# An *in vivo* assessment of the cholesterol-lowering efficacy of *Lactobacillus plantarum* ECGC 13110402 in normal to mildly hypercholesterolaemic adults

**DOI:** 10.1371/journal.pone.0187964

**Published:** 2017-12-11

**Authors:** Adele Costabile, Ivan Buttarazzi, Sofia Kolida, Sara Quercia, Jessica Baldini, Jonathan R. Swann, Patrizia Brigidi, Glenn R. Gibson

**Affiliations:** 1 Health Sciences Research Centre, Life Sciences Department, Whitelands College, University of Roehampton, London, United Kingdom; 2 Department of Food and Nutritional Sciences, University of Reading, Reading, United Kingdom; 3 Optibiotix Health plc, Innovation Centre, Innovation Way, Heslington, York, United Kingdom; 4 Department of Pharmacy and Biotechnology, Alma Mater Studiorum, University of Bologna, Bologna, Italy; 5 Division of Computational and Systems Medicine, Imperial College, London, United Kingdom; Kurume University School of Medicine, JAPAN

## Abstract

Coronary heart disease (CHD) is one of the major causes of death and disability in industrialised countries, with elevated blood cholesterol an established risk factor. Total plasma cholesterol reduction in populations suffering from primary hypercholesterolemia may lower CHD incidence. This study investigated the cholesterol reducing capacity of *Lactobacillus plantarum* ECGC 13110402, a strain selected for its high bile salt hydrolase activity, in 49 normal to mildly hypercholesterolaemic adults. Primary efficacy outcomes included effect on blood lipids (total cholesterol (TC), low density lipoproteins (LDL-C), high density lipoproteins (HDL-C) and triacylgycerides (TAG), inflammatory biomarkers and occurrence/severity of gastrointestinal side effects to establish safety and tolerance of the intervention. Secondary outcomes included blood pressure, immune biomarkers, gut microbiota characterisation and metabonome changes. The study was run in a parallel, double blind, placebo controlled, randomised design in which the active group ingested 2x10^9^ CFU encapsulated *Lactobacillus plantarum* ECGC 13110402 twice daily. Daily ingestion of the active treatment resulted in a statistically significant reduction in LDL-C in volunteers with baseline TC<5mM during the 0–12 week period (13.9%, P = 0.030), a significant reduction in TC in volunteers with baseline TC≥6mM in the 0–6 week period (37.6%, P = 0.045), a significant decrease in TAG (53.9% P = 0.030) and an increase in HDL-C (14.7%, P = 0.007) in the over 60 years population in the 6–12 week period. A statistically significant reduction in systolic blood pressure was also observed across the active study group in the 6-12-week period (6.6%, P = 0.003). No impact on gastrointestinal function and side effects was observed during the study. Similar to blood and urine metabonomic analyses, faecal metagenomics did not reveal significant changes upon active or placebo intake. The results of this study suggest that *Lactobacillus plantarum* ECGC 13110402 is a well-tolerated, natural probiotic, that may be used as an alternative or supplement to existing treatments to reduce cardiovascular risk.

**Trial registration:** Clinical trials.gov NCT03263104

## Introduction

Coronary heart disease (CHD) is one of the major causes of death and disability in industrialised countries [1]. The World Health Organization (WHO) predicts that by the year 2020, up to 40% of all human deaths will be related to cardiovascular diseases. The most common form of CHD is coronary artery disease (CAD) and is now the leading cause of death globally, as reported by the WHO, accounting for 7.25 million deaths a year. Results from epidemiological and clinical studies indicate a positive correlation between elevated total serum cholesterol levels, mainly reflecting the low density lipoprotein-cholesterol fraction (LDL-C), and risk of CHD onset [2]. Evidence demonstrates a log-linear relationship between increasing LDL-C concentrations and relative risk for CAD. Epidemiological studies and clinical trials for cholesterol-lowering approaches have confirmed this relationship, showing an almost identical pattern of association. As such, there is interest in addressing serum cholesterol and other blood lipids [3], to reduce cardiovascular risk with LDL-C being a primary target for the management of lipid levels in many national and international guidelines.

Although dietary strategies for prevention of CHD are the first line of treatment, they are based on adherence to low cholesterol/low saturated fat diets, which may be effective, but are difficult to maintain on a long-term basis with efficacy diminishing over time [4,5]. Pharmacological approaches for cholesterol management include fibric acid derivatives (fibrates), nicotinic acid, bile acid sequestrants (BAS), oestrogen replacement therapy and statins. Statin therapy is currently the most commonly used approach for cholesterol management, and while it has been shown to reduce the incidence of first or recurrent CHD events, a high patient population fail to sustain statin monotherapy, with noncompliance for primary (25.4%), chronic (36.1%), and acute coronary disease (40.1%) after 2 years of treatment of treatment [6]. Consequently, there is a gap between patients’ requirements and clinical practice, with limitations of current approaches increasing interest in non-drug therapies to improve blood cholesterol profiles. This includes probiotics with modes of action that have the potential to improve outcomes in patients with CHD with no or little risk of side effects.

Some studies have indicated the potential of probiotics and fermented dairy foods in cholesterol management. However, several studies have reported little or no effect. These contradictory results have largely been attributed to heterogeneity in study design and variability in efficacy of specific microbial strains [7]. This often reflects a lack of scientific rationale behind the choice of probiotics used in many studies. As the use of probiotics moves from general welfare to demonstrable specific health benefits, there is a growing need to identify reliable microbial strains and their mechanism(s) of action. As the pivotal role of gut microbiota (GM) in regulating lipid absorption in the intestine is well recognised, it is crucial to investigate the impact of probiotics on overall structure of the microbial ecosystem. There are a number possible mechanisms for cholesterol removal by probiotics including: adsorption of cholesterol to cellular surfaces, assimilation of cholesterol into cell membranes, cholesterol reductase activity and the deconjugation of bile acids via bile salt hydrolase (BSH) [8,9]. One area of growing interest is the potential of BSH probiotics to reduce cholesterol. BSH active probiotics have been shown to increase intraluminal bile salt deconjugation, resulting in increased levels of circulating deconjugated bile acids. Once deconjugated, bile acids are less soluble and are absorbed in the intestine to be excreted in faeces. Cholesterol is then used for *de novo* bile acid synthesis in a homeostatic response, resulting in lowering of serum cholesterol [10]. A systematic review [11] of 1197 publications, examining the role of bile acids in reducing the metabolic complications of obesity post bariatric surgery, concluded that there was good evidence to support the role of bile acids in lipid and cholesterol metabolism. This suggests that regulation of bile acids using BSH producing probiotics could serve as a safe and natural approach to modify blood lipid levels and reduce cardiovascular risk.

To identify strains with cholesterol reducing potential a systematic screen of phylogenetically diverse microbial strains was carried out. *Lactobacillus plantarum* ECGC 13110402 (dairy isolate) was selected as it demonstrated high *in vitro* BSH activity, cholesterol removal potential, resistance to gastric, pancreatic, and bile acids and high survivability on freeze drying. The aim of this human volunteer study was to establish tolerance, and the extent of the cholesterol lowering potential of *Lactobacillus plantarum* ECGC 13110402 in 49, healthy, normal to mildly hypercholesterolaemic adults (30–65 years old). Primary efficacy outcomes included effect on blood lipids (total cholesterol, TC; low density lipoproteins, LDL-C; high density lipoproteins, HDL-C; and triacylgycerides, TAG) inflammatory biomarkers and occurrence/ severity of gastrointestinal side effects to establish safety and tolerance of the intervention. Secondary outcomes included blood pressure, immune biomarkers, gut microbiota characterisation and metabonome changes. The 12-week intervention was conducted in a parallel, double blind, placebo controlled, randomised design and concluded with a four-week washout period.

## Materials and methods

### Selection and characteristics of study population

**Fig 1:**
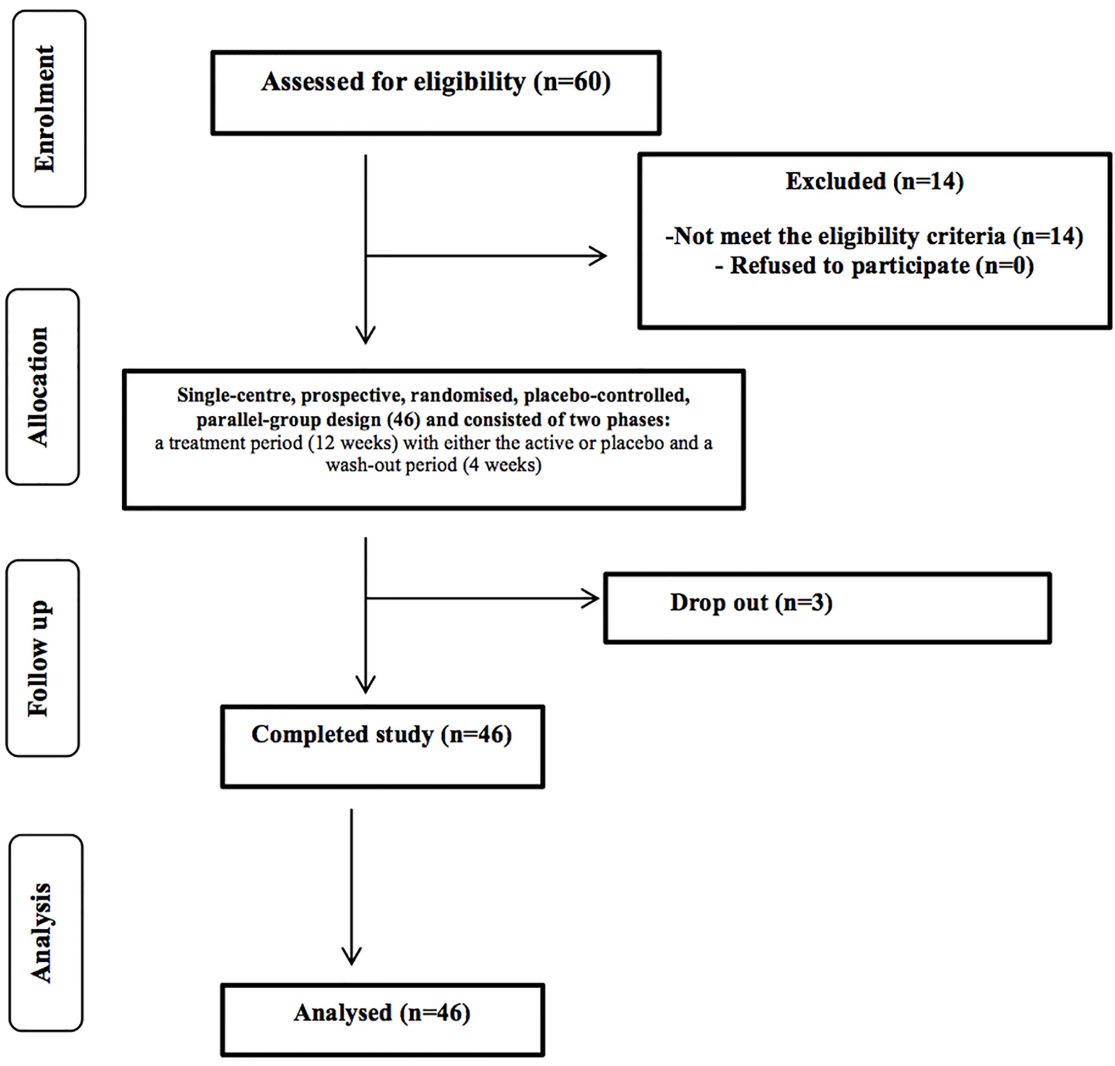
Flow of the study participants through the intervention.

The study was registered as a clinical trial (clinical trials.gov ID: NCT03263104) and conducted according to the Declaration of Helsinki following Good Clinical Practice (GCP). The study was carried out according to the Helsinki declaration and was approved by the University of Reading Research Ethics Committee (UREC 15/06 on 28.01.2015). Written informed consent was obtained from all subjects prior to commencement of selection. Inclusion criteria were: males and females from 18 to 50 years of age; BMI 18.5 to 29.9 kg/m^2^; total cholesterol (TC) between 200 and 300 mg/dl (5.16 and 7.64 mmol/L). General health status was assessed using a pre-study medical questionnaire and putative volunteers were excluded if they fulfilled any of the following: suffering from chronic gastrointestinal complaints (including chronic constipation, diarrhoea or Irritable Bowel Syndrome), diabetes or anaemia; requirement to take long-term medications active on the gastrointestinal tract, treatment of cardio-vascular disease, or any other long-term medication; high blood cholesterol or use of cholesterol lowering drugs/functional foods; history of drug or alcohol misuse or alcohol consumption exceeding 14 and 21 units/week for females and males respectively; those suffering with any allergies to medication or food; smokers, and those on weight reducing diets. Females who were either planning pregnancy within six months from the start of the study, lactating, or had given birth within the preceding six months were also excluded. Specified timed exclusion criteria included use of antibiotics within six months preceding the study, participation in any probiotic, prebiotic or laxative study or intake of an experimental drug four weeks prior to the study start. A total of 70 potential healthy volunteers (aged from 18 to 50 years) were contacted from the University of Reading and surrounding area through the Hugh Sinclair Unit of Human Nutrition volunteer database, and through advertisements within the local community between January 2015 and February 2015. Of the first 68 responded, 60 were screened and we recruited 49 volunteers; 46 participants completed the study with 3 dropout due to personal circumstances (Fig 1). The participants (gender: 34 females, 15 males; mean age: 51.5 (50.31 male, 52.70 female), range 30 to 65 years; body mass index (BMI): male 25.68, female 27.19, total average mean 26.43 kg/m^2^, range 19.6 to 40.2 kg/m^2^) were randomly assigned into two treatment groups: placebo and active. An Excel-based covariate adaptive randomisation program was used to enter two intervention arms randomized the volunteers, stratified by age, gender and BMI. Both treatments were blinded by the manufacturers and were delivered in identical blister packed capsules. The study was powered to provide 80% statistical power (MGH Biostatistics Hedwig Software) based on an average (±SD) log change: 0.45 ± 0.4 for total cholesterol (TC) on the basis of the data from previous intervention studies in human volunteers conducted on blood lipids. Given these calculations, 49 participants (to allow for 15% attrition) will be required to detect differences. A treatment difference at a two-sided 0.05 significance level.

### Requirements for diet and medication during study

Intake of the following foods or substances was not permitted: prebiotic supplements, probiotics, statins, drugs active on gastrointestinal motility, antibiotic treatment or any class of laxative. Any medication taken was recorded in diaries. Volunteers were instructed not to alter their usual diet or fluid intake during the trial period.

#### *Lactobacillus plantarum* ECGC 13110402 selection

The BSH activity and bile resistance of 353 phylogenetically diverse *Lactobacillus* strains was determined under anaerobic conditions on 96 well plates. A bile salt solution (5μl containing each of (w/v): 0.5% glycodeoxycholic acid, 0.5% taurodeoxycholic acid, 0.5% oxgal, 2.0% oxgal) was spotted on each well and each test was scored for growth after 24 and 72h. Promising results were obtained for 45 strains, the BSH activity of which was quantified using classical plate assays. The 24 *Lactobacillus* strains presenting the highest activities were selected to perform cholesterol assimilation assays. MRS media containing 20 mg/l cholesterol and 0.4% (w/v) oxgal were used to grow the *Lactobacillus* strains anaerobically for 24h at 37°C. *Lactobacillus* cells were subsequently separated from the medium through centrifugation and the medium was frozen at -25°C until further processing. After thawing the samples, cholesterol was isolated from the medium at room temperature by lipid extraction. Supernatant (3 ml) was shaken vigorously with 3 ml ether-heptane (1:1, v/v) and 1 ml ethanol (≥99.8%), then centrifuged for 5 minutes at 3000 g. The upper layer of ether/heptane was collected in a glass tube and the extraction repeated twice with 1.5 ml ether/heptane and 0.5 ml ethanol. Content of the collected lipid extraction tube was dried under nitrogen at approximately 40°C. The fat components were then dissolved in heptane in an ultrasonic bath for 5 minutes. A small amount of dried sodium sulphate was added to the solution to bind any water present. Following centrifugation, 1.0 μl was injected onto a Capillary GC Column (Agilent: VF-5ht UltiMetal 30 m x 0.32mm ID DF = 0.10μm including 2m x 0.53mm ID retention gap) with a flame ionisation detector. Calculation of the cholesterol content was done using a standard solution of cholesterol in heptane.

#### Study design

The study was carried out in a single-centre, prospective, randomised, placebo-controlled, parallel-group design and consisted of two phases: a treatment period (12 weeks) with either the active or placebo and a wash-out period (4 weeks). Following a screening visit which ensured adherence to inclusion criteria, the study included a baseline, midpoint, endpoint (week 6 and 12, respectively) and washout visit (week 16).

Participants were asked to take the products twice daily, before breakfast and dinner, and were advised not to change their regular diet or physical activity throughout the trial period. Each participant was asked to consume the treatment, which comprised of *Lactobacillus plantarum* ECGC 13110402 at a concentration of 2x10^9^ CFU (0.1 g) twice daily in capsular format (vegetable) with the addition of filling carrier (0.12 g; 30% w/v maltodextrin and 5% w/v sucrose), or the placebo (0.12 g; 30% w/v maltodextrin and 5% w/v sucrose). All participants were advised on product storage conditions to ensure product consistency throughout the period of the study. The probiotic was produced by CSL (Centro Sperimentale del Latte, Lodi, Italy). Active and placebo formulae were blended, encapsulated and blind packaged in blister packs by Nutrilinea srl (Gallarate, Italy).

#### Anthropometric measurements and blood pressure

Study participant anthropometric parameters were measured during all visits. These included weight, Body Mass Index (BMI) and body fat percentage (Tanita^®^ BC-418 Segmental Body Composition Analyzer, Scalesmart UK). In addition, height and waist circumference were also measured. Blood pressure readings were obtained in triplicate during each visit using an electric sphygmomanometer blood pressure monitor (CONTEC08E, UK).

#### Blood, urine and stool sample collection

For all visits, freshly voided faecal samples and first pass urine were collected and stored at -80°C until further analysis. For each volunteer, blood samples were collected by a trained phlebotomist into one 10 ml EDTA tube (BD Vacutainer EDTA Tube, BD, Cowley, Oxon, UK) for fasting TAG, TC and HDL-C analysis; into one 2 ml fluoride/oxalate tube (BD Vacutainer Fluoride/Oxalate Tube) for fasting glucose analysis. Following collection, all samples were kept on ice until centrifugation. Plasma samples were recovered by centrifugation at 1700 g for 10 min, dispensed into 1.5 ml microcentrifuge tubes and frozen at -20°C within 1 h from the collection. Plasma samples were defrosted and centrifuged for 5 min at 1500 g prior to analysis.

#### Determination of plasma TAG, total cholesterol and HDL-cholesterol concentrations

Plasma TAG, TC and HDL-C concentrations were determined using the AU5800 Clinical Chemistry System (Beckman Coulter, Buckinghamshire, UK). Test kits (IL Test triacylglycerols, IL Test cholesterol, IL Test HDL cholesterol) supplied by Beckman Coulter UK and were used according to instructions for the determination of plasma TAG, TC, HDL-C respectively.

#### Inflammation/ immune biomarkers

Inflammatory biomarkers, interleukin 6 (IL-6), tumour necrosis factor alpha (TNF-α), c reactive protein (CRP) and interleukin 10 (IL-10) were measured by Enzyme-Linked ImmunoSorbent Assay (ELISA) using appropriate specific reagent kits (Quantikine^®^ ELISA, R&D Systems Inc., Barton, UK).

Serum samples were thawed on a roller mixer. Calibrators and reagents were prepared according to manufacter’s instructions. A 25 μl aliquot of serum, calibrator or control was pipetted onto a 96-well plate and 100 μl anti-cytokine antibody, horseradish peroxidase, conjugate added. The plate was shaken for 1 h at room temperature. Following incubation, the plate was washed three times with wash buffer. A 100 ml aliquot of substrate solution was added to each well and the plate shaken for 10 min at room temperature. The reaction was stopped by addition of 100 μl stop solution and the plate read immediately at 450 nm using an automated ELISA plate spectrophotometer (Tecan GENios; Process Analysis and Automation Ltd. Farnborough, Hants, UK). Cytokine concentrations of the samples and the quality controls were determined automatically by reading from the standard curve using Magellan (Version 5.01) computer software.

#### ^1^H nuclear magnetic resonance (NMR) spectroscopy for urine and serum samples

Metabonomic analysis was performed via Nuclear Magnetic Resonance (NMR) spectroscopy at Imperial College, London (on urine and serum samples) for metabolites and bile salt quantification. The NMR urine samples were run sequentially to optimise data acquisition and minimise natural variation. All samples from one experiment were run as one batch to preserve sample integrity. Samples were prepared with deuterated phosphate buffer (28.86 g/L disodium phosphate, 5.25 g/L monosodium phosphate, 0.172 g/L sodium 3 (trimethylsilyl) propionate-d4 (TSP) (1 mM) and 0.193 g/L sodium azide in 900 ml deuterium oxide (D_2_O) (v/v) and 100 ml H_2_O). Urine samples run by NMR were defrosted from -80°C and vortexed to ensure a homogenous sample. Each urine sample (400 μl) was then placed into a microfuge tube and 200 μl of phosphate buffer added, vortexed and centrifuged at 8000 g for 10 min before 550 μl of supernatant was transferred to a 5 mm NMR tube. TSP was used as an internal standard for NMR spectrum calibration and sodium azide was added to prevent microbial growth whilst samples were awaiting NMR analysis. All samples were analysed on a Bruker Ultrashield Plus NMR Spectrometer at 300 K operating at 700.13 MHz for ^1^H observation. A standard one-dimensional NMR spectrum was acquired with water peak suppression using a standard pulse sequence (recycle delay-90°-*t*1-90°-*t*m-90°-acquisition)-Noesypr1D. The recycle delay was set at 2 seconds and the mixing time (*t*m) at 100 milliseconds.

For plasma samples, 30 μl of sample was combined with 30 μl saline buffer (no TSP) and 50 μl transferred to a capillary NMR tube. Plasma samples were acquired using the same standard pre-saturation pulse sequence as for urine and hepatic samples followed by Carr-Purcell Meiboom Gill (CPMG) spin-echo pulse program with 8 dummy scans followed by 128 scans collected in 64 K data points, with a spectral width of 20 ppm and an acquisition time per scan of 1.36 seconds. The mixing time was 100 milliseconds and the recycle dela 2 seconds. All ^1^H NMR spectra were processed by TopSpin 3.1.5 (Bruker^™^) algorithms for phase correction, calibration of chemical shift by setting TSP to zero ppm and baseline correction to allow comparison of spectral data by multivariate data analysis.

#### Microbial DNA extraction from faeces

Total bacterial DNA was extracted from 250 mg of faecal sample using QIAamp DNA Stool Mini Kit (QIAGEN, UK) with modified protocol [12]. The DNAs samples were resuspended in 100 μl of TE buffer and treated with 2 μl of DNase-free RNase (10 mg/ml) at 37°C for 15 min. Proteins were removed by treatment with 15 μl of proteinase K at 70°C for 10 min. DNA was subsequently purified using QIAamp Mini Spin columns (QIAGEN) following the manufacturer’s instructions. Final DNA concentration was quantified by using NanoDrop ND-1000 (NanoDrop Technologies, Wilmington, DE).

#### 16S rRNA gene amplification via next generation sequencing (NGS) and bioinformatics analysis

For NGS analysis, the V3-V4 region of the 16S rRNA gene was PCR amplified in 50 μL final volume mix containing 25 ng of microbial DNA, 2X KAPA HiFi HotStart ReadyMix (KAPA Biosystems, Resnova, Rome, Italy), and 200 nmol/L of S-D-Bact-0341-b-S-17/S-D-Bact-0785-a-A-21 primers carrying Illumina overhang adapter sequences. The amplification cycle consisted of an initial denaturation of 3 min at 95°C 25 cycles of 30 sec at 95°C for denaturation, 30 sec at 55°C for annealing, and 30 sec at 72°C for elongation, with a final extension step of 5 min at 72°C and was performed with a Biometra Thermal Cycler T Gradient (Biometra). Magnetic bead-based clean-up system (Agencourt AMPure XP; Beckman Coulter, Brea, CA) was used for purification of the 460 bp amplicons, which were then used to prepare indexed libraries using a limited-cycle PCR using Nextera Technology. Libraries were purified and successively pooled at equimolar concentrations (4nM), denatured and diluted to 6 pmol/L. Samples were sequenced on Illumina MiSeq platform using a 2×300 bp paired end protocol, according to the manufacturer’s instructions (Illumina, San Diego, CA). Paired-end reads, obtained by sequencing, were analysed using the QIIME pipeline [13] and PANDAseq [14] and the Greengenes database [15] was utilised for taxonomic assignment. The chimera filtering was performed using UCLUST, which removes sigleton OTUs [16].

### Statistical analysis

An initial set of analyses examined the demographic and outcome variables at baseline to ensure that the two groups were well matched. Continuous variables were analysed using the unpaired t-test, whilst the Chi-square test was used for the categorical variables. The change in outcomes over four pre-determined study periods was examined. Periods examined were: baseline to 6 weeks; 6 weeks to 12 weeks; baseline to 12 weeks and 12 weeks to 16 weeks. Analyses were performed using analysis of covariance (ANCOVA) via Stata (version 13.2). The latter time-point was used as the outcome variable, with the earlier time-point considered as a covariate. Separate ANCOVA analyses were performed for each time period. The assumptions of the models were examined by checking the residuals for normality and for their relationship with the predicted values. The data were complete for most patients and for most outcomes. Missing values were omitted from the analyses.

The effect of treatment on lipid profiles, blood pressure, inflammatory/immune biomarkers, gastrointestinal symptoms and anthropometric measurements was first considered on the total study population. Placebo and active groups were additionally compared in patient subgroups, stratifying according to baseline TC levels: normal baseline TC (N-TC) <5mM, mildly elevated baseline TC (M-TC) 5–5.9mM, high baseline TC (H-TC) ≥6.0mM), gender (male, female) and age (<50yrs, 50-59yrs, ≥60yrs).

For H^1^NMR, multivariate statistical data analyses (PCA and OPLS-DA) were performed in Matlab using statistical tools from the Korrigan Toolbox (Korrigan Sciences Ltd, UK) and in house scripts from the University of Reading (UK).

Biostatistic analyses of metagenomic data was performed with R (https://www.r-project.org/) and Microsoft Excel. Principal Coordinate Analysis (PCoA) was conducted to explore and visualise similarities among samples using weighted and unweighted UniFrac metric [17].

## Results

### *Lactobacillus plantarum* ECGC 13110402 selection

Following systematic *in vitro* screening of a phylogeniticaly diverse collection of *Lactobacillus* strains, *Lactobacillus plantarum* ECGC 13110402 was identified as the most promising for combining high BSH activity (2.6. μmol/hr/e10cells), the ability to reduce cholesterol in *vitro* by 77.9%, high preferential resistance to gastric, pancreatic, and bile acids and high survivability on freeze drying. *Lactobacillus plantarum* ECGC 13110402 was manufactured, encapsulated, packaged in blister packs for use in this human intervention study.

### Baseline demographic variables and volunteer stratification

Demographic parameters for the active (n = 23) and placebo (n = 23) groups were examined at baseline (Table 1). No statistically significant differences were found in baseline lipid profiles, weight, BMI, waist circumference, systolic and diastolic blood pressure, age and gender. Table 2 shows volunteer stratification into subgroups based on their baseline TC levels, age and gender.

**Table 1:** Demographic and baseline characteristics of human intervention study participants in the active (*Lactobacillus plantarum* ECGC 13110402) and placebo treatment groups.

Variable	Placebo (n = 23) Mean (SD)	Active (n = 23) Mean (SD)	P-value
Age (years)	52.0 (8.4)	52.3 (10.7)	0.89
Gender (*) Female	14 (61%)	18 (78%)	0.20
Male	9 (39%)	5 (22%)	
Total cholesterol (mM)	5.22 (0.92)	5.10 (0.71)	0.62
HDL cholesterol (mM)	1.24 (0.31)	1.40 (0.35)	0.10
LDL cholesterol (mM)	3.44 (0.76)	3.20 (0.68)	0.28
Triglycerides (mM)	1.18 (0.45)	1.11 (0.46)	0.61
Weight (Kg)	79.2 (16.5)	72.1 (12.0)	0.10
BMI (Kg/m^2^)	26.8 (5.0)	26.7 (3.7)	0.96
Waist (cm)	92.3 (13.5)	89.6 (12.0)	0.49
Systolic BP (mm Hg)	118.7 (16.0)	119.2 (13.2)	0.73
Diastolic BP (mm Hg)	71.0 (12.2)	73.0 (8.0)	0.52

(*) Number (%) reported.

BMI: body mass index; BP: blood pressure

**Table 2:** Human intervention study volunteer stratification into groups according to baseline total cholesterol, age and gender in the active (*Lactobacillus plantarum* ECGC 13110402) and placebo treatment groups.

	Total cholesterol	Age	Gender
	**<5mM (N-TC)**	**<50yrs**	**Male**
**Active**	12	9	5
**Placebo**	11	7	9
	**5–5.9mM (M-TC)**	**50-59yrs**	**Female**
**Active**	8	6	18
**Placebo**	9	12	14
	**≥6.0mM (H-TC)**	**≥60yrs**	
**Active**	3	8	
**Placebo**	3	4	

TC: total cholesterol; Normal baseline TC: N-TC:<5mM; mildly elevated baseline TC: M-TC: 5–5.9mM, high baseline TC: H-TC: ≥6.0mM)

### Anthropometric data and blood pressure

Treatment with *Lactobacillus plantarum* ECGC 13110402 had no statistically significant impact on body weight, BMI or waist circumference in the baseline to 12-week study period (Table 3). The study groups were found to vary significantly (P = 0.003; -1.8 Change Mean (SD) [range], -0.7% Change Mean (SD) [range], -0.6 Group Difference Mean (95% Cl) in their systolic blood pressure for the period from 6 to 12 weeks (Table 4). There was increase in the placebo group, but a small decrease in the active group. The values at 12 weeks were found to be 6 mmHg (5.1%) lower in the active group than in the placebo group.

**Table 3:** Change in anthropometric measurements and blood pressure in all study participants between baseline and 12 weeks in the active (*Lactobacillus plantarum* ECGC 13110402) and placebo treatment groups.

Outcome	Group	N	Baseline Mean (SD)	12 weeks Mean (SD)	Change Mean (SD) [range]	% Change Mean (SD) [range]	Group Difference Mean (95% CI)	P-value
**Weight**	Placebo	23	79.2 (16.5)	79.3 (16.8)	0.2 (1.7) [-2.6, 3.5]	0.1 (2.1) [-3.3, 4.7]	0	
**(Kg)**	Active	23	72.1 (12.0)	72.8 (12.6)	0.7 (1.7) [-2.6, 3.8]	0.9 (2.2) [-2.8, 4.9]	0.7 (-0.3, 1.7)	0.18
**BMI**	Placebo	23	26.8 (5.0)	27.0 (5.2)	0.3 (1.3) [-3.1, 4.2]	0.9 (4.7) [-9.3, 15.5]	0	
**(Kg/m**^**2**^**)**	Active	23	26.7 (3.7)	27.2 (4.0)	0.5 (0.9) [-1.1, 3.3]	2.0 (3.3) [-3.9, 11.8]	0.3 (-0.4, 1.0)	0.41
**Waist**	Placebo	23	92.3 (13.5)	90.5 (13.8)	-1.8 (6.4) [-14, 12]	-1.8 (6.8) [-17.3, 12.9]	0	
**(cm)**	Active	23	89.6 (12.0)	89.1 (11.0)	-0.5 (5.7) [-13, 13]	-0.2 (6.7) [-13.0, 16.3]	0.9 (-2.6, 4.4)	0.61
**Systolic BP**	Placebo	23	117.7 (16.0)	122.3 (11.4)	4.7 (11.0) [-13, 28]	4.9 (10.3) [-11.4, 31.1]	0	
**(mm Hg)**	Active	22	119.2 (13.2)	119.7 (13.0)	0.5 (8.9) [-19, 21]	0.7 (7.2) [-13.4, 15.8]	-3.6 (-8.6, 1.4)	0.15
**Diastolic BP**	Placebo	23	71.0 (12.2)	73.5 (8.2)	2.4 (9.0) [-15, 18]	5.0 (13.6) [-14.4, 30.5]	0	
**(mm Hg)**	Active	22	73.0 (8.0)	73.0 (8.2)	0.0 (5.9) [-9, 13]	0.3 (8.4) [-10.5, 20.3]	-1.6 (-5.2, 2.1)	0.39

BMI: body mass index; BP: blood pressure; N: number of participants

**Table 4:** Change in anthropometric measurements and blood pressure in all study participants between 6 and 12 weeks in the active (*Lactobacillus plantarum* ECGC 13110402) and placebo treatment groups.

Outcome	Group	N	6 weeks Mean (SD)	12 weeks Mean (SD)	Change Mean (SD) [range]	% Change Mean (SD) [range]	Group Difference Mean (95% CI)	P-value
**Weight**	**Placebo**	23	79.4 (16.2)	79.3 (16.8)	0.0 (1.3) [-3.0, 2.8]	-0.2 (1.7) [-3.8, 2.8]	0	
**(Kg)**	**Active**	23	72.6 (12.5)	72.8 (12.6)	0.2 (1.1) [-2.6, 2.1]	0.2 (1.5) [-3.8, 3.3]	0.3 (-0.4, 1.1)	0.34
**BMI**	**Placebo**	23	26.9 (5.0)	27.0 (5.2)	0.1 (1.0) [-1.2, 3.7]	0.5 (3.5) [-4.8, 13.4]	0	
**(Kg/m**^**2**^**)**	**Active**	23	26.9 (3.8)	27.2 (4.0)	0.3 (0.8) [-0.7, 3.3]	1.0 (2.9) [-2.5, 11.8]	0.1 (-0.4, 0.7)	0.57
**Waist**	**Placebo**	23	91.3 (13.3)	90.5 (13.8)	-0.8 (2.7) [-8, 4]	-0.9 (2.8) [-7.1, 3.9]	0	
**(cm)**	**Active**	23	90.0 (11.3)	89.1 (11.0)	-0.9 (3.5) [-13, 6]	-0.9 (3.9) [-13.0, 8.1]	-0.1 (-2.0, 1.8)	0.90
**Systolic**	**Placebo**	23	116.2 (16.0)	122.3 (11.4)	6.1 (7.8) [-17, 22]	5.9 (6.7) [-9.9, 22.9]	0	
**(mm Hg)**	**Active**	22	121.0 (18.4)	119.7 (13.0)	-1.8 (10.5) [-35, 11]	-0.7 (7.4) [-22.2, 11.3]	-6.0 (-9.9, -2.1)	**0.003**
**Diastolic**	**Placebo**	23	72.3 (9.5)	73.5 (8.2)	1.2 (5.6) [-15, 13]	2.1 (7.5) [-14.4, 21.3]	0	
**(mm Hg)**	**Active**	22	71.9 (9.6)	73.0 (8.2)	0.6 (5.6) [-11, 9]	1.4 (7.9) [-13.6, 15.7]	-0.7 (-3.6, 2.2)	0.62

BMI: body mass index; BP: blood pressure; N: number of participants

### Lipid parameters

#### Total cholesterol (TC)

When compared to the placebo group, TC from baseline to 12 weeks showed a consistent pattern of decrease across all active treatment groups, however this was not statistically significant (Tables 5–7). In the baseline to mid study point period (0–6 weeks) statistically significant reductions of 2.44mmol/l were observed in total cholesterol, corresponding to a 36.7% reduction (P = 0.045; -0.50 Change Mean (SD) [range], -7.8% Change Mean (SD) [range],-2.44 Group Difference Mean (95% Cl) in the H-TC group (Table 8). However, the population size of this group was very small (n = 3 placebo/3 active) and low numbers can be highly affected by individual changes despite its statistical significance. No group relevance should therefore be attributed to this effect. No significant effect of gender on was identified on TC.

**Table 5:** Lipid parameters expressed in mM in all study participants between baseline and 12 weeks in the active (*Lactobacillus plantarum* ECGC 13110402) and placebo treatment groups.

Outcome	Group	N	Baseline Mean (SD)	12 weeks Mean (SD)	Change Mean (SD) [range]	% Change Mean (SD) [range]	Group Difference Mean (95% CI)	P-value
**TC**	Placebo	23	5.22 (0.92)	5.33 (0.84)	0.11 (0.66) [-1.0, 1.4]	3.1 (13.4) [-14.6, 34.1]	0	
	Active	23	5.10 (0.71)	5.12 (0.87)	0.02 (0.56) [-1.3, 1.2]	0.6 (10.5) [-22.4, 23.3]	-0.12 (-0.47, 0.24)	0.51
**HDL-C**	Placebo	23	1.24 (0.31)	1.24 (0.29)	0.00 (0.17) [-0.2, 0.5]	1.5 (17.0) [-14.3, 62.5]	0	
	Active	23	1.40 (0.35)	1.46 (0.42)	0.06 (0.15) [-0.1, 0.5]	3.4 (10.5) [-12.5, 33.3]	0.06 (-0.04, 0.16)	0.023
**LDL-C**	Placebo	23	3.44 (0.76)	3.54 (0.70)	0.10 (0.62) [-0.9, 1.3]	4.9 (19.5) [-22.9, 52.0]	0	
	Active	23	3.20 (0.69)	3.13 (0.78)	-0.07 (0.53) [-1.3, 1.0]	-1.4 (15.6) [-36.4, 26.3]	-0.24 (-0.56, 0.09)	0.15
**TAG**	Placebo	23	1.18 (0.45)	1.20 (0.39)	0.03 (0.41) [-0.9, 0.6]	10.9 (35.7) [-52.9. 83.3]	0	
	Active	23	1.11 (0.46)	1.15 (0.65)	0.04 (0.36) [-0.5, 0.8]	3.6 (34.8) [-55.6, 114.3]	0.01 (-0.22, 0.23)	0.96

TC: total cholesterol; HDL-C: High-density lipoprotein cholesterol; LDL-C: low-density lipoprotein cholesterol; TAG: triacylglycerol; N: number of participants

**Table 6:** Lipid parameters expressed in mM in the normal total cholesterol group (TC <5mM) between baseline and 12 weeks intervention study in the active (*Lactobacillus plantarum* ECGC 13110402) and placebo treatment groups.

Outcome	Group	N	Baseline Mean (SD)	12 weeks Mean (SD)	Change Mean (SD) [range]	% Change Mean (SD) [range]	Group Difference Mean (95% CI)	P-value
**TC**	**Placebo**	11	4.50 (0.28)	4.79 (0.43)	0.29 (0.60) [-0.7, 1.4]	7.1 (13.7) [-14.6, 34.1]	0	
	**Active**	12	4.53 (0.33)	4.61 (0.42)	0.08 (0.38) [-0.5, 1.0]	2.0 (8.5) [-10.9, 23.3]	-0.19 (-0.56, 0.19)	0.31
**HDL-C**	**Placebo**	11	1.09 (0.28)	1.11 (0.23)	0.02 (0.19) [-0.2, 0.5]	4.5 (22.3) [-14.3, 62.5]	0	
	**Active**	12	1.34 (0.30)	1.44 (0.42)	0.10 (0.19) [-0.1, 0.5]	6.2 (12.9) [-12.5, 33.3]	0.09 (-0.10, 0.27)	0.33
**LDL-C**	**Placebo**	11	2.88 (0.33)	3.15 (0.47)	0.26 (0.59) [-0.8, 1.3]	10.5 (21.0) [-22.9, 52.0]	0	
	**Active**	12	2.71 (0.31)	2.72 (0.28)	0.02 (0.30) [-0.6, 0.6]	1.3 (11.7) [-21.4, 26.1]	-0.39 (-0.74, -0.04)	**0.03**
**TAG**	**Placebo**	11	1.14 (0.51)	1.15 (0.38)	0.02 (0.41) [-0.9, 0.5]	12.3 (37.3) [-52.9, 62.5]	0	
	**Active**	12	1.08 (0.39)	0.97 (0.35)	-0.12 (0.25) [-0.5, 0.2]	-8.1 (25.4) [-55.6, 28.6]	-0.01 (-0.22, 0.23)	0.96

TC: total cholesterol; HDL: High-density lipoprotein cholesterol; LDL: low-density lipoprotein cholesterol; TAG: triacylglycerol; N: number of participants

**Table 7:** Lipid parameters expressed in mM in the mildly elevated total cholesterol group (TC <5–5.9mmol mM) between baseline and 12 weeks intervention study in the active (*Lactobacillus plantarum* ECGC 13110402) and placebo treatment groups.

Outcome	Group	N	Baseline Mean (SD)	12 weeks Mean (SD)	Change Mean (SD) [range]	% Change Mean (SD) [range]	Group Difference Mean (95% CI)	P-value
**TC**	**Placebo**	9	5.48 (0.25)	5.52 (0.64)	0.04 (0.74) [-0.8, 1.2]	1.1 (13.8) [-14.5, 22.2]	0	
	**Active**	8	5.55 (0.24)	5.25 (0.54)	-0.30 (0.64) [-1.3, 0.6]	-5.2 (11.4) [-22.4, 11.8]	-0.23 (-0.87, 0.40)	0.44
**HDL-C**	**Placebo**	9	1.30 (0.26)	1.31 (0.33)	0.01 (0.15) [-0.2, 0.2]	0.3 (11.2) [-12.5, 15.4]	0	
	**Active**	8	1.50 (0.47)	1.51 (0.49)	0.01 (0.1) [-0.1, 0.2]	0.6 (6.4) [-8.3, 11.8]	-0.01 (-0.15, 0.14)	0.91
**LDL-C**	**Placebo**	9	3.61 (0.27)	3.63 (0.54)	0.02 (0.67) [-0.8, 1.1]	1.5 (19.2) [-21.1, 33.3]	0	
	**Active**	8	3.55 (0.40)	3.16 (0.58)	-0.39 (0.65) [-1.3, 0.6]	-10.2 (18.3) [-36.4, 18.8	-0.47 (-1.08, 0.13)	0.11
**TAG**	**Placebo**	9	1.24 (0.42)	1.29 (0.48)	0.04 (0.46) [-0.9, 0.6]	10.7 (39.3) [-52.9, 83.3]	0	
	**Active**	8	1.11 (0.58)	1.26 (0.92)	0.15 (0.38) [-0.3, 0.7]	5.9 (26.4) [-33.3, 41.2]	0.13 (-0.33, 0.58)	0.56

TC: total cholesterol; HDL: High-density lipoprotein cholesterol; LDL: low-density lipoprotein cholesterol; TAG: triacylglycerol; N: number of participants

**Table 8:** Lipid parameters expressed in mM in the high total cholesterol group (TC ≥6.0 mmol/L) from baseline to 6 weeks intervention study, in the active (*Lactobacillus plantarum* ECGC 13110402) and placebo treatment groups.

Outcome	Group	N	Baseline Mean (SD)	6 weeks Mean (SD)	Change Mean (SD) [range]	% Change Mean (SD) [range]	Group Difference(*) Mean (95% CI)	P-value
**TC**	**Placebo**	3	7.10 (0.40)	7.40 (0.46)	0.30 (0.78) [-0.2, 1.2]	4.6 (11.5) [-2.7, 17.9]	0	
	**Active**	3	6.20 (0.26)	5.70 (0.53)	-0.50 (0.70) [-1, 0.3]	-7.8 (11.2) [-15.4, 5]	-2.44 (-4.77, -0.11)	**0.045**
**HDL**	**Placebo**	3	1.60 (0.2)	1.67 (0.15)	0.07 (0.06) [0.0, 0.1]	4.5 (3.9) [0.0, 7.1]	0	
	**Active**	3	1.40 (0.2)	1.33 (0.40)	-0.07 (0.21) [-0.3, 0.1]	-6.2 (16.5) [-25.0, 6.3]	-0.06 (-0.53, 0.41)	0.72
**LDL**	**Placebo**	3	4.97 (0.32)	5.13 (0.15)	0.17 (0.46) [-0.1, 0.7]	3.8 (9.9) [-2, 15.2]	0	
	**Active**	3	4.27 (0.64)	3.87 (0.49)	-0.40 (0.70) [-0.9, 0.4]	-8.3 (16.3) [-18.0, 10.5]	-1.21 (-2.64, 0.21)	0.07
**TAG**	**Placebo**	3	1.13 (0.42)	1.37 (0.99)	0.23 (0.58) [-0.1, 0.9]	11.3 (39) [-12.5, 56.3]	0	
	**Active**	3	1.20 (0.56)	1.07 (0.40)	-0.13 (0.38) [-0.4, 0.3]	-3.4 (41.2) [-36.4, 42.9]	-0.38 (-1.82, 1.07)	0.47

TC: total cholesterol; HDL: High-density lipoprotein cholesterol; LDL: low-density lipoprotein cholesterol; TAG: triacylglycerol; N: number of participants

(*) Calculated from ANCOVA analysis, adjusting for baseline value

#### High density lipoprotein cholesterol (HDL-C)

HDL-C increased slightly between baseline and 12 weeks for both placebo and active groups. On adjusting for variable baselines, HDL-C concentrations in the all subject and N-TC groups were 0.06mmol/l (4.5%) and 0.09mmol/l (7.4%) higher in the active group when compared to the placebo. Most of this difference occurred in the 6-12week period for both all subject (P = 0.023, 0.06 Change Mean (SD) [range], 3.4% Change Mean (SD) [range], 0.06 Group Difference Mean (95% Cl) (Table 6) and and N-TC groups (P = 0.33, 0.10 Change Mean (SD) [range], 6.2% Change Mean (SD) [range], 0.09 Group Difference Mean (95% Cl). Age stratification (<50, 50–59, and 60+) revealed statistically significant group differences in the 60+ group (n = 12) with average increases in HDL cholesterol of 0.23mmol/l (14.7%) when compared to the placebo group. Stratification according to baseline TC concentrations and gender revealed no significant treatment effect on HDL-C levels.

#### Low density lipoprotein cholesterol (LDL-C)

LDL-C cholesterol was reduced between baseline and 12 weeks in all the active treatment groups, an effect that was not observed in the placebo group. LDL-C concentrations in the N-TC group were significantly lower by 0.39mmol/l (13.9%) in the active compared to the placebo group (P = 0.03). In the M-TC group, LDL-C showed an average 0.47mmol/l decrease (13.1%), but this did not reach statistical significance. The LDL-C reducing effect appeared to occur consistently for both the 0–6 and 6–12 week periods.

Stratification according to gender, revealed a more pronounced LDL-C reducing effect in female volunteers compared to males however this was not statistically significant (P = 0.06). Active group concentrations were 0.41mmol/l (12.4%) lower for females compared to placebo while a 0.06mmol/l (1.8%, P = 0.06) while an increase was observed for the active male group, compared to placebo (P = 0.83).

Stratification according to volunteer age showed a trend for higher decreases in LDL-C concentrations with increasing age. Only slight changes were observed in baseline adjusted LDL cholesterol concentrations in the <50 years group (0.08mmol/l increase). LDL-C decreases were more pronounced in the 50–59 group (0.49mmol/l) and in the ≥60 years group (0.31mmol/l), corresponding to a 15% and 9.14% decrease respectively in the active group compared to the placebo.

#### Triacylgycerides (TAG)

No statistically significant effects were observed on triacylgyceride concentrations upon the ingestion of either the active or placebo treatments in the all subjects, L-TC, M-TC or H-TC groups. Age stratification using <50 (n = 16), 50–59 (n = 18), and 60+ (n = 12) showed a statistically significant (P = 0.002) triglyceride reduction in the 60+ group of 0.48mmol/l (53.9%) between the placebo and active group during the baseline to 12 week study period. The reduction in triglycerides was more pronounced during the 6–12 week period, with a statistically significant reduction of 0.26mmol/l (32.9%; P = 0.03). A reduction of 0.38mmol/l (31.4%) was observed in the baseline to 6 week period but that was not statistically significant (P = 0.47).

#### Inflammation/ immune biomarkers

No statistically significant changes were noted in IL-6, TNF-α, CRP and IL-10 between baseline and 12 weeks (Table 9), or any of the mid study analysis time points. Stratification into groups according to baseline TC levels, gender or age did not have an impact on any of the parameters analysed here.

**Table 9:** Change in inflammatory biomarkers in all subjects from baseline to 12 weeks.

Outcome	Group	N	Baseline Mean (SD)	12 weeks Mean (SD)	Change Mean (SD) [range]	% Change Mean (SD) [range]	Group Difference (*) Mean (95% CI)	P-value
**IL-6** ^(#)^	**Placebo**	22	54.61 (1.07)	54.52 (1.01)	-0.09 (0.47) [-1.44, 0.82]	-0.2 (0.8) [-2.6, 1.5]	0	
	**Active**	23	54.63 (0.92)	54.68 (0.98)	0.06 (0.26) [-0.69, 0.73]	0.1 (0.5) [-1.3, 1.3]	0.15 (-0.07, 0.37)	0.18
**IL-10** ^(#)^	**Placebo**	22	(25.49)	74.21 (25.5)	-0.01 (0.12) [-0.44, 0.22]	0.0 (0.2) [-0.8, 0.2]	0	
	**Active**	23	(23.08)	66.3 (23.07)	0.04 (0.24) [-0.23, 1.04]	0.1 (0.4) [-0.5, 1.8]	0.05 (-0.07, 0.17)	0.38
**TNF-α** ^(#)^	**Placebo**	22	113.7 (5.24)	113.7 (5.23)	0.00 (0.03) [-0.06, 0.05]	0.0 (0.0) [-0.1, 0.0]	0	
	**Active**	23	112.3 (4.93)	112.4 (4.93)	0.02 (0.08) [-0.07, 0.38]	0.0 (0.1) [-0.1, 0.3]	0.02 (-0.02, 0.06)	0.31
**CRP**	**Placebo**	22	4.0 (7.4)	1.6 (2.0)	-2.4 (6.1) [-26, 1.3]	-11 (67.8) [-94.9, 121]	0	0.57
	**Active**	23	3.1 (4.6)	2.2 (2.6)	-0.9 (2.9) [-13.1, 1.4]	69 (341) [-82, 1604]	0.8 (-0.2, 1.8)	0.11

(*) Calculated from ANCOVA analysis, adjusting for baseline value;

^(#)^ Summary statistics reported in thousands; N: number of participants

#### 16S rDNA next generation sequencing outcomes

**Fig 2:**
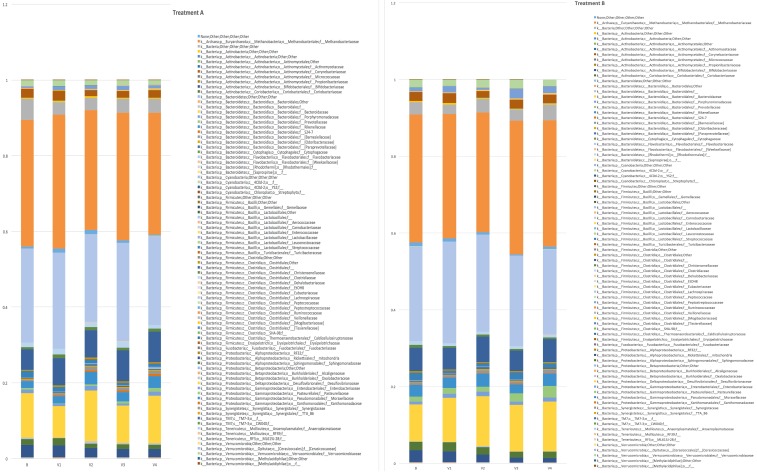
**(A) Histograms representing the relative abundance at family level of the mean between all the samples underwent to the same treatment (A: *Lactobacillus plantarum* ECGC 13110402) at different time point (B = basal, V1 = visit 1, V2 = visit 2, V3 = visit 3, V4 = visit 4). Relative abundances were filtered in order to keep those families that were founded in at least 10% of the subjects at 0.01% of abundance.** Legend reports family names according to Greengenes syntax. (B) Histograms representing the relative abundance at family level of the mean between all the samples underwent to the same treatment (B: placebo) at different time point (B = basal, V1 = visit 1, V2 = visit 2, V3 visit 3, V4 = visit 4). Relative abundances were filtered in order to keep those families that were founded in at least 10% of the subjects at 0.01% of abundance. Legend reports family names according to Greengenes syntax.

A total of 3,225,782 high-quality reads of the V3-V4 region of the 16S rDNA gene were obtained (mean 17,921, SD 1581) and clustered in 41,919 operational taxonomic units (OTUs). With the aim of assessing if the treatment had an impact on the GM composition, relative abundances at family level for each time-point were represented (Fig 2A and 2B, treatment and placebo, respectively). The overall faecal microbiota configuration over time was dominated by 3 families: *Bacteroidaceae*, *Lachnospiraceae* and *Ruminococcaceae* and their relative abundance did not change significantly despite the treatment (p >0.05). Overall, metagenomics data did not show any variation based upon *Lactobacillus plantarum* ECGC 13110402 (A) or placebo (B) or time (baseline or 12 weeks). When *Lactobacillus* population relative abundance in comparison between *Lactobacillus plantarum* ECGC 13110402 and placebo was specifically analysed, no significant differences (*P*>0.05) were observed.

#### Global analysis of urine and serum metabolites

**Fig 3:**
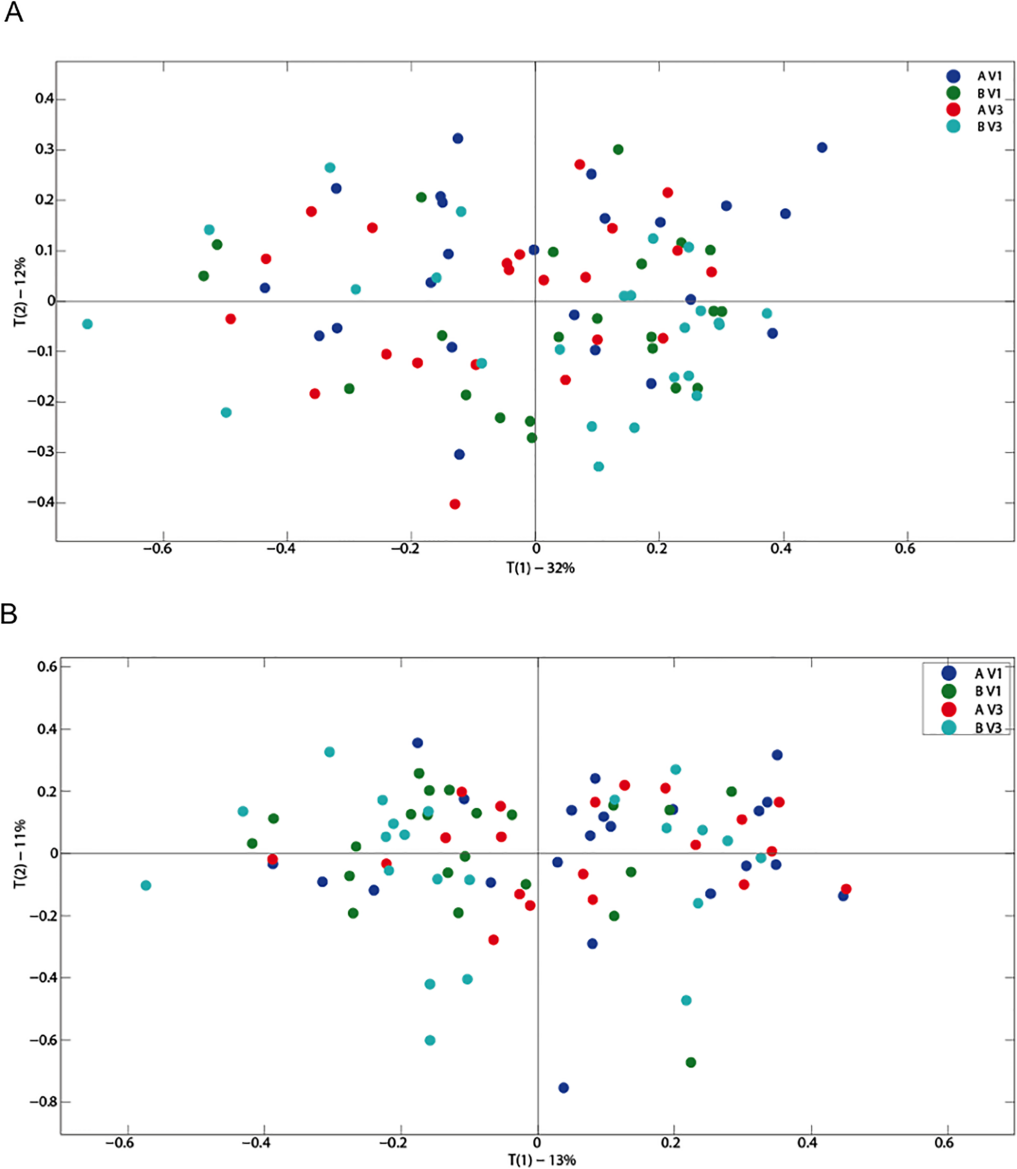
(A) A PCA scores plot for urinary metabolites for all treatments (A: *Lactobacillus plantarum* ECGC 13110402 and B: placebo) and baseline (V1) and 12 weeks (V3) (n = 91); (B) A PCA scores plot for urinary metabolites for all treatments (A: *Lactobacillus plantarum* ECGC 13110402 and B: placebo) (A and B) and baseline (V1) and 12 weeks (V3) (n = 86).

**Fig 4:**
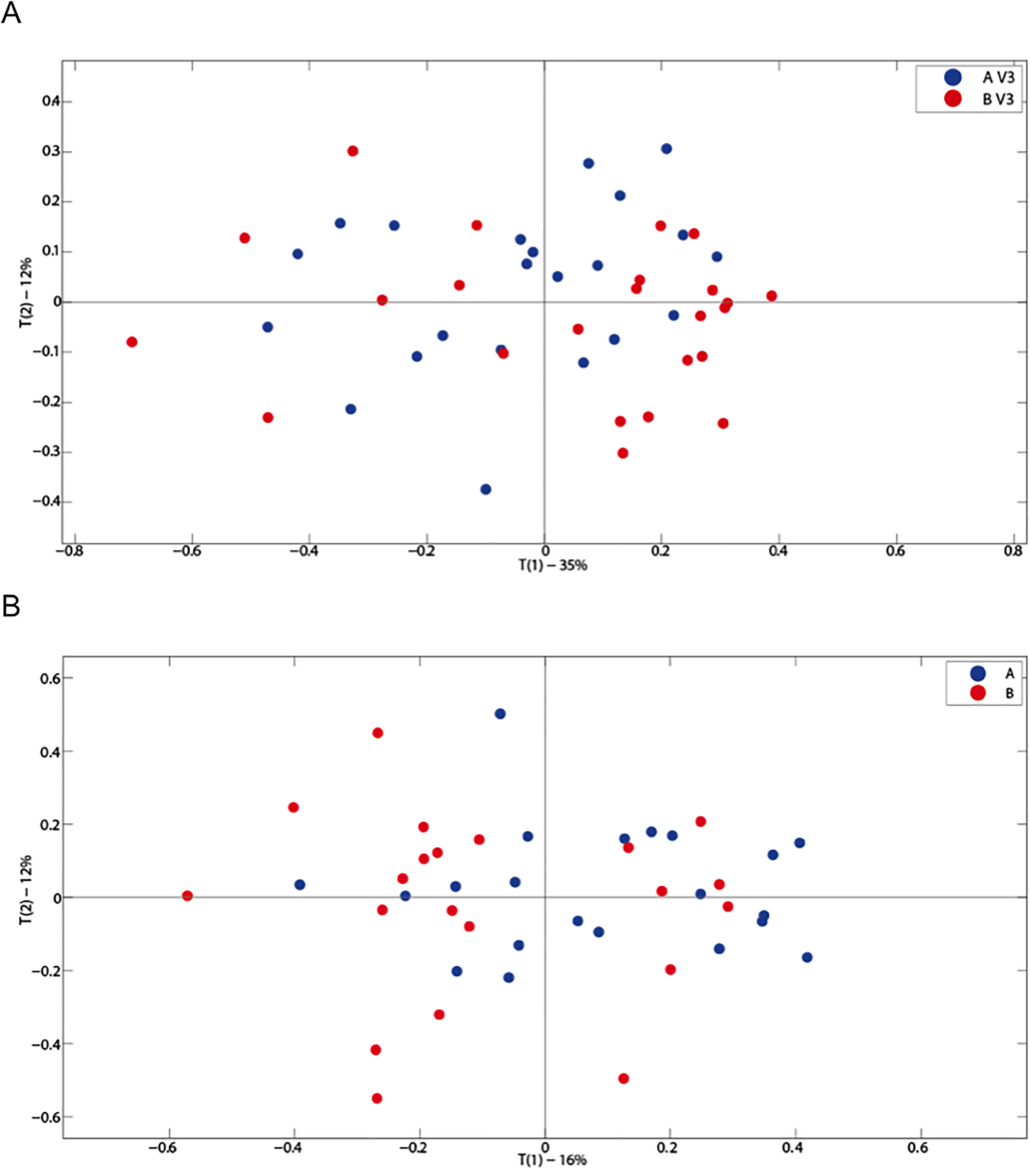
A PCA scores plot for serum metabolites at baseline comparing treatments A and B (A: *Lactobacillus plantarum* ECGC 13110402 and B: placebo) (A); A PCA scores plot for serum metabolites at 12 weeks comparing treatments A and B (A: *Lactobacillus plantarum* ECGC 13110402 and B: placebo) (B).

Primary component analysis of serum and urinary metabolites did not show any variation based upon active or placebo ingestion or time (baseline or 12 weeks). No differences were observed in urinary (Fig 3A and 3B) or serum (Fig 4A and 4B) metabolic profiles when comparing active or placebo at 12 weeks. OPLS analysis did not show any alteration in metabolic profiles in blood and urine by either treatment or placebo use. Individual serum metabolic profiles remained similar between baseline and 12 weeks regardless of treatment.

#### Gastrointestinal symptoms

Volunteers were asked to complete daily gastrointestinal (GI) symptom diaries throughout the duration of the study and to report any adverse effects experienced. GI symptoms for abdominal pain, bloating and flatulence were recorded as none (0), mild (1), moderate (2) or severe (3). Number of daily bowel movements were recorded and stool consistency was scored according to the Bristol stool chart. Table 10 shows the average scores of the self-reported GI symptoms. No significant difference was noted between the active and the placebo groups in any of the parameters scored. None of the study participants reported severe side effects over the 12-week treatment period and no significant differences in stool morphology and frequency were observed between treatment groups.

**Table 10:** Average, self-reported, gastrointestinal (GI) symptom scores for abdominal pain, bloating and flatulence recorded as none (0), mild (1), moderate (2) or severe (3) over the 12-week intervention period for the placebo and *Lactobacillus plantarum* ECGC 13110402) treatment groups. Stool consistency was scored according to the Bristol stool chart.

	Placebo (N = 23)	Active (N = 23)
	Average (±SD)	Average (±SD)
**Bowel movements**	1.28 (0.53)	1.27 (0.51)
**Stool consistency**	3.35 (1.25)	3.55 (0.90)
**Abdominal pain**	0.15 (0.18)	0.32 (0.47)
**Bloating**	0.28 (0.31)	0.35 (0.49)
**Flatulence**	0.68 (0.44)	0.53 (0.48)

## Discussion

The aim of this study was to investigate the cholesterol reducing capacity of *Lactobacillus plantarum* ECGC 13110402 in healthy, normal to mildly hypercholesterolaemic adults. Primary efficacy outcomes included effect on blood lipids, safety and tolerance; secondary outcomes included blood pressure, immune biomarkers, gut microbiota analysis and metabonome changes.

This study used a strain, *Lactobacillus plantarum* ECGC 13110402, identified after *in vitro* screening of 353 microbial strains for mechanisms of action known to influence cholesterol reduction and having high simulated gut survivability. The *in vitro* screening identified *Lactobacillus plantarum* ECGC 13110402 as having high BSH activity, ability to reduce cholesterol, high preferential resistance to gastric, pancreatic, and bile acids and high survivability on freeze drying. Adopting a systematic approach to selecting microbial strains with known mechanisms of action (bile salt hydrolysis) relevant to established disease biomarkers (cholesterol), introduces a baseline rationale to probiotic strain selection, controls the effects of strain variability commonly described in previous studies [7], while increasing the likelihood of reliable outcomes in human studies.

The results of the study demonstrate biologically and/ or statistically significant effects across several CHD risk factors, particularly LDL, HDL, and blood pressure, in normal to mildly hypercholesterolaemic subjects.

One of the primary targets for reducing cardiovascular risk is LDC-C. Study results suggest a consistent pattern for reduction in the active group compared to the placebo across 0–6, 6–12, and 0–12 week periods. Changes in LDL-C varied from 0.24mmol/l (7.2%) in the all subject group to 0.39mmol/l (13.9%) in the N-TC group (P = 0.03). Whilst individual group and subgroup analysis demonstrated variations in statistical significance, there was a consistent trend towards lower LDL values across all active groups. These reductions have potential clinical significance as a 1% reduction in serum cholesterol has been associated with a lessening of artery disease risk by 2–3% [18].

Correlation analyses to examine changes in TC levels between two time points showed a statistically significance (P = 0.04) reduction in TC between baseline and 6 weeks in the active group. This effect was not seen in the placebo group. There was a similar consistent decrease in TC levels when comparing the average active treatment to the placebo. This ranged from 0.12mmol/l (2.3%) in the all subject group to a statistically significant 2.44mmol/l reduction (36.7%, P = 0.045) in the H-TC group. TC reductions of 0.19mmol/l (4.2%) and 0.23mmol/l (4.17%) were seen in the N-TC and M-TC group respectively. The highest impact on LDL-C levels was observed in the H-TC group, however the small population of this subgroup impacts negatively on the strength of this observation. A recent meta-analysis of 30 randomised control trials (1624 participants) on probiotic intake and lipid concentrations by Cho & Kim [7] identified the starting baseline concentration of TC as a significant factor in study outcomes. The higher the baseline TC, the greater the effects observed on TC and LDL levels. Taking these findings into consideration, it can be suggested that individuals with high baseline TC could benefit the most by *Lactobacillus plantarum* ECGC 13110402 treatment, however this should be confirmed in an appropriately powered study in a relevant population.

A recent study performed in 60 hypercholesterolaemic participants investigated the impact of a combination of strains of *Lactobacillus plantarum*. However, this showed variable reductions in TC and LDL-C depending on the baseline cholesterol with only a marginal effect on LDL-C in participants with cholesterol <6.5 mmol/l [19].

Classical epidemiology investigations of atherosclerotic plaque formation, and intervention trials have identified LDL-C as a causative agent in coronary artery disease. As such, reduction of LDL-C is a cornerstone of cardiovascular risk reduction worldwide. Briel et al. [20] in a meta-analysis investigating the association between change in LDL-C and cardiovascular morbidity and mortality showed that a 0.26mmol/l LDL-C decrease reduced the relative risk of coronary heart disease deaths by 7.2% (P = 0.001), coronary heart disease events by 7.1% (P<0.001) and total deaths by 4.4% (P = 0.002). Statins are currently the most effective agents for reducing LDL-C levels. However, of approximately 20 million patients treated with statins, an estimated 10% to 20% are unable to tolerate the intervention, or the higher doses necessary to achieve current LDL-C goals, [21] primarily because of muscle-related side effects. Increasing recognition of the limitations of statins has led to growing interest in non-drug therapies to improve blood cholesterol profiles, particularly when pharmaceutical treatment is considered unsuitable due to elevated cost, safety reasons or just personal preference. Plant stanols and sterols are an increasing option. The European Food Standard Agency (EFSA) reviewed over 80 studies and concluded that for an intake of 1.5–2.4 g/d (3–4 teaspoonfuls) an average reduction in LDL-C of between 7 and 10.5% can be expected. The panel considered that such a reduction is of biological significance in terms of reduced risk of coronary heart disease. The results of this study, using *Lactobacillus plantarum* ECGC 13110402, suggest that a similar reduction to that of stanols and sterols may be achievable [22].

Several cholesterol reducing mechanisms have been suggested for probiotic bacteria to date. *Lactobacillus* strains have been shown to passively bind cholesterol onto their cellular membrane and, as such, remove it from circulation [23,24]. Cholesterol can also be incorporated into the bacterial cell membrane during growth, a mechanism that increases membrane strength and resistance towards lysis [24,25]. Cholesterol can also be converted to coprostanol in the gut by bacterial cholesterol reductases and is then directly excreted in faeces. This decreases the amount of absorbed cholesterol and results in a reduction in the physiological cholesterol pool. Lye *et al* [25, 26] characterised both intracellular and extracellular cholesterol reductases in a range of *Lactobacillus* strains, which could convert cholesterol to coprostanol during *in vitro* fermentation. Another reported mechanism of cholesterol reduction by *Lactobacillus* is through BSH activity which is responsible for bile salt deconjugation in enterohepatic circulation. BSH activity has been previously described for *L*. *plantarum* strains [27]. Bile salt hydrolases (BSHs) catalyse the hydrolysis of the C24-acyl-amide bond of conjugated bile acids and as such remove them from enterohepatic circulation requiring the utilisation of cholesterol in the liver.

Upon ingestion of the active treatment there was evidence of a difference in systolic blood pressure between baseline and 12 weeks. The main reduction in systolic blood pressure occurred in the 6–12 week study period and was 6mmHg (5.1%) lower in the active group compared to placebo (P = 0.003). Similar to high LDL-C, high blood pressure is an established CVD risk factor, with a 10 mm Hg reduction in systolic blood pressure leading to a 13% reduction in all-cause mortality from coronary heart disease, stroke and heart failure [28]. However, as this study was carried out in volunteers with normal blood pressure, the impact of the intervention should be confirmed in hypertension sufferers. Other studies have suggested a role of probiotics in reducing blood pressure. A meta-analysis of nine trials [29] showed that probiotic consumption reduced systolic blood pressure by 3.56mm Hg and diastolic blood pressure by 2.38mm Hg compared with control groups. Possible described mechanisms for the antihypertensive potential of probiotics include the improvement of lipid profiles, insulin resistance, modulation of renin, the bioconversion of bioactive isoflavones and reduction in body weight [26]. *L*. *plantarum* ECGC 13110402 ingestion did not influence body weight here, however the impact on systolic pressure could be attributed to improved lipid profiles, an effect that has been previously described in patients suffering by systolic hypertension [27]. Other mechanisms may have also been involved but were beyond the scope of this study.

The combination of reduction in TC, LDL and blood pressure is a significant advantage over existing approaches as the ability to reduce both LDL and systolic blood pressure may have a multiplicative effect in reducing cardiovascular risk [28–32].

No findings of clinical significance were identified in pro-inflammatory biomarkers, demonstrating safety of the intervention. No adverse GI side effects were noted upon ingestion of *Lactobacillus plantarum* ECGC 13110402 and self-reported parameters relevant to flatulence, abdominal pain, bowel movements and faecal consistency were not significantly different between the active and placebo groups, indicating that the intervention was well tolerated by all volunteers.

Metagenomic analysis of GM composition obtained at baseline and at the end of the intervention study period (12 weeks) did not reveal any significant changes. However, the analytical approach used here examines the *Lactobacillus* genus density and cannot account for inter species changes within the group.

Metabonomic analysis of serum and morning urine samples did not reveal any significant changes in the metabolic profiles between baseline and the end of the intervention (12 weeks). Metabolite concentrations, in blood in particular, are under homeostatic control and as such it may require a higher impact intervention, such as a prebiotic, to affect a measurable change. To this end, we have synthesised a targeted prebiotic galactooligosaccharide using β-galactosidases from *Lactobacillus plantarum* ECGC 13110402 that can selectively stimulate *Lactobacillus* levels *in vitro* in pH controlled faecal culture models and can significantly stimulate cholesterol removal and BSH activity of *Lactobacillus plantarum* ECGC 13110402. This is currently under further testing.

Whilst biologically and statistically significant effects were seen in the current study, it is possible that sample size limited the value of subgroup analysis, particularly in the H-TC group. It is anticipated that further studies with larger numbers would allow a clearer representation of significant effects. Similarly, a longer study duration and/or crossover design may emphasise current findings.

The results of this study demonstrate statistically significant effects across a number of CHD risk factors, particularly LDL, HDL, and blood pressure in normal to mildly hypercholesterolaemic subjects. The results provide early evidence that *Lactobacillus plantarum* ECGC 13110402 has potential as a well tolerated, easy to use, natural cholesterol reducing supplement, as an alternative or supplement to existing treatments. These results, in a non-optimised product, in healthy adults suggest efficacy similar to 1.5–2.4 g plant sterols/stanols per day.

## Conclusions

The study was designed to explore effects across a broad healthy population and was not targeted at a high cholesterol group to demonstrate clinical efficacy. It is believed optimisation of the study protocol, dosage timing, and targeting a higher cholesterol population and/or high blood pressure group and potentially combination with a *Lactobacillus plantarum* targeted prebiotic would further enhance and demonstrate its cholesterol lowering activity of *in vivo*.

## Supporting information

S1 Checklist [Consort-2010-check list-MS]. (PDF)

S2 Checklist [Protocol research committee ethics]. (PDF)

S3 Checklist [Primary and secondary outcomes of the study]. (PDF)
