# Supplementary material for: An in vivo assessment of the cholesterol-lowering efficacy of Lactobacillus plantarum ECGC 13110402 in normal to mildly hypercholesterolaemic adults
Source: PLoS One. 2017 Dec 11;12(12):e0187964. doi: 10.1371/journal.pone.0187964 (PMC5724841; doi:10.1371/journal.pone.0187964)
Supplement: S1 Checklist — (PDF) [file pone.0187964.s001.pdf]

CONSORT 2010 checklist of information to include when reporting a randomised trial*

Section/Topic	Item No	Checklist item	Reported on page No
**Title and abstract**			
	1a	Identification as a parallel, double blind, placebo controlled, randomised trial	2
	1b	Structured summary of trial design, methods, results and conclusions (for specific guidance see CONSORT for abstract)	2
Introduction			
Background and objectives	2a	Specific Background and explanation of rationale	5-8
	2b	Specific objectives and hypotheses	4-5
Methods			
Trial design	3a	Description of the trial design (such as parallel, factorial) including allocation ratio	7
	3b	Important changes to methods after trial commencement with reasons	6
Participants	4a	Eligibility criteria for participants	5
	4b	Setting and locations where the data were collected	6 +24
Interventions	5	The interventions for each group with sufficient details to allow replication, including how and when they were actually administrated	5-6
Outcomes	6a	Completely defined pre-specified primary and secondary outcome measures, including how and when they were assessed	24-33
	6b	Any changes to trial outcomes after their trial commenced with reasons	NA
Sample size	7a	How sample size was determined	6
	7b		NA
Randomisation:			
Sequence generation	8a	Method used to generate the random allocation sequence	6
	8b	Type of randomisation; details of any restriction	6
Allocation concealment	9	Mechanism used to implement the random allocation sequence describing any steps taken to conceal the sequence until interventions were assigned	NA

mechanism			
Implementation	10	Who generated the random allocation sequence, who enrolled participants and who assigned to interventions	6
Blinding	11a	If done who was blinded after assignment to interventions and how	6
	11b	If relevant, description of the similarity of interventions	NA
Statistical methods	12a	Statistical methods used to compare groups for primary and secondary outcomes	25-33
Results			
Participant flow (a diagram is strongly recommended)	13a/13b		5-6, 24-33
Recruitment	14a	Dates defining the periods of recruitment and follow up	6
	14b	Why the trial ended or was stopped	NA
Baseline data	15	A table showing the Demographic and clinical characteristics for each group	24
Numbers analysed	16	For each group number of participants included in each analysis and whether the analysis was by original assigned groups	
Outcomes and estimation	17a	For each primary and secondary outcome, the results for each group and the estimated effect have shown 95% confidence interval.	24
Ancillary analyses	17 b	NA	NA
	18		NA
Harms	19	All important harms or unintend effects in each group	NA
Discussion	20	Trial limitations, addressing sources of potential bias, imprecision, and if relevant multiplicity of analyses	NA
Limitations			
Generalisability	21	Generalisability of the trial findings	NA
Interpretation	22		16-22

**Other information**			
Registration	23	Registration number and name of the trial	1 and 5
Protocol	24	Where the full trial protocol can be accessed if available	5
Funding	25	Sources of funding and other support, role of the funders	20

*We strongly recommend reading this statement in conjunction with the CONSORT 2010 Explanation and Elaboration for important clarifications on all the items. If relevant, we also recommend reading CONSORT extensions for cluster randomised trials, non-inferiority and equivalence trials, non-pharmacological treatments, herbal interventions, and pragmatic trials. Additional extensions are forthcoming: for those and for up to date references relevant to this checklist, see www.consort-statement.org.
