# Supplementary material for: An in vivo assessment of the cholesterol-lowering efficacy of Lactobacillus plantarum ECGC 13110402 in normal to mildly hypercholesterolaemic adults
Source: PLoS One. 2017 Dec 11;12(12):e0187964. doi: 10.1371/journal.pone.0187964 (PMC5724841; doi:10.1371/journal.pone.0187964)
Supplement: S2 Checklist — (PDF) [file pone.0187964.s002.pdf]

School of Chemistry Food and Pharmacy, Department of Food and Nutritional Sciences
Research Ethics Committee

Application Form

SECTION 1: APPLICATION DETAILS

1.1

Project Title: PROBIOCHOL: The cholesterol-lowering efficacy of probiotic *Lactobacillus plantarum* 2830 in hypercholesterolaemic adults.

Date of Submission: December 2014 Proposed start date: January 2015 Proposed End Date: October 2015

1.2

Principal Investigators: Ivan Buttarazzi and Dr. Adele Costabile

Office room number: 4.27 Internal telephone: 0118 378 6218
Email address: a.costabile@rdg.ac.uk Alternative contact telephone:

Other applicants

Name: Glenn Gibson (Staff) Department :Department of Food and Nutritional Sciences Email: g.r.gibson@reading.ac.uk
Internal telephone: 0118 378 8715

1.3

Project Submission Declaration

I confirm that to the best of my knowledge I have made known all information relevant to the Research Ethics Committee and I undertake to inform the Committee of any such information which subsequently becomes available whether before or after the research has begun.

I understand that it is a legal requirement that both staff and students undergo Criminal Records Checks when in a position of trust (i.e. when working with children or vulnerable adults).

I confirm that a list of the names and addresses of the subjects in this project will be compiled and that this, together with a copy of the Consent Form, will be retained within the School for a minimum of five years after the date that the project is completed.

Signed…………………………… (Principal Investigator) Date:…………

…………………………… (Student) Date:…………

…………………………… (Other named investigators) Date:…………

…………………………… (Other named investigators) Date:…………

1.4

University Research Ethics Committee Applications

Projects expected to require review by the University Research Ethics Committee must be reviewed by a member of the School research ethics committee and the Head of School before submission.

Signed…………………………… (Chair of School Committee) Date:……………………

Signed…………………………… (Head of School) Date:……………………

SECTION 2: PROJECT DETAILS

2.1

Lay summary

Please provide a summary of the project in non-specialist terms, which includes a description of the scientific background to the study (existing knowledge), the scientific questions the project will address and a justification of these. Please note that the description must be sufficient for the committee to take a reasonable view on the likely scientific rigour and value of the project.

Coronary heart disease (CHD) is one of the major causes of death and disability in industrialised countries. Results from several epidemiological and clinical studies indicate a positive correlation between elevated total serum cholesterol levels, mainly reflecting the LDL-cholesterol fraction, and risk of CHD. It is thought that a reduction in total plasma cholesterol levels in populations suffering from primary hypercholesterolemia (elevated cholesterol) can lower the incidence of coronary thrombosis.

Currently, therefore there is extensive interest in the management of serum cholesterol and other blood lipids. Diet is viewed as a major influencing factor that can reduce levels. This is largely driven by the expense of drug therapy, the large numbers of individuals affected and unwanted side effects of such treatments. Dietary strategies for prevention of CHD implicate adherence to a low-fat/low-saturated fat diet. Although such diets may present an effective approach, they are difficult to maintain on a long-term basis and efficacy diminishes over time. As such, new approaches towards identification of other dietary means of reducing blood cholesterol levels have been evaluated. These include, among others, the use of probiotics. Probiotics are 'live microbial feed supplements that offer a benefit to health.'

They are marketed as health or functional foods whereby they are ingested for their purported positive advantages in the digestive tract and/or systemic areas like the liver, vagina or bloodstream. They are completely safe for human use. We would like to test whether probiotics can directly degrade cholesterol as well as produce metabolites that interfere with its synthesis in the liver. The effect may also be partially ascribed to an enzymatic deconjugation of bile acids. We therefore propose to investigate the cholesterol reducing capacity of the probiotic *Lactobacillus plantarum* 2830 in hypercholesterolaemic adults, aged 30-65 years.

Study objectives

Primary

- To test, in humans, a probiotic intervention that exerts cholesterol lowering properties in laboratory experiments
- To determine the effect of *Lactobacillus plantarum* 2830 on the faecal microbiota composition of the volunteers
- To investigate the effect of a probiotic on immunity by measuring various inflammatory/immune biomarkers (e.g. cytokines, calprotectin, C-reactive protein)

Secondary

- To conduct an assessment of stool frequency, stool consistency, bloating, flatulence, abdominal pain and mood changes in a human study

2.2

Procedure

Procedure

Please briefly describe what the study will involve for your participants and the procedures and methodology to be undertaken (you may expand this box as required).

This will be a prospective, double-blind, placebo controlled, randomised, single centred cross-over study in 50 (30-65 years old) individuals. The study consist of a 12 week randomised intervention treatment periods with the probiotic and placebo. Volunteers will provide a baseline blood sample at a pre-screening visit, to check for anaemia. Faecal and blood samples will be collected at site visits at the Department of Food and Nutritional Sciences.

Volunteers will be asked to keep a daily volunteer diary on bowel habit and digestive comfort, and mood during each phase of the study. Additionally, during each of the visits the volunteers will be asked to record all foods and drinks consumed within a four-day food diary. Any adverse medical events which occur during the trial (e.g. headache, gut symptoms) should be recorded in a diary along with medication taken. Daily stool habit should be recorded in a diary. All incidence of respiratory infections and colds should also be reported to the principal investigator. Incidence of illness will be monitored to determine if there are fewer adverse events on the active product. In the case that a volunteer wishes to leave the study they will be able to do so at any point without giving a reason and without prejudice. In such incidences volunteers will be renumerated pro-rata.

Study design

A single-centre, prospective, randomized, double-blind, placebo-controlled, parallel-group trial is planned. The sample size (Dell et al. 2002) will be finalised to detect 0.5 Log10 changes with power set at 0.9, and a significance level of 0.05. Subjects will be randomly distributed into two groups: placebo (sucrose and maltodextrin) or treatment with *Lactobacillus plantarum* 2830. Participants will take the product twice a day before breakfast and before the evening meal as a dietary supplement and advised not change their regular diet or physical activity throughout the trial period. Habitual diet will be assessed by pre-validated 5-day food diaries (2 weekend and 3 week days). Each participant will be asked to consume the treatment composed of a *Lactobacillus plantarum* 2830 strain at a concentration of 4 x 10^9 CFU/ day (0.1g) in capsular format (vegetable) with the addition of filling carrier (0.12g; 30% maltodextrin and 5% sucrose), or the placebo control (0.12g; 30% maltodextrin and 5% sucrose). All participants will be advised on product storage conditions prior to use to ensure product consistency throughout the period of the study. The placebo and probiotic will be packaged by NIZO in The Netherlands and only they will have access to product codes.

The study will consist of two phases: a treatment (probiotic or placebo for 12 weeks) and a wash-out period (4 weeks). The study will include a baseline visit at selection and visits at midpoint and endpoint of the treatment periods (Figure 1).

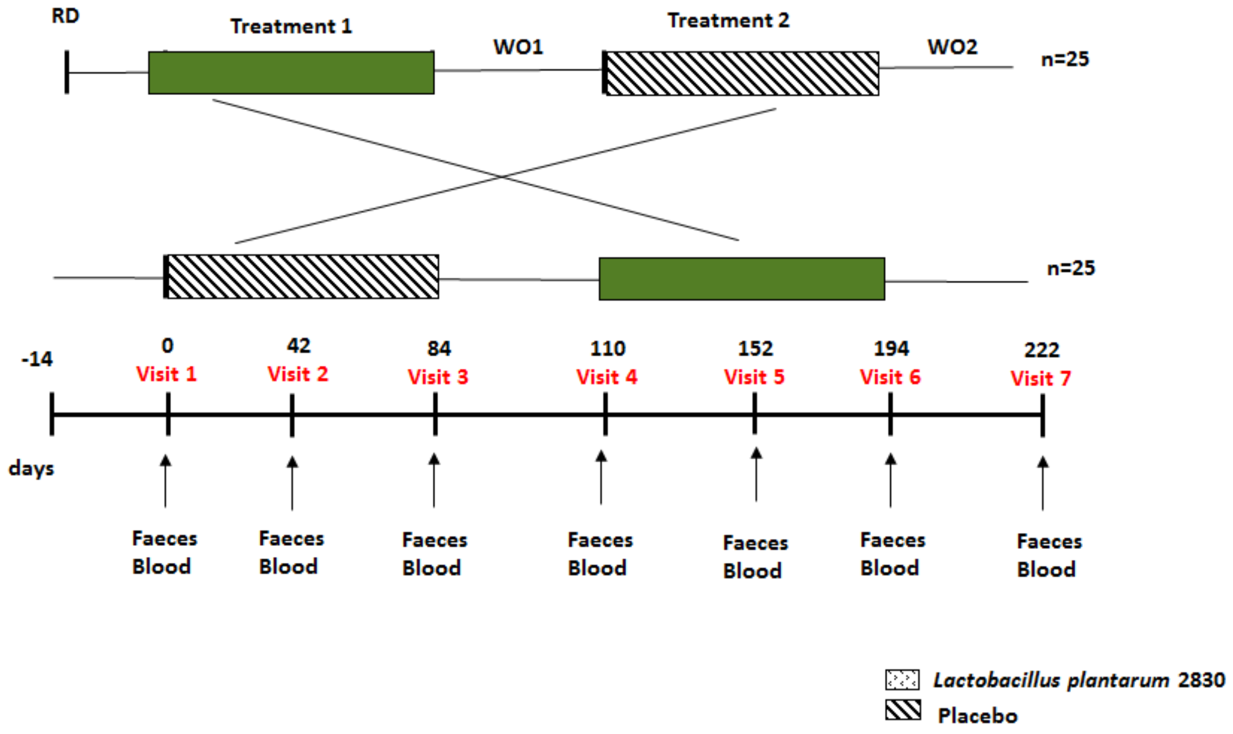

Figure 1- Study design

**Trial Day**	*Stage of Study*	***Treatment***
**-2 weeks**	**Pre trial meeting**	• Briefing about the study<br>• Distribution of trial information sheets and preliminary discussions with volunteers<br>• Provide blood sample for screening<br>• Volunteer diaries (diaries for bowel habit and mood questionnaire
0	**Baseline (Visit 1)**	• Provide baseline faecal sample and a blood sample (50ml; 5 dessert spoons) for baseline measurements<br>• Distribution of test capsules for the 1st 12w treatment<br>Volunteer diaries (diaries for bowel habit and mood questionnaire) will be distributed for the 1st 84 day treatment
**12 weeks**	**1st Treatment period (Visits 2 and 3)**	• Consume one capsule for every day of each 12w treatment period<br>• During the treatment (probiotic or placbo), complete dietary, bowel habit and mood questionnaires and record any concomitant medication or adverse events during the trial<br>• Provide faecal sample and blood sample after 6w and 12w (Visits 2 and 3)
		• Continue to refrain from consuming probiotic and

**4 weeks**	**Washout period (Visit 4)**	prebiotic supplements.<br>• Do not take any product capsules<br>• Provide faecal sample and blood sample<br>• Distribution of test capsules for the 2nd 12w treatment
**12 weeks**	**2nd Treatment period (Visits 5 and 6)**	• Consume one capsule for every day of each 12w treatment period<br>• During the treatment (probiotic or placebo), complete dietary, bowel habit and mood questionnaires and record any concomitant medication or adverse events during the trial<br>• Provide faecal sample and blood sample after 6w and 12w (Visits 5 and 6)
**4 weeks**	**Washout period (Visit 7)**	• Continue to refrain from consuming probiotic and prebiotic supplements.<br>• Do not take any product capsules<br>• Provide faecal sample and blood sample

END OF THE STUDY

Those working with blood and faeces will be up to date with Hepatitis A and B vaccinations.

- *Bacterial enumeration*

  Freshly voided faecal samples will be diluted 1 in 10 (w/w) with anaerobic phosphate buffer and mixed in a stomacher for 2 min. Changes in faecal bacterial populations will be assessed through the use of a culture independent procedure that assesses molecular changes in the microbiota (metagenomics).

- *Short chain fatty acids (SCFA)*

  Samples of the faecal slurry [1 in 10 (w/w) dilution of faeces] will be taken for determination of SCFA (end products of bacterial metabolism) by gas chromatography.

  All investigators coming into contact with faecal matter will have completed a Hepatitis A vaccination course. Any individuals coming into contact with faecal samples will abide by good general laboratory procedures.

- *Inflammatory/immune biomarkers*

  A blood sample to check for anaemia and baseline measurements will be taken during screening (50ml – 5 dessert spoons) (Visit 1). 5 subsequent blood samples will be taken during the trial. Volunteers will be asked to provide a venous, fasting (fasted for 10 hours), blood sample. Blood will be collected by venepuncture by a trained phlebotomist.

  Blood will be extracted into sodium heparin tubes (kept on ice), diluted and centrifuged to gain plasma (for cytokine analysis).

  Various inflammatory/immune biomarkers (e.g. cytokines calprotectin (from dry faeces), and C reactive protein) will be measured using either flow cytometry or ELISA kits. Lipid profile including measures of total, LDL and HDL cholesterol, triglycerides.

  Plasma and serum samples will be labelled as a biohazard and stored at -80°C.

  The Department of Food and Nutritional Sciences is fully licensed under the Human Tissue Act 2004 and as such will adhere to the guidelines necessary for the storage of faeces. A detailed log will be kept to record when the sample was taken, the place of storage, when analysis was conducted on the sample and how and when the sample was disposed of.

Early withdrawal

- Withdrawal Criteria:

Volunteers will be informed in the consent form and also in the volunteer information sheet that they have the right to withdraw from the study at any time without giving a reason and without prejudice. In addition they may be withdrawn at the Investigator's discretion at any time.

- Volunteers may be withdrawn by the Investigator due to:

An adverse event for which the Investigator does not consider continuous study participation safe.
Recurrent illness.
Poor tolerance.
Poor adherence.

- Data to be collected following withdrawal from the study

If possible, the assessments that should be performed at final visit will be performed at the time of withdrawal, while the volunteer is still on treatment. The study termination form should also be completed.

Description of the test product

The product used in this study will be *Lactobacillus plantarum* 2830 combined with sucrose (5%) and maltodextrin (30%) (carriers; powdered carbohydrates). The placebo will be identical to the test capsules but will not contain *Lactobacillus plantarum* 2830. The products will be provided as a powder in capsules, therefore can be kept at room temperature, and also can be taken abroad. *Lactobacillus plantarum* 2830 has been fed to human subjects in trials carried out in Holland. We have data confirming its status as 'safe' for probiotic use that can be supplied on request. It is a harmless, food grade, microorganism.

Serving: ***2 servings per day (2 capsule)***

*Instruction of use:*Take one capsule with a glass of water in the morning with breakfast and one capsule at dinner time. Do not open the capsule.

Visit schedule and assessments

Screening/Pre-Trial meeting (Baseline visit)

Informed consent will be obtained prior to the performance of any study related activities/assessments. The inclusion/exclusion criteria will be reviewed for volunteer eligibility. Here, volunteers will undergo the following evaluations:

- Demography
- Past/current medical conditions
- Weight/ Height
- Concomitant medication
- Smoking history
- Blood sample collection

Note: Blood sample collections can be arranged for another day should volunteer prefer.

In addition, volunteers deemed eligible and who want to participate will be given food and drink diary and instructed on how to complete the diary.

The volunteer will either be excluded or, if fully meeting the inclusion criteria and willing to take part in the study, will be randomised and enrolled. On Day 1, the study volunteer will be given the correct number of capsules for the first treatment period. Food and drink diaries will be collected and diary cards will be issued and the volunteer will be instructed on how to complete these diaries.

Volunteers will keep a daily diary noting the number of bowel movements and the average consistency of the stools using the Bristol stool chart (hard, solid, loose or watery), as well as the occurrence of abdominal discomfort, flatulence or bloating and mood changes. Furthermore the diary will include an area for noting any illness experienced including colds and respiratory infections.

Visit 1: Initiation of the study
Volunteers meeting the inclusion/exclusion criteria will be given the correct number of capsules for the first 12 weeks of the treatment. Food and drink diaries will be collected and daily diary cards will be delivered and the volunteer will be instructed on how to complete the diary cards. Volunteers will provide blood (50ml; 5 dessertspoons) and faecal samples. Concomitant medications will be checked and the volunteer's weight recorded.

Blood sample collection

Volunteers will be asked to provide a venous, fasting (having fasted for 10 hours, drinking only water – but no water 1hr before arrival) blood sample (50ml; 5 dessertspoons) at each visit. Blood samples will be collected by an experienced and trained phlebotomist in the Hugh Sinclair Unit of Human Nutrition (Department of Food and Nutritional Sciences). Samples will be processed immediately in order to isolate PBMCs and conduct immune parameter analyses.

Faecal sample collections

Volunteers will be asked to provide a fresh faecal sample at each visit. The volunteers will be provided with an appropriate vessel for the faecal sample collection at the University. If unable to do so, they will be given the option to come in on preceding days until production is possible. No new treatment will be issued until stool has been provided. Samples will be processed immediately for in preparation for microbial and faecal calprotectin analyses.

Visits 2 and 3: After randomisation or before if volunteer is withdrawn
At Visits **2- 3**, volunteers will provide blood and faecal samples. Adverse Events will be addressed, concomitant medications will be checked and the diary card will be reviewed with the volunteer. New diary cards and the correct number of capsules for the 12 weeks of the treatment will be given.

In the event of withdrawal, all evaluations required will be performed as per the end of the study, provided the subject is willing.

Visit 4:
At Visit 4, volunteers will provide blood (50ml; 5 dessertspoons) and a faecal sample. Adverse Events will be addressed, concomitant medications will be checked, weight will be recorded and the diary card will be reviewed with the volunteer. New diary cards will be distributed and volunteers will not be supplied with feeding capsules at this visit as they will enter 4 weeks of washout period. All unused products will be collected at this visit.

In the event of withdrawal, all evaluations required will be performed as at the end of the study, provided the subject is willing.

Visits 5 and 6: 12 weeks after randomisation or before if volunteer is withdrawn

At Visits 5 and 6, volunteers will provide blood (50ml; 5 dessertspoons), and faecal samples. Adverse Events will be addressed, concomitant medications will be checked, weight will be recorded and the diary card will be reviewed with the volunteer. New diary cards will be distributed and volunteers will be supplied with capsules for the 12 weeks of the second treatment – different from the product they received for the first 12 weeks.
In the event of withdrawal, all evaluations required will be performed as at the end of the study, provided the subject is willing. Visit 6 finalises the study.

Statistical analysis:

Faeces:
Bacterial numbers will be compared after transformation to log counts. This will be done using a repeat measure ANOVA test analysing repeat measures over time using the factors: Treatment group 0 (placebo) or 1 (treatment).

Blood immune and lipid factors:
Each parameter will be analysed separately using summary statistics, whereby a profile plot will be taken with mean values, comparing treatment and placebo on the same plot. The area under the curve will be used to statistically analyse differences in treatment and placebo values.

Volunteer diaries:
We will use a χ^2 test which compares two factors to see if they are independent from each other (in this case the placebo and treatment values): of stool frequency, consistency, abdominal pain and intestinal bloating.

(Note: All questionnaires or interviews should be appended to this application)

2.3

Where will the project take place?

The project will take place within the Department of Food and Nutritional Sciences building, within the Hugh Sinclair Unit (Jan Luff has been contacted).

If the project is to take place in Hugh Sinclair Unit of Human Nutrition, please confirm that you have informed Ms Jan Luff (j.e.luff@reading.ac.uk). Yes, Jan Luff has been informed

2.4

Funding

Is the research supported by funding from a research council or other *external* sources (e.g. charities, business)? Yes

If Yes, please give details:

Stephen OHara
Chief Executive
OptiBiotix Health Ltd
Tel: Mobile: ++ 44 (0) 7887 874392
Email: Spohara@optibiotix.com

Optibiotix is funding, and providing the test products associated with, this study

Please note that *all* projects, (except those considered as low risk, questionnaire-based studies which require Head of School approval) require approval from the University Research Ethics Committee.

2.5

Ethical Issues

Could this research lead to any risk of harm or distress to the participants? Please explain why this is necessary and how any risk will be managed.

Lactobacillus plantarum 2830 is a harmless probiotic. It has been rigorously tested for both safety and efficacy and is a commercially available foodstuff. As such, participation in this study does not pose any significant risk. Please refer to Appendix J for further information.

(*this box may be expanded as required*)

2.7

Payment

Will you be paying your participants for their involvement in the study? Yes

If yes, please specify and justify the amount paid

Time	Task	Payment	Total Payment
2 hours	Assessment and instruction	£ 8.50 / h	£ 17
6 hours	Sample collection	£ 7.00 / h	£ 42
13 hours	Record keeping	£ 7.00 / h	£ 91
**Total 21 hours**			**£ 150**

Volunteers will be paid £150 for completion of the trial. Those who withdraw will have their payment pro-rated according to time spent. Reserve volunteers will be paid for assessment and instruction if they are not required to participate in the treatment phases of the trial.

Note: excessive payment may be considered coercive and therefore unethical. Travel expenses will not be provided.

2.8

Data protection and confidentiality

What steps will be taken to ensure participant confidentiality? How will the data be stored?

Confidentiality will be maintained by allocating volunteers an identification code, which will be used to identify all samples and data obtained. Volunteer's names will not be used in any reports or publications. All data generated from the study will be held securely within a password protected file, only the study investigators will have access to this. A record of the names of the volunteers will not be held on the same file. Information matching volunteer names with identification codes will be kept by a departmental secretary in a locked filing cabinet, the investigators will only use identification codes. The only time data will be matched with volunteer names is for those volunteers that request to have their personal results discussed with them. A request for individual results to be discussed will include a review of all sample results for the individual volunteer. A list of the names and addresses of the subjects in this project will be compiled, this, together with a copy of the Consent Form, will be retained within the Department for five years after the date that the project is completed.

2.9 **Consent**

Please describe the process by which participants will be informed about the nature of the study and the process by which you will obtain consent

A letter will be sent to the Sensory Dimensions Group who run various trials and have a large number of hypercholesterolemic individuals registered on their database. Sensory Dimensions Group will then make contact with individuals matching the correct age profile and give them information sheets. Potential volunteers may then contact the study investigator if they are interested in participating, or if they would like any further information or to discuss any concerns. A meeting will be arranged to talk to the participants before the commencement of the trial. Participants are free to withdraw from the trial at any time without giving reason. Those who wish to participate in the study will attend a pre-trial assessment, in which informed consent will be obtained. Consent will be obtained after volunteers are asked questions about inclusion and exclusion criteria. Volunteers are free to ask the investigator questions at any point during / following this process.

Please note that a copy of consent forms and information letters for all participants must be appended to this application.

2.10

Genotyping

Are you intending to genotype the participants? Which genotypes will be determined?

No

Please note that a copy of all information sheets on the implications of determining the specific genotype(s) to be undertaken must be appended to this application.

SECTION 3: PARTICIPANT DETAILS
3.1<br>**Sample Size**<br>How many participants do you plan to recruit? Please provide a suitable power calculation demonstrating how the sample size has been arrived at or a suitable justification explaining why this is not possible/appropriate for the study. By the use of a statistical power calculation it can be observed that at significance level of 5% (one-sided) will be that, a log change of 0.31 can be detected at a power of 90% with 50 volunteers. (This calculation is based on the assumption that the within patient standard deviation is 0.42, which was as observed by Tuohy *et al.,* 2001). Therefore 50 hypercholesterolaemic volunteers (30-65 years old) will be recruited for participation in this study. We will over recruit by 10% to cover for drop-outs.<br>35-60 year old individuals will be screened according the inclusion and exclusion criteria. From this screening we will select individuals to enrol in the trial.
3.2<br>Will the research involve children or vulnerable adults (e.g. adults with mental health problems or neurological conditions)? No<br><br>If yes, how will you ensure these participants fully understand the study and the nature of their involvement in it and freely consent to participate?<br><br>(Please append letters and, if relevant, consent forms, for parents, guardians or carers). Please note: information letters must be supplied for all participants wherever possible, including children. Written consent should be obtained from children wherever possible in addition to that required from parents.
3.3<br>Will your research involve children under the age of 18 years? No<br>Will your research involve children under the age of 5 years? No
3.4<br>Will your research involve NHS patients, Clients of Social Services or will GP or NHS databases be used for recruitment purposes? No<br><br>Please note that if your research involves NHS patients or Clients of Social Services your application will have to be reviewed by the University Research Ethics Committee and by an NHS research ethics committee.
3.5<br>**Recruitment**<br>Please describe the recruitment process and append all advertising and letters of recruitment.<br>Sensory Dimensions Group will be contracted to do a part of the recruitment as they have a large number of hypercholesterolemic individuals registered on their database. Sensory Dimensions Group will make contact with these individuals and give them information sheets. Volunteers could then contact the study investigator if they are interested in participating, or if they would like any further information or to discuss any concerns. Potential participants will have a minimum of two weeks to decide whether they want to be involved in the study. A meeting will be arranged to talk to the participants before the commencement of the trial prior to informed consent. Participants are free to withdraw from the trial at any time. Advertisements may also be emailed, put in newspapers and on walls in and around the University as well as in care homes (Appendix H).

Important Notes

1. The Principal Investigator must complete the Checklist in Appendix A to ensure that all the relevant steps and have been taken and all the appropriate documentation has been appended.

2. If you expect that your application will need to be reviewed by the University Research Ethics Committee you must also complete the Form in Appendix B.

3. For template consent forms, please see Appendices C.

4. For examples of information letters, see Appendices D.

5. For participant information sheet, see Appendix E.

6. For pre-study questionnaire, see Appendix F.

7. For volunteer diaries, see Appendix G.

8. For advertisement, see Appendix H.

9. For sensory dimensions recruitment letter, see Appendix I

10. Safety assessment data for the probiotic, see Appendix J (letter from NIZO and genomic data confirming the lack of safety regards)

Appendix A: Application checklist

This must be completed by an academic staff member (e.g. supervisor)

Please tick to confirm that the following information has been included and is correct. Indicate (N/A) if not applicable:

Information Sheet

Is on headed notepaper	☐	
Includes Investigator's name and email / telephone number	☐	
Includes Supervisor's name and email / telephone number	☐	
Statement that participation is voluntary	☐	
Statement that participants are free to withdraw their co-operation	☐	
Reference to the ethical process	☐	
Reference to Disclosure	☐	N/A ☐
Reference to confidentiality, storage and disposal of personal information collected	☐	

Consent form(s) ☐

Other relevant material

Questionnaires	☐	N/A ☐
Advertisement/leaflets	☐	N/A ☐
Letters	☐	N/A ☐
Other (please specify)	☐	N/A ☐

Expected duration of the project (months) WO2

Name (print) ……………………………………… **Signature** ………………………………

Appendix B

University of Reading

Project Submission Form

ANNEX B

Note All sections of this form should be completed. Please continue on separate sheets if necessary.

Principal Investigators: Ivan Buttarazzi and Adele Costabile

School: Food and Nutritional Sciences
Email: a.costabile@ reading.ac.uk

Title of Project:
The cholesterol-lowering efficacy of probiotic *Lactobacillus plantarum* 2830 in hypercholesterolaemic adults.

Proposed starting date: January 2015

Brief description of Project:

To investigate the effect of the probiotic *Lactobacillus plantarum* 2830 on the faecal microbiota, blood lipids and immunity in hypercholesterolemic individuals. This will be a prospective, double-blind, placebo controlled, randomised, single-centred cross-over study in individuals aged 30-65 years old. The study will consist of a 8 month randomised double-blind crossover treatment period with probiotic and placebo.

I confirm that to the best of my knowledge I have made known all information relevant to the Research Ethics Committee and I undertake to inform the Committee of any such information which subsequently becomes available whether before or after the research has begun.

I confirm that if this project is an interventional study, a list of names and contact details of the subjects in this project will be compiled and that this, together with a copy of the Consent Form, will be retained within the School for a minimum of five years after the date that the project is completed.

Signed:
………………………………………………………..... Date: ……………………………………
(Investigator)
………………………………………………………..... Date: ……………………………………
(Head of School or
authorised Head of Department)
………………………………………………………..... Date: ……………………………………
(Student -where applicable)

Version – September 2010

Checklist

1. This form is signed by my Head of School (or authorised Head of Department) ☐

2. The Consent form includes a statement to the effect that the application has been reviewed by the University Research Ethics Committee and has been given a favourable ethical opinion for conduct ☐

3. I have made, and explained within this application, arrangements for any confidential material generated by the research to be stored securely within the University and, where appropriate, subsequently disposed of securely. ☐

4. I have made arrangements for expenses to be paid to participants in the research, if any, OR, if not, I have explained why not. ☐

5. EITHER
(a) The proposed research does not involve the taking of blood ☐

samples;
OR
(b) For anyone whose proximity to the blood samples brings a risk of Hepatitis B, documentary evidence of protection prior to the risk of exposure will be retained by the Head of School or authorized Head of Department. ☐
Signed:
………………………………………... Date……………………
(Head of School or
authorised Head of Department)

6. EITHER
(a) The proposed research does not involve the storage of human tissue, as defined by the Human Tissue Act 2004; ☐

OR
(b) I have explained within the application how the requirements of the Human Tissue Act 2004 will be met. ☐

7. EITHER
(a) The proposed research will not generate any information about the health of participants; ☐

OR
(b) In the circumstance that any test reveals an abnormal result, I will inform the participant and, with the participant's consent, also inform their GP, providing a copy of those results to each and identifying by name and date of birth; ☐

OR
(c) I have explained within the application why (b) above is not appropriate. ☐

8. EITHER
(a) the proposed research does not involve children under the age of 5; ☐

OR
(b) My Head of School (or authorised Head of Department) has given details of the proposed research to the University's insurance officer, and the research will not proceed until I have confirmation that insurance cover is in place. ☐

Signed:
……………………………………….. Date………………..…

(Head of School or
authorised Head of Department)
This form and further relevant information (see Sections 5 (b)-(e) of the Notes for Guidance) should be returned to:
Mr Nathan Helsby
Planning Support Office
Room 321
Whiteknights House
Tel: 0118 378 6972
Email: n.e.helsby@reading.ac.uk
- both electronically and in hard copy
You will be notified of the Committee's decision as quickly as possible, and you should not proceed with the project until then.

Department of Food and Nutritional Sciences

Adele Costabile: a.costabile@pgr.reading.ac.uk
Whiteknights
PO Box 266, Reading RG6 6AP, UK
phone 0118 378 6218
fax +44 (0)118 931 0080

University of Reading

Appendix C

Research Ethics Committee

Consent Form

ANNEX C

1. I have read and had explained to me by ……………………………………………..…

the accompanying Information Sheet relating to the project on:

The cholesterol-lowering efficacy of probiotic *Lactobacillus plantarum* 2830 in hypercholesterolaemic adults.

-

2. I have had explained to me the purposes of the project and what will be required of me, and any questions I have had have been answered to my satisfaction. I agree to the arrangements described in the Information Sheet in so far as they relate to my participation.

3. I understand that participation is entirely voluntary and that I have the right to withdraw from the project any time, and that this will be without detriment.

4. I authorise the Investigator to consult my General Practitioner.

5. This application has been reviewed by the University Research Ethics Committee and has been given a favourable ethical opinion for conduct.

6. I have received a copy of this Consent Form and of the accompanying Information Sheet.

7. I have read the Information Sheet and been told the reasons why a blood sample is required. I consent to:
   (1) a single blood sample being taken at screening
   (2) a series of 7 blood samples being taken

8. I have had explained to me that consent for my contact details and personal information to be added to the Nutrition Unit Volunteer Database is entirely voluntary. Accordingly I consent as indicated below:

yes □ no

I consent to my contact details being stored on the Nutrition Unit Volunteer Database.

I consent to my screening information (including date of birth, height, weight, blood pressure, smoking status, long-term use of — yes □ no

University of Reading

Department of Food and Nutritional Sciences

Adele Costabile: a.costabile@pgr.reading.ac.uk
Whiteknights
PO Box 266, Reading RG6 6AP, UK
phone 0118 378 6218
fax +44 (0)118 931 0080

medication, and blood test results such as level of cholesterol, triglycerides, glucose, liver enzymes, and blood cell count) being stored on the Nutrition Unit Volunteer Database.

Name: ……………………………………………………………………………………

Date of birth: …………………………………………………………………………

Signed: …………………………………………..………………………………

Date: ……………………………………………………..………………………

Witnessed by: ………………………………………………………………………….

Date: ……………………………………………………………………………………

Signed: ……………………………………………………………

Please provide details of your GP below:

Name of GP……………………………………………………………………………

Address:……………………………………………………………………………….
………………………………………………………………………………………
………………………………………………………………………………………
………………………………………………………………………………………

Telephone number:……………………………………………………………………

Department of Food and Nutritional Sciences

Adele Costabile: a.costabile@pgr.reading.ac.uk
Whiteknights
PO Box 266, Reading RG6 6AP, UK
phone 0118 378 6218
fax +44 (0)118 931 0080

Name: ………………………………………………………………………………………
Address: ………………………………………………………………………………
…………………………………………………………………………………………
…………………………………………………………………………………………
…………………………………………………………………………………………
Date of birth: ……………………………………………………………………..……
Signed: ………………………………………..…………………………………….
Date: …………………………………………………………...………………………

I have received a copy of this (please tick) ☐
I would like to receive feedback after the study is completed (please tick if yes) ☐

Department of Food and Nutritional Sciences

Adele Costabile: a.costabile@pgr.reading.ac.uk
Whiteknights
PO Box 266, Reading RG6 6AP, UK
phone 0118 378 6218
fax +44 (0)118 931 0080

Appendix D

GP letter

Dear *Name of GP*,

Name of Patient (and date of birth) has expressed an interest in participating in a dietary intervention trial at The University of Reading. The trial will investigate the effect of a probiotic *Lactobacillus plantarum* 2830 on cholesterol levels in hypercholesterolaemic adults.

This will be a prospective, double blind, placebo controlled, randomised, single centred crossover study in individuals aged 30-65 years old. The study will consist of a 8 month randomised double blind crossover treatment period with probiotic and placebo. The subject information sheet has been enclosed for your information.

The product used in this study will be *Lactobacillus plantarum* 2830 (0.1g) served in capsules, twice daily. The placebo will be sucrose and maltodextrin (0.12g) served in capsules, twice daily. *Lactobacillus plantarum* 2830, sucrose and maltodextrin are completely safe. As such the trial is not considered of risk to the volunteers.

This human study has been subject to ethical review, according to the procedures specified by the University Research Ethics Committee, and has been given a favourable ethical opinion for conduct.

The trial will commence on (*Trial start date)*

If you have any questions about the trial please do not hesitate to contact me.

We appreciate your help.

Yours sincerely,

Ivan Buttarazzi
Adele Costabile

Department of Food and Nutritional Sciences
Adele Costabile: a.costabile @.reading.ac.uk
Whiteknights
PO Box 266, Reading RG6 6AP, UK
phone 0118 378 6218
fax +44 (0)118 931 0080

Appendix E

PARTICIPANT INFORMATION SHEET

The cholesterol-lowering efficacy of probiotic *Lactobacillus plantarum* 2830 in hypercholesterolaemic adults.

You are being invited to take part in a research study. Before you decide if you want to take part it is important that you understand what is involved. Please read the following information and discuss with others if you wish. Please ask us if there is anything you do not understand or if you would like any additional information. Take time to decide whether or not you wish to take part.

Aim
The aim of this study is to evaluate the effects of beneficial bacteria (*Lactobacillus plantarum 2830*; a probiotic) on intestinal bacteria, cholesterol and other blood lipids and on immunity in hypercholesterolaemic individuals aged 30-65 years. About 50 volunteers will take part in this study. In addition, a further 5 volunteers will be recruited as reserves. Equal numbers of men and women will be included in the study cohort.

Before you decide whether to take part in the study, please read the following information carefully. If you want to know anything about the study, which is not written here, please ask the investigator.

What are probiotics?

- Probiotics are bacteria that live in the intestine and bring about health benefits
- They are used in foods to increase gut numbers
- They have been found to improve the intestinal bacterial balance of the general population
- They are safe for human consumption
- They have been consumed by humans for hundreds of years

Proposed benefits of high numbers of probiotics
- Reduce the number / activities of disease causing bacteria

University of Reading

Department of Food and Nutritional Sciences
Adele Costabile: a.costabile @.reading.ac.uk
Whiteknights
PO Box 266, Reading RG6 6AP, UK
phone 0118 378 6218
fax +44 (0)118 931 0080

- Improve immune response
- Reduce risk of gastrointestinal illness such as travellers' diarrhoea, irritable bowel syndrome, infections
- Increase absorption of minerals
- Lower blood cholesterol

Why is this study being carried out?
There is an increasing shift towards use of diet to control blood cholesterol and other health parameters. Probiotics are safe interventions that could help.

Inclusion criteria/exclusion criteria
Inclusion criteria- If the following applies to you, you will be considered for participation in the trial:

- Aged between 30 and 65 years of age.
- Willing to participate in the entire study (signed informed consent required).
- Subjects will be eligible for the study if male or female (non pregnant), aged 30– 65 years, with a Total Cholesterol (TC) between 200 and 300 mg/dl (5.16 and 7.64 mmol/L); and Low Density Lipoprotein cholesterol (LDL-C) values between 130 and 190 mg/dl (3.35 and 4.9 mmol/l).

Exclusion criteria - If the following applies to you, you will be unable to participate in the trial:

- History or evidence of intestinal disease; such as tumour, irritable bowel syndrome, *etc*., within the previous 5 years.
- Received antibiotics in the previous six months
- Not willing to cease the consumption of probiotic or prebiotic preparations for the duration of the study
- Former participation in another study involving prebiotic or probiotic preparations within the previous 2-weeks, or intention to use such products during the course of the study (please note sensory evaluations may be still permitted after discussion with the Investigator)
- History of malignancy within the previous 5 years (with exception of well-treated basal cell carcinoma or *in situ* cervical carcinoma).Smoker
- Lactose intolerant
- Allergic to gluten
- Currently prescribed immunosuppressive drugs. Participants will be required to withdraw should they begin taking any of the ineligible medication.

Department of Food and Nutritional Sciences
Adele Costabile: a.costabile @.reading.ac.uk
Whiteknights
PO Box 266, Reading RG6 6AP, UK
phone 0118 378 6218
fax +44 (0)118 931 0080

- *Intention* to use *regularly* other medication which affects gastrointestinal motility.
- History of alcohol or drug misuse.
- Suffer from any major conditions involving the following:
- Head, Ears, Eyes, Nose and Throat
- Dermatological/Connective tissue
- Neurological
- Lymphatic
- Urogenital/Rectal
- Abdominal
- Respiratory
-

A previous cardiovascular event within the last 6 months, presence of secondary dyslipemias related to thyroid dysfunction, used any drug affecting lipid metabolism in previous 3 months, a history of alcohol abuse.

What will I be asked to do?

- All participants will be asked to fill out a health screening questionnaire and inclusion/exclusion criteria will be reviewed for volunteer eligibility.
- Informed consent from yourself will be required
- On giving consent and passing initial screening, participants' height and weight will be measured and they will be required to give a blood sample to screen for anaemia and baseline measurements (50ml; 5 dessertspoons)
- Once the study begins, participants will be randomly allocated to consume the probiotic supplement or a placebo (sucrose and matodextrin) for 12 weeks. Neither yourself or investigator will know which of the supplements you are taking. After the first 12 weeks (first treatment) there will be a 4 week washout period where no product will be consumed.
- A second 12 week treatment product different from the initial product will be then supplied.
- Participants will be required to consume the product as one dose at any time during the day but preferably in the morning. The products will be provided as a powder in capsules, therefore can be kept at room temperature, and also can be taken abroad.
- Participants will provide one stool sample on weeks 0, 6, 12, 16, 22, 28 and 32 of the study to look for changes in gut bacteria. The same times will be used for blood sampling to assess blood lipids (cholesterol etc.) and certain immune parameters.

- Volunteers will be given a container for faecal collection and shown to private facilities within the department to give a stool sample. If unable to do so they will be given the option to come in on preceding days until production is possible. No new treatment will be issued until stool has been provided.
- All blood samples will be taken in the Hugh Sinclair unit of the Department of Food and Nutritional Sciences

- **Maintenance of normal diet patterns throughout the study is essential** and participants will be required to complete food and drink logs throughout the study

University of Reading

Department of Food and Nutritional Sciences
Adele Costabile: a.costabile @.reading.ac.uk
Whiteknights
PO Box 266, Reading RG6 6AP, UK
phone 0118 378 6218
fax +44 (0)118 931 0080

- Any adverse medical events which occur during the trial (e.g. headache, gut symptoms) should be recorded in a diary along with medication taken
- All incidence of respiratory infections and colds should also be reported
- Daily stool habit should be recorded in a diary
- Please note that participants will be removed from the study if they develop acute gastrointestinal illness (e.g. food poisoning) or intolerance to the supplement or if they do not comply to above stated restrictions

Are there any risks?

The product used in this study will be the probiotic *Lactobacillus plantarum* 2830, the placebo will be sucrose and maltodextrin served in capsules, twice daily. *Lactobacillus plantarum* 2830, sucrose and maltodextrin do not pose any risk to participants.

Blood samples will be collected by experienced staff trained for this purpose at the University (Hugh Sinclair Unit). There is a chance of a little discomfort and bruising when having blood taken but every care will be taken to minimise this.

Confidentiality

Confidentiality will be maintained by allocating volunteers an identification code, which will be used to identify all samples and data obtained. Volunteer's names will not be used in any reports or publications. All data generated from the study will be held securely within a password protected file, only the study investigators will have access to this a record of the names of the volunteers will not be held on the same file.

Information matching volunteer names with identification codes will be kept by a departmental secretary in a locked filing cabinet, the investigators will only use identification codes. The only time data will be matched with volunteer names is for those volunteers that request to have their personal results discussed with them. A request for individual results to be discussed will include a review of all sample results for the individual volunteer. A list of the names and addresses of the subjects in this project will be compiled, this, together with a copy of the Consent Form, will be retained within the School for a minimum of five years after the date that the project is completed.

Restrictions during testing

- Participants must not consume pre- or probiotic supplements or food products containing them during the study.

Examples of these food products are:

- Danone Actimel yoghurt drink
- Yakult milk drink
- Danone Activia yoghurt
- Kellogs rice crispies multigrain
- Weetabix weetaflakes
- Muller Vitality Yoghurt/Drinks

University of Reading

Department of Food and Nutritional Sciences
Adele Costabile: a.costabile @.reading.ac.uk
Whiteknights
PO Box 266, Reading RG6 6AP, UK
phone 0118 378 6218
fax +44 (0)118 931 0080

- Warburton's healthy inside bread

General Information

- You will receive £150 for completing the trial and £20 if you are a reserve volunteer who does not receive a treatment. Volunteers that drop out will have their payment pro-rated to cover the part of the study completed.
- Stool sample pots will be provided and advice on how to take stool samples will be given
- The analysis of the faecal and blood samples will occur at the University of Reading
- You will be provided with breakfast on visit days
- If at any time you wish to withdraw from the study you are completely free to do so without giving a reason
  The information collected will be used for research purposes only. All information will be confidential and individuals' names will not be used in any reports resulting from this work.
- Once the study has been completed you can request your results
- All unused blood and faecal samples will be destroyed after the completion of the study and sample analysis.

The University has appropriate insurance and is well used to carrying out these types of trials. If there is a complain then this should be addressed to Professor Glenn R Gibson, FMSU, Universty of Reading g.r.gibson@reading.ac.uk

The investigators thank you for taking time to read this. If you have any queries please feel free to contact:

Adele Costabile a.costabile@ reading.ac.uk
or **Ivan Buttarazzi**

Department of Food and Nutritional Sciences
Adele Costabile: a.costabile @.reading.ac.uk
Whiteknights
PO Box 266, Reading RG6 6AP, UK
phone 0118 378 6218
fax +44 (0)118 931 0080

Study Timetable

Proposed dates	***Stage of Study***	*Treatment*
	**Baseline**<br>Pre-trial meeting/ Screening	• *Briefing about the study*<br>• Provide screening blood samples<br>• Dispensing food and drink diary cards
**Weeks 0**	**Visit 1**<br>Start of study	• Provide base-line faecal and blood samples<br>• Collecting food and drink diary cards<br>• Dispensing diary cards<br>• Dispensing product<br>• During the 12 week period – product will be taken daily<br>• Symptom card completed daily<br>• Diet diary completed over 4 days
**Weeks 1-6**<br>(interim period)<br>**&**<br>**Weeks 6-12**<br>(end of 1st treatment period)	**Visits 2 and 3**<br>**12 Week**<br>1st treatment period	• Diaries to be submitted<br>• Provide faecal and blood samples and given new diary cards. No product provided for washout period.
**Weeks 12-16**	**Visit 4**<br>**4 week**<br>Post Washout period	• Provide faecal and blood samples (visit 4)<br>• Diaries to be submitted<br>• Given new diary cards<br>• Diaries to be completed daily<br>• Food diaries to be completed over 4 days<br>• Provide faecal and blood samples and given new diary cards and product (2nd treatment)<br>• 2nd treatment product to be taken daily
**Weeks 16-22**<br>(interim period)<br>**&**<br>**Weeks 22-28**<br>(end of 2st treatment period)	**Visits 5 and 6**<br>**12 Week**<br>End of 2nd treatment period	• Diaries to be submitted<br>• Provide faecal and blood samples and given new diary cards.<br>• Food diaries to be completed over 4 days<br>• Provide faecal and blood samples and collect any unused product)

University of Reading

Department of Food and Nutritional Sciences
Adele Costabile: a.costabile @.reading.ac.uk
Whiteknights
PO Box 266, Reading RG6 6AP, UK
phone 0118 378 6218
fax +44 (0)118 931 0080

**Weeks 28-32**	**Visit 7**<br>**4 week**<br>Post Washout period	• Provide faecal and blood samples (visit 7)<br>• Provide faecal and blood (2nd treatment)

Note: Volunteers will take all treatments during the course of the study but not at the same time. Volunteers will be provided with breakfast on visit days.

University of Reading

Department of Food and Nutritional Sciences
Adele Costabile: a.costabile @.reading.ac.uk
Whiteknights
PO Box 266, Reading RG6 6AP, UK
phone 0118 378 6218
fax +44 (0)118 931 0080

FOODS TO AVOID

Whilst participating in this trial there are some foods that we would ask you to please refrain from consuming

Probiotics

- (found in many yogurts)

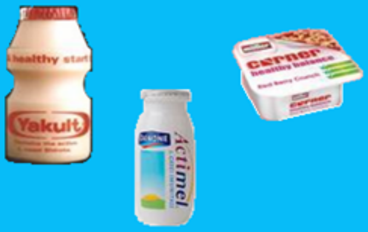

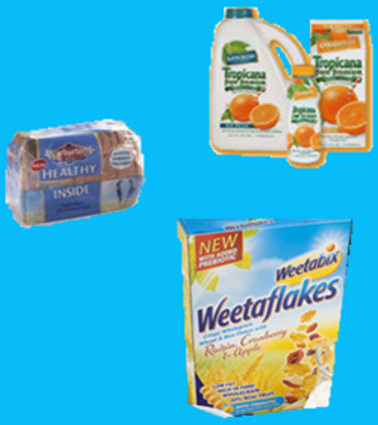

Additional **Prebiotics**

Added to

-Supplemented bread

-Supplemented Cereals

-Supplemented fruit juices

-Additional supplements

Treatment 2

Treatment 1

WHOLE GRAIN

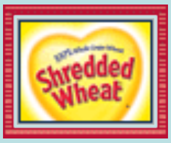

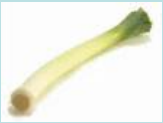

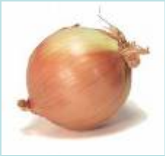

Department of Food and Nutritional Sciences
Adele Costabile: a.costabile @.reading.ac.uk
Whiteknights
PO Box 266, Reading RG6 6AP, UK
phone 0118 378 6218
fax +44 (0)118 931 0080

Appendix F: Pre-study Questionnaire

Pre-study General Health Questionnaire and Volunteer details

Name: ____

DOB: ____

Gender: ____

Address: ______________________________

____

____

____

____

Telephone: _____________________________________

Height: ____ (cm)

Weight: ____ (kg)

Waist circumference: _____________________________ (cm)

Department of Food and Nutritional Sciences
Adele Costabile: a.costabile @.reading.ac.uk
Whiteknights
PO Box 266, Reading RG6 6AP, UK
phone 0118 378 6218
fax +44 (0)118 931 0080

Questions to assess general health

Does the following apply to you?

	Yes	No	Don't know	If Yes – Please describe
History of drug or alcohol abuse				
Excessive Alcohol consumption				
Smoker				
Lactose intolerance				
Pregnancy, lactation or planning pregnancy				
Involvement in drug/ medication study in last month				
Intake in pre or probiotics within last month				
Gluten allergy				
Use of antibiotics in previous 6 months				
Colonic irrigation (within last 3 months)?				
Medication active on GI tract (within last 3 months)				
Family history of colorectal cancer in under 50's				

Please state any prescribed medication you are currently taking below:

____

____

____

____

____

University of Reading

Department of Food and Nutritional Sciences
Adele Costabile: a.costabile @.reading.ac.uk
Whiteknights
PO Box 266, Reading RG6 6AP, UK
phone 0118 378 6218
fax +44 (0)118 931 0080

Do you suffer from any conditions involving the following?

	Yes	No	Don't know	If Yes – Please describe
Dermatological/Connective tissue				
Head, Ears, Eyes, Nose and Throat				
Cardiovascular				
Respiratory				
Abdominal				
Urogenital/Rectal				
Gastroenterological				
Lymphatic				
Neurological				
Coeliac disease				

All details will be kept strictly confidential

University of Reading

Department of Food and Nutritional Sciences
Adele Costabile: a.costabile @.reading.ac.uk
Whiteknights
PO Box 266, Reading RG6 6AP, UK
phone 0118 378 6218
fax +44 (0)118 931 0080

Appendix G: Volunteer diaries

Volunteer Diary

Page 1 of 10

Volunteer No. _______________________

Period No. _____________________ **Day No. to Day No.**

Please fill in the diary carefully and completely for each day. If you are unsure how to answer, please give the best information you can. Please return completed diary to Adele Costabile on your next visit.

To be filled in by investigator only!

Date started at:

Next visit at:

Department of Food and Nutritional Sciences
Adele Costabile: a.costabile @.reading.ac.uk
Whiteknights
PO Box 266, Reading RG6 6AP, UK
phone 0118 378 6218
fax +44 (0)118 931 0080

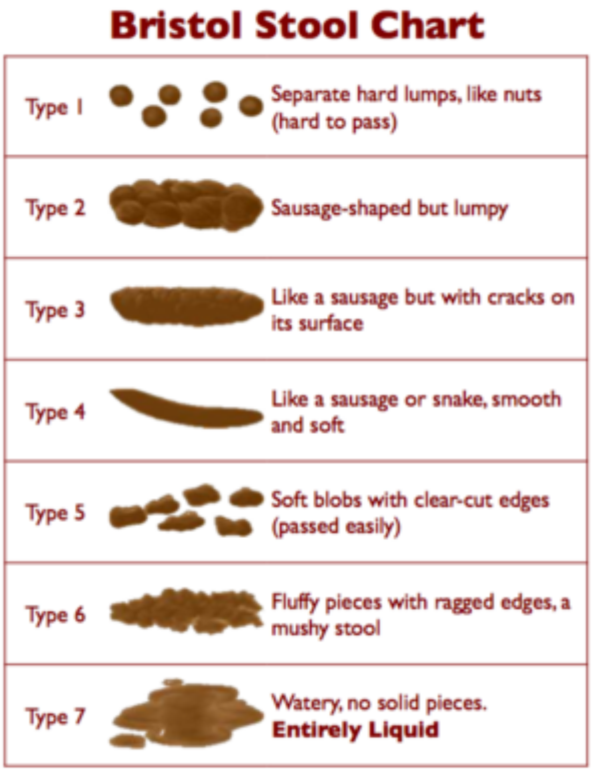

The Bristol stool chart provides a scale relating to stool consistency, please use this chart to rate your stool consistency 1-7 (solid – liquid) in your daily diary.

E.g. a rating of 4 – used in the diary example would relate to “like a sausage or snake, smooth and soft”)

University of Reading

Department of Food and Nutritional Sciences
Adele Costabile: a.costabile @.reading.ac.uk
Whiteknights
PO Box 266, Reading RG6 6AP, UK
phone 0118 378 6218
fax +44 (0)118 931 0080

 Volunteer No.

**Study day**	**Date d/m**	**Number of stools**	**Stool consistency as per Bristol chart (page 2)**			**Abdominal pain**			
		If 0 please include	Stool 1	Stool 2	Stool 3	**none**	**mild**	**moderate**	**severe**
e.g.	15/01	1	4	x	x	▪			
1									
2									
3									
4									
5									
6									
7									
8									
9									
10									
11									
12									
13									
14									
15									
16									
17									
18									
19									
20									
21									

University of Reading

Department of Food and Nutritional Sciences
Adele Costabile: a.costabile @.reading.ac.uk
Whiteknights
PO Box 266, Reading RG6 6AP, UK
phone 0118 378 6218
fax +44 (0)118 931 0080

 Volunteer No.

**Study day**	**Date d/m**	**Stomach or intestinal bloating**				**Flatulence**			
		**none**	**mild**	**moderate**	**severe**	**none**	**mild**	**moderate**	**severe**
**e.g.**	**15/01**	▪				▪			
1									
2									
3									
4									
5									
6									
7									
8									
9									
10									
11									
12									
13									
14									
15									
16									
17									
18									
19									
20									
21									

Department of Food and Nutritional Sciences
Adele Costabile: a.costabile @.reading.ac.uk
Whiteknights
PO Box 266, Reading RG6 6AP, UK
phone 0118 378 6218
fax +44 (0)118 931 0080

 Volunteer No. _______________

**Study day**	**Date d/m**	**Happy**			**Alert**		
		**Less than normal**	**Normal**	**More than normal**	**Less than normal**	**Normal**	**More than normal**
**e.g.**	**15/1**		▪			▪	
1							
2							
3							
4							
5							
6							
7							
8							
9							
10							
11							
12							
13							
14							
15							
16							
17							
18							
19							
20							
21							

 Volunteer No. _______________

University of Reading

Department of Food and Nutritional Sciences
Adele Costabile: a.costabile @.reading.ac.uk
Whiteknights
PO Box 266, Reading RG6 6AP, UK
phone 0118 378 6218
fax +44 (0)118 931 0080

**Study day**	**Date d/m**	**Energetic**			**Stressed**		
		**Less than normal**	**Normal**	**More than normal**	**Less than normal**	**Normal**	**More than normal**
**e.g**	**15/01**		▪			▪	
1							
2							
3							
4							
5							
6							
7							
8							
9							
10							
11							
12							
13							
14							
15							
16							
17							
18							
19							
20							
21							

Department of Food and Nutritional Sciences
Adele Costabile: a.costabile @.reading.ac.uk
Whiteknights
PO Box 266, Reading RG6 6AP, UK
phone 0118 378 6218
fax +44 (0)118 931 0080

 Volunteer No. _______________

Medication

Please enter the intake of any medication taken during the study with name, dosage per day, date started and date stopped.

**Medication**	**Dosage**	**Date started - stopped**
**e.g1. paracetamol**	**500mg twice**	**15/1 – 15/01**
1		
2		
3		
4		
5		
6		
7		
8		
9		
10		
11		
12		
13		
14		
15		
16		
17		
18		
19		
20		
21		

 Volunteer No. _______________

Department of Food and Nutritional Sciences
Adele Costabile: a.costabile @.reading.ac.uk
Whiteknights
PO Box 266, Reading RG6 6AP, UK
phone 0118 378 6218
fax +44 (0)118 931 0080

Adverse Events

Please enter the occurrence of all adverse events and any respiratory infections, colds or flu-like symptoms with start and end date and time.

**Adverse Event**	**Date – started**	**Date – stopped**
**e.g1. headache**	**15/01**	**15/01**
1		
2		
3		
4		
5		
6		
7		
8		
9		
10		
11		
12		
13		
14		
15		
16		
17		
18		
19		
20		
21		

Department of Food and Nutritional Sciences
Adele Costabile: a.costabile @.reading.ac.uk
Whiteknights
PO Box 266, Reading RG6 6AP, UK
phone 0118 378 6218
fax +44 (0)118 931 0080

 Volunteer No. _______________

Date:

**Time**	**Food/drink**	**Details**	**Amount**
E.g. 6.30			
7.30	Cereal	Weetabix	3
	Semi-skimmed milk		1 mug full
	Toast	Wholemeal	2 slices
	Tea	Tea, Milk	1 mug full

University of Reading

Department of Food and Nutritional Sciences
Adele Costabile: a.costabile @.reading.ac.uk
Whiteknights
PO Box 266, Reading RG6 6AP, UK
phone 0118 378 6218
fax +44 (0)118 931 0080

 Volunteer No. _______________

Final Questions

Did you experience any of the following after taking your product:

Did you experience an after taste?

Yes______ No______

Did you experience a feeling of fullness?

Yes______ No______

Did you experience difficulty in taking the product?

Yes______ No______

University of Reading

Department of Food and Nutritional Sciences
Adele Costabile: a.costabile @.reading.ac.uk
Whiteknights
PO Box 266, Reading RG6 6AP, UK
phone 0118 378 6218
fax +44 (0)118 931 0080

Appendix H: Advertisement

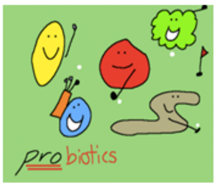

Probiotic Approach to Cholesterol Reduction

We are recruiting volunteers for a study investigating the effects of a beneficial bacteria on cholesterol levels and immunity
We are looking for:
men and women with slightly raised cholesterol (aged 30-65 years)

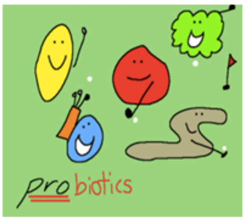

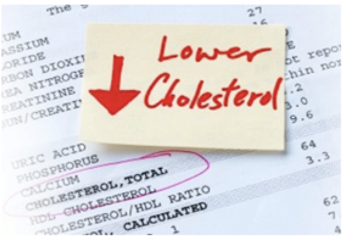

The study will start in January 2015
If you are interested
please contact
Adele Costabile: a.costabile@reading.ac.uk;
Department of Food and Nutritional Sciences
Whiteknights, PO Box 266, Reading RG6 6AP,
UK Phone: 0118 378 6218- 07713087948

Department of Food and Nutritional Sciences
Adele Costabile: a.costabile @.reading.ac.uk
Whiteknights
PO Box 266, Reading RG6 6AP, UK
phone 0118 378 6218
fax +44 (0)118 931 0080

Appendix I

Sensory Dimensions Recruitment Letter

Dear

We are looking to recruit men and women aged 30-65 years old, who have slightly raised cholesterol, on to a dietary intervention trial at The University of Reading. The product we will be using is a probiotic called *Lactobacillus plantarum*. Probiotics are positive gut bacteria. This trial will investigate the effect of the probiotic on gut bacteria, blood lipids (including cholesterol) and the influence of this product on immune status.

The study will consist of a 8 month randomised double blind crossover treatment period with probiotic and placebo.

Please review the enclosed information sheet and contact myself (study investigator) if you are interested in participating, or would like any further information or to discuss any concerns. There will be a minimum of two weeks to decide whether you would like to be involved in the study and a meeting will be arranged before the commencement of the trial prior to giving informed consent. Participants are free to withdraw from the trial at any time.

The trial will commence on (*Trial start date)*

If you have any questions about the trial please do not hesitate to contact me.

We appreciate your help.

Yours sincerely,

Adele Costabile

University of Reading

Department of Food and Nutritional Sciences
Adele Costabile: a.costabile @.reading.ac.uk
Whiteknights
PO Box 266, Reading RG6 6AP, UK
phone 0118 378 6218
fax +44 (0)118 931 0080

Appendix J

Information relating to safety aspects of the probiotic

Please find attached the pdf file from Nizo confirming all strains are QPS and GRAS. The strains all come from Nizo's strain collection and have been used in some way in food processing or manufacture and therefore regularly fed to humans with no side effects. The details of each strain is in the Word document although specifics have to be removed to preserve commercial confidence. We are using strain 2830 which has been used in fruit juice fermentations. In addition to the attached I have full genetic sequencing of the strains and can confirm the absence of any pathogenicity, virulence, or toxin, or antibiotic resiatnce factors. The preclinical studies have confirmed that *Lactobacillus plantarum* 2830 grows well, can be freeze dried, can survive gut acidity & bile acids, and produces large amounts of BSH and so has been chosen to progress. This strain is currently being produced, capsulated, and packaged for the commencement of the studies in January 2015. Any concerns/questions please feel free to call at any time.

Best Wishes,
Stephen OHara
email: spohara@hotmail.com
Mobile: 07887 874392

University of Reading

Department of Food and Nutritional Sciences
Adele Costabile: a.costabile @.reading.ac.uk
Whiteknights
PO Box 266, Reading RG6 6AP, UK
phone 0118 378 6218
fax +44 (0)118 931 0080

Regarding the assigned strains to Optibiotix coming from the NIZO Culture Collection below we can state that all these strains are non-GMO strains as they are from a natural origin and have not been modified in any way after isolation.
The antibiotic and virulence databases do not give any good hits with your genomes. There are cases where a low (very significant) e- value is found but this is never for a full gene, typically not more than 25% of the length. In essence there are no virulence and antibiotic resistance genes in your genomes that have a high identity (>75%) over a large part of a gene (>75%). From that we would conclude the strains are safe to the best of our knowledge.

All the best,
NIZO food research BV
Dr. P.A. (Peter) Bron
Senior Scientist

Name		NIZO Code	Isolated from	Ownership	Other
Lactobacillus	*plantarum*	NIZO2828	Source not documented	NIZO	in NCC before 1993, used in fruit juice fermentations
Lactobacillus	*plantarum*	NIZO2830	Source not documented	NIZO	in NCC before 1993, used in fruit juice fermentations
Lactobacillus	*plantarum*	NIZO2691	Farm yoghurt from U.S.	NIZO	in NCC before 1993, used in melon and tomato fermentations

Department of Food and Nutritional Sciences
Adele Costabile: a.costabile @.reading.ac.uk
Whiteknights
PO Box 266, Reading RG6 6AP, UK
phone 0118 378 6218
fax +44 (0)118 931 0080

NIZO food research B.V.
Kernhemseweg 2, 6718 ZB Ede, The Netherlands
P.O. Box 20, 6710 BA Ede, The Netherlands
Chamber of Commerce 091 390 48
T +31 (0)318 65 95 11 F +31 (0)318 65 04 00
E info@nizo.com W www.nizo.com

Optibiotix Health PLC
Attn: Mr. S. OHara
Innovation Centre,
Innovation Way,
Heslington, York, YO10 5DG
UNITED KINGDOM

Date:	Our reference:	Your reference:	Direct number:
September 30, 2014	2014/GJH/NH/060		+31 318 659551

Subject
NIZO Culture Collection

Dear Mr. OHara,

Regarding the assigned strains to Optibiotix Health PLC from the NIZO Culture Collection (NCC) we are pleased to confirm the following information:

1. All strains are non-GMO strains as they are from a natural origin and have not been modified in any way after isolation
2. All strains have Qualified Presumption of Safety (QPS), a generic assessment system used by the European Food Standard Agency (EFSA) for the safety of microorganism introduced to the food chain

Further details on each strain are provided below:

Name		NIZO Code	Status	Isolated from	Ownership	Other
Lactobacillus	*plantarum*	NIZO2828	QPS	Source not documented	NIZO	in NCC before 1993
Lactobacillus	*plantarum*	NIZO2830	QPS	Source not documented	NIZO	in NCC before 1993
Lactobacillus	*plantarum*	NIZO2691	QPS	Farm yoghurt from U.S. for human consumption	NIZO	in NCC before 1993

With kind regards,
NIZO food research

Nils Hijlkema
Business Development Manager
