# Supplementary material for: An in vivo assessment of the cholesterol-lowering efficacy of Lactobacillus plantarum ECGC 13110402 in normal to mildly hypercholesterolaemic adults
Source: PLoS One. 2017 Dec 11;12(12):e0187964. doi: 10.1371/journal.pone.0187964 (PMC5724841; doi:10.1371/journal.pone.0187964)
Supplement: S3 Checklist — (PDF) [file pone.0187964.s003.pdf]

Introduction

A randomised control trial was performed to examine the cholesterol lowering efficacy of a Lactobacillus strain in hypercholesterolaemic adults. Subjects were randomised using a 1:1 allocation into either a placebo group (maltodexrin) or an active group (Lactobacillus strain).

Each study participant was measured on 4 occasions:

- 0 weeks (Baseline)
- 6 weeks (During treatment)
- 12 weeks (End of treatment)
- 16 weeks (Post-treatment)

The primary aim of the study was to compare the groups in terms of their lipid measurements.

Statistical Methods

An initial set of analyses examined the demographic and outcome variables at baseline to ensure that the two groups were well matched. Continuous variables were analysed using the unpaired t-test, whilst the Chi-square test was used for the categorical variables.

The next analyses compared the study outcomes between the two study groups in terms of changes between timepoints The change in outcomes over four pre-determined study periods was examined. The periods examined were:

- Baseline to 6 weeks
- 6 weeks to 12 weeks
- Baseline to 12 weeks
- 12 weeks to 16 weeks

All outcomes were continuous in nature. For each analysis, the data was restricted to the two timepoints in the analysis (e.g. only the baseline and 6 week data was used to compare the changes from baseline to 6 weeks). The analyses were performed using analysis of covariance (ANCOVA). The latter timepoint was used as the outcome variable, with the earlier timepoint considered as a covariate. This approach is mathematically preferable to simply comparing the change over time between groups, as it potentially allows the change over time to very depend on the starting value.

The first set of analyses considered all study participants in the analysis. Subsequently, the lipid analyses were repeated for different patient subgroups, with subgroups created based on baseline total cholesterol, gender and age.

An additional set of analyses examined how the change in lipid values during each time period of interest was associated with the lipid levels at the start of the time period. Firstly the analysis was performed using data from both study groups combined and the association was examined using partial correlation, adjusting for study group (to allow for any differences in effect between groups). Subsequently Pearson correlation was used to examine the relationships for each group separately.

Results

a) Demographic/baseline variables

The first set of analyses compared the two study groups in terms of their demographics, and in terms of the outcome measures at baseline. A summary of the analysis results is given in Table 1. The figures show the mean and standard deviation in each group, aside from gender where the number and percentage in each group are reported. P-values indicating the significance of the results are also presented.

Table 1: Demographic and Baseline characteristics

Variable	Placebo (n=23) Mean (SD)	Active (n=23) Mean (SD)	P-value
Age	52.0 (8.4)	52.3 (10.7)	0.89
Gender (*) - Female	14 (61%)	18 (78%)	0.20
- Male	9 (39%)	5 (22%)	
Total cholesterol	5.22 (0.92)	5.10 (0.71)	0.62
HDL cholesterol	1.24 (0.31)	1.40 (0.35)	0.10
LDL cholesterol	3.44 (0.76)	3.20 (0.68)	0.28
Triglycerides	1.18 (0.45)	1.11 (0.46)	0.61
Weight	79.2 (16.5)	72.1 (12.0)	0.10
BMI	26.8 (5.0)	26.7 (3.7)	0.96
Waist	92.3 (13.5)	89.6 (12.0)	0.49
Systolic BP	118.7 (16.0)	119.2 (13.2)	0.73
Diastolic BP	71.0 (12.2)	73.0 (8.0)	0.52

(*) Number (%) reported

The results suggested no significant difference between the two study groups in terms of their demographics (age, sex) or for any of the lipid or anthropometric measures at baseline.

b) Lipid outcomes - All subjects

Analyses were performed to examine how the changes in lipid measurements over the course of the study varied between the two study groups. The changes between four pairs of timepoints were examined, with results summarised in Tables 2-5. These tables show the mean and standard deviation at each of the timepoints, and also in terms of the change between timepoints. The group differences from the ANCOVA analyses are also reported, with the figures the mean difference and corresponding confidence interval. These are reported as outcome for Active group minus outcome for Placebo group. [Note that due to the nature of the analyses the mean group difference may not equate to the difference in raw changes over time between groups]. P-values indicating the significance of the results are also reported.

Table 2: Change in Lipid measurements in all subjects from baseline to 6 weeks

Outcome	Group	Baseline Mean (SD)	6 weeks Mean (SD)	Change Mean (SD) [range]	% Change Mean (SD) [range]	Group Difference (*) Mean (95% CI)	P-value
TC	Placebo	5.22 (0.92)	5.33 (0.99)	0.10 (0.65) [-0.8, 1.2]	-2.7 (13.2) [-14.8, 25.6]	0	
	Active	5.10 (0.71)	5.01 (0.66)	-0.09 (0.46) [-0.1, 1.2]	-1.3 (9.1) [-15.8, 16.7]	-0.22 (-0.55, 0.10)	0.18
HDL	Placebo	1.25 (0.31)	1.32 (0.30)	0.06 (0.14) [-0.2, 0.4]	6.4 (14.2) [-15.4, 50.0]	0	
	Active	1.40 (0.35)	1.45 (0.42)	0.04 (0.16) [-0.2, 0.4]	2.8 (11.8) [-25.0, 22.2]	-0.02 (-0.12, 0.08)	0.67
LDL	Placebo	3.44 (0.77)	3.49 (0.81)	0.05 (0.51) [-0.7, 0.9]	2.7 (17.0) [-18.4, 37.5]	0	
	Active	3.20 (0.69)	3.06 (0.60)	-0.14 (0.49) [-1.6, 0.6]	-3.2 (13.9) [-40.0, 20.7]	-0.26 (-0.54, 0.03)	0.08
Trigs.	Placebo	1.20 (0.45)	1.13 (0.48)	-0.06 (0.37) [-0.9, 0.9]	-1.9 (25.7) [-52.9, 56.3]	0	
	Active	1.11 (0.46)	1.08 (0.67)	-0.03 (0.38) [-0.6, 1.1]	-4.2 (31.5) [-50.0, 64.7]	0.03 (-0.20, 0.26)	0.79

(*) Calculated from ANCOVA analysis, adjusting for baseline value

There was no strong evidence of that the two groups varied in terms of changes from baseline to 6 weeks.

Table 3: Change in Lipid measurements in all subjects from 6 weeks to 12 weeks

Outcome	Group	6 weeks Mean (SD)	12 wks Mean (SD)	Change Mean (SD) [range]	% Change Mean (SD) [range]	Group Difference (*) Mean (95% CI)	P-value
TC	Placebo	5.33 (0.99)	5.33 (0.84)	0.00 (0.72) [-1.4, 1.5]	1.3 (13.5) [-25.5, 32.6]	0	
	Active	5.01 (0.66)	5.12 (0.87)	0.11 (0.60) [-1.3, 1.5]	2.4 (11.0) [-22.4, 27.3]	0.02 (-0.36, 0.39)	0.93
HDL	Placebo	1.32 (0.30)	1.26 (0.28)	-0.06 (0.18) [-0.3, 0.4]	-3.7 (12.8) [-21.4, 36.4]	0	
	Active	1.45 (0.42)	1.46 (0.42)	0.01 (0.13) [-0.2, 0.2]	1.2 (9.7) [-18.2, 22.2]	0.09 (0.00, 0.18)	0.06
LDL	Placebo	3.49 (0.81)	3.58 (0.70)	0.09 (0.53) [-0.8, 1.1]	4.4 (16.2) [-21.2, 35.5]	0	
	Active	3.06 (0.60)	3.13 (0.78)	0.07 (0.48) [-1.1, 1.1]	2.6 (14.0) [-21.2, 35.5]	-0.11 (-0.41, 0.20)	0.49
Trigs.	Placebo	1.13 (0.48)	1.20 (0.40)	0.07 (0.44) [-1.2, 1.0]	13.1 (32.2) [-48.0, 83.3]	0	
	Active	1.08 (0.67)	1.15 (0.65)	0.07 (0.28) [-0.4, 0.8]	11.1 (26.8) [-33.3, 57.1]	-0.01 (-0.21, 0.20)	0.93

(*) Calculated from ANCOVA analysis, adjusting for 6 week value

There were no strong evidence of any group difference for the lipid variables for the period from 6 to 12 weeks.

Table 4: Change in Lipid measurements in all subjects from baseline to 12 weeks

Outcome	Group	Baseline Mean (SD)	12 weeks Mean (SD)	Change Mean (SD) [range]	% Change Mean (SD) [range]	Group Difference (*) Mean (95% CI)	P-value
TC	Placebo	5.22 (0.92)	5.33 (0.84)	0.11 (0.66) [-1.0, 1.4]	3.1 (13.4) [-14.6, 34.1]	0	
	Active	5.10 (0.71)	5.12 (0.87)	0.02 (0.56) [-1.3, 1.2]	0.6 (10.5) [-22.4, 23.3]	-0.12 (-0.47, 0.24)	0.51
HDL	Placebo	1.24 (0.31)	1.24 (0.29)	0.00 (0.17) [-0.2, 0.5]	1.5 (17.0) [-14.3, 62.5]	0	
	Active	1.40 (0.35)	1.46 (0.42)	0.06 (0.15) [-0.1, 0.5]	3.4 (10.5) [-12.5, 33.3]	0.06 (-0.04, 0.16)	0.23
LDL	Placebo	3.44 (0.76)	3.54 (0.70)	0.10 (0.62) [-0.9, 1.3]	4.9 (19.5) [-22.9, 52.0]	0	
	Active	3.20 (0.69)	3.13 (0.78)	-0.07 (0.53) [-1.3, 1.0]	-1.4 (15.6) [-36.4, 26.3]	-0.24 (-0.56, 0.09)	0.15
Trigs.	Placebo	1.18 (0.45)	1.20 (0.39)	0.03 (0.41) [-0.9, 0.6]	10.9 (35.7) [-52.9. 83.3]	0	
	Active	1.11 (0.46)	1.15 (0.65)	0.04 (0.36) [-0.5, 0.8]	3.6 (34.8) [-55.6, 114.3]	0.01 (-0.22, 0.23)	0.96

(*) Calculated from ANCOVA analysis, adjusting for baseline value

There were no statistically significant differences between the two study groups for the change from baseline to 12 weeks.

Table 5: Change in Lipid measurements in all subjects from 12 weeks to 16 weeks

Outcome	Group	12 weeks Mean (SD)	16 weeks Mean (SD)	Change Mean (SD) [range]	% Change Mean (SD) [range]	Group Difference (*) Mean (95% CI)	P-value
TC	Placebo	5.33 (0.84)	5.20 (0.94)	-0.13 (0.56) [-1.1, 0.8]	-2.2 (10.8) [-20.0, 17.8]	0	
	Active	5.04 (0.78)	5.04 (0.68)	0.0 (0.50) [-1.0, 0.8]	0.7 (9.4) [-13.9, 16.0]	0.08 (-0.23, 0.39)	0.61
HDL	Placebo	1.24 (0.29)	1.23 (0.29)	0.00 (0.12) [-0.2, 0.2]	0.3 (11.3) [-15.4, 25.0]	0	
	Active	1.48 (0.42)	1.46 (0.39)	-0.02 (0.16) [-0.4, 0.2]	-0.1 (10.8) [-20.0, 22.2]	0.02 (-0.07, 0.10)	0.70
LDL	Placebo	3.54 (0.70)	3.40 (0.71)	-0.13 (0.48) [-0.9, 0.7]	-2.8 (13.6) [-22.9, 23.3]	0	
	Active	3.04 (0.65)	3.08 (0.55)	0.05 (0.44) [-0.8, 0.9]	3.0 (14.8) [-16.7, 42.9]	0.04 (-0.23, 0.31)	0.79
Trigs.	Placebo	1.20 (0.39)	1.22 (0.51)	0.02 (0.46) [-0.7, 1.4]	4.3 (37.1) [-55.6, 107.7]	0	
	Active	1.14 (0.66)	1.09 (0.57)	-0.05 (0.33) [-0.8, 0.6]	1.5 (30.1) [-44.4, 75.0]	-0.08 (-0.31, 0.14)	0.47

(*) Calculated from ANCOVA analysis, adjusting for 12 week value

There was no evidence of a difference in lipid measurements between the two groups for the period for 12 to 16 weeks.

c) Anthropometric data - All subjects

A similar set of analyses were performed to compare the anthropometric variables between the two study groups. The results are summarised in Tables 6-9.

Table 6: Change in Anthropometric measurements from in all subjects baseline to 6 weeks

Outcome	Group	Baseline Mean (SD)	6 weeks Mean (SD)	Change Mean (SD) [range]	% Change Mean (SD) [range]	Group Difference (*) Mean (95% CI)	P-value
Weight	Placebo	79.2 (16.5)	79.4 (16.2)	0.2 (1.2) [-2.6, 2.3]	0.3 (1.5) [-2.5-, 3.3]	0	
	Active	72.1 (12.0)	72.6 (12.5)	0.5 (1.3) [-2.0, 3.5]	0.6 (1.8) [-2.7, 4.1]	0.3 (-0.5, 1.1)	0.39
BMI	Placebo	26.8 (5.0)	26.9 (5.0)	0.1 (1.0) [-3.0, 2.3]	0.5 (3.3) [-9.0, 8.1]	0	
	Active	26.7 (3.7)	26.9 (3.8)	0.2 (0.6) [-0.8, 1.4]	0.9 (2.3) [-2.7, 5.2]	0.1 (-0.3, 0.6)	0.57
Waist	Placebo	92.3 (13.5)	91.3 (13.3)	-1.0 (5.1) [-11, 9]	-1.0 (5.5) [13.6, 9.7]	0	
	Active	89.6 (12.0)	90.0 (11.3)	0.3 (4.3) [-9, 11]	0.6 (5.2) [-11.3, 13.8]	1.1 (-1.7, 3.8)	0.44
Systolic	Placebo	117.7 (16.0)	116.2 (16.0)	-1.5 (8.9) [-23, 15]	-0.9 (7.6) [-20.2, 14.9]	0	
	Active	119.2 (13.2)	121.0 (18.4)	2.2 (8.3) [-15, 16]	7.0 (7.0) [-13.4, 13.4]	3.7 (-1.6, 8.9)	0.17
Diastolic	Placebo	71.0 (12.2)	72.3 (9.5)	1.2 (6.5) [-12, 13]	2.8 (9.7) [-13.5, 22.0]	0	
	Active	73.0 (8.0)	71.9 (9.6)	-0.6 (7.7) [-10, 23]	-0.6 (11.8) [-15.4, 39.0]	-1.4 (-5.3, 2.5)	0.47

(*) Calculated from ANCOVA analysis, adjusting for baseline value

There was no evidence of any group differences when the changes between the two groups for the period from baseline to 6 weeks.

Table 7: Change in Anthropometric measurements in all subjects from 6 weeks to 12 weeks

Outcome	Group	6 weeks Mean (SD)	12 weeks Mean (SD)	Change Mean (SD) [range]	% Change Mean (SD) [range]	Group Difference (*) Mean (95% CI)	P-value
Weight	Placebo	79.4 (16.2)	79.3 (16.8)	0.0 (1.3) [-3.0, 2.8]	-0.2 (1.7) [-3.8, 2.8]	0	
	Active	72.6 (12.5)	72.8 (12.6)	0.2 (1.1) [-2.6, 2.1]	0.2 (1.5) [-3.8, 3.3]	0.3 (-0.4, 1.1)	0.34
BMI	Placebo	26.9 (5.0)	27.0 (5.2)	0.1 (1.0) [-1.2, 3.7]	0.5 (3.5) [-4.8, 13.4]	0	
	Active	26.9 (3.8)	27.2 (4.0)	0.3 (0.8) [-0.7, 3.3]	1.0 (2.9) [-2.5, 11.8]	0.1 (-0.4, 0.7)	0.57
Waist	Placebo	91.3 (13.3)	90.5 (13.8)	-0.8 (2.7) [-8, 4]	-0.9 (2.8) [-7.1, 3.9]	0	
	Active	90.0 (11.3)	89.1 (11.0)	-0.9 (3.5) [-13, 6]	-0.9 (3.9) [-13.0, 8.1]	-0.1 (-2.0, 1.8)	0.90
Systolic	Placebo	116.2 (16.0)	122.3 (11.4)	6.1 (7.8) [-17, 22]	5.9 (6.7) [-9.9, 22.9]	0	
	Active	121.0 (18.4)	119.7 (13.0)	-1.8 (10.5) [-35, 11]	-0.7 (7.4) [-22.2, 11.3]	-6.0 (-9.9, -2.1)	**0.003**
Diastolic	Placebo	72.3 (9.5)	73.5 (8.2)	1.2 (5.6) [-15, 13]	2.1 (7.5) [-14.4, 21.3]	0	
	Active	71.9 (9.6)	73.0 (8.2)	0.6 (5.6) [-11, 9]	1.4 (7.9) [-13.6, 15.7]	-0.7 (-3.6, 2.2)	0.62

(*) Calculated from ANCOVA analysis, adjusting for 6 week value

The study groups were found to vary significantly in their systolic blood pressure for the period from 6 to 12 weeks. There was increase in the placebo group, but a slight decrease in the active group. The values at 12 weeks were found to be 6 mmHg lower in the active group than in the placebo group.

No other differences between the two study groups were observed during the 6-12 week period.

Table 8: Change in Anthropometric measurements in all subjects from baseline to 12 weeks

Outcome	Group	Baseline Mean (SD)	12 weeks Mean (SD)	Change Mean (SD) [range]	% Change Mean (SD) [range]	Group Difference (*) Mean (95% CI)	P-value
Weight	Placebo	79.2 (16.5)	79.3 (16.8)	0.2 (1.7) [-2.6, 3.5]	0.1 (2.1) [-3.3, 4.7]	0	
	Active	72.1 (12.0)	72.8 (12.6)	0.7 (1.7) [-2.6, 3.8]	0.9 (2.2) [-2.8, 4.9]	0.7 (-0.3, 1.7)	0.18
BMI	Placebo	26.8 (5.0)	27.0 (5.2)	0.3 (1.3) [-3.1, 4.2]	0.9 (4.7) [-9.3, 15.5]	0	
	Active	26.7 (3.7)	27.2 (4.0)	0.5 (0.9) [-1.1, 3.3]	2.0 (3.3) [-3.9, 11.8]	0.3 (-0.4, 1.0)	0.41
Waist	Placebo	92.3 (13.5)	90.5 (13.8)	-1.8 (6.4) [-14, 12]	-1.8 (6.8) [-17.3, 12.9]	0	
	Active	89.6 (12.0)	89.1 (11.0)	-0.5 (5.7) [-13, 13]	-0.2 (6.7) [-13.0, 16.3]	0.9 (-2.6, 4.4)	0.61
Systolic	Placebo	117.7 (16.0)	122.3 (11.4)	4.7 (11.0) [-13, 28]	4.9 (10.3) [-11.4, 31.1]	0	
	Active	119.2 (13.2)	119.7 (13.0)	0.5 (8.9) [-19, 21]	0.7 (7.2) [-13.4, 15.8]	-3.6 (-8.6, 1.4)	0.15
Diastolic	Placebo	71.0 (12.2)	73.5 (8.2)	2.4 (9.0) [-15, 18]	5.0 (13.6) [-14.4, 30.5]	0	
	Active	73.0 (8.0)	73.0 (8.2)	0.0 (5.9) [-9, 13]	0.3 (8.4) [-10.5, 20.3]	-1.6 (-5.2, 2.1)	0.39

(*) Calculated from ANCOVA analysis, adjusting for baseline value

There was no strong evidence of a difference in anthropometric measurements between groups from baseline to 12 weeks.

There was very weak evidence that the systolic blood pressure at 12 weeks was lower in the active group, but this difference was not statistically significant.

Table 9: Change in Anthropometric measurements in all subjects from 12 weeks to 16 weeks

Outcome	Group	12 weeks Mean (SD)	16 weeks Mean (SD)	Change Mean (SD) [range]	% Change Mean (SD) [range]	Group Difference (*) Mean (95% CI)	P-value
Weight	Placebo	79.3 (16.8)	79.0 (16.8)	-0.3 (0.6) [-1.7, 1.1]	-0.4 (0.8) [-2.3, 1.7]	0	
	Active	72.8 (12.6)	72.5 (12.6)	-0.3 (0.9) [-2.4, 1.6]	-0.4 (1.3) [-3.6, 2.4]	0.0 (-0.5, 0.5)	0.96
BMI	Placebo	27.0 (5.2)	27.0 (5.2)	-0.1 (1.1) [-3.6, 3.2]	-0.2 (3.8) [-11.5, 12.1]	0	
	Active	27.2 (4.0)	27.0 (3.9)	-0.3 (0.9) [-3.0, 0.9]	-1.0 (2.8) [-9.6, 3.9]	-0.2 (-0.8, 0.3)	0.44
Waist	Placebo	90.5 (13.8)	90.5 (14.9)	0.0 (4.5) [-8, 9]	-0.1 (5.0) [-10.1, 8.6]	0	
	Active	89.1 (11.0)	88.9 (11.8)	-0.2 (3.2) [-7, 5]	-0.2 (3.7) [-8.5, 5.7]	-0.1 (-2.5, 2.2)	0.91
Systolic	Placebo	122.3 (11.4)	118.0 (13.5)	-4.3 (9.5) [-25, 12]	-3.4 (7.4) [-19.1, 9.8]	0	
	Active	119.7 (13.0)	117.3 (15.4)	-2.3 (9.4) [-14, 29]	-1.9 (7.6) [-10.6, 23.6]	1.7 (-3.9, 7.4)	0.54
Diastolic	Placebo	73.5 (8.2)	71.2 (8.2)	-2.3 (6.9) [-20, 10]	-2.7 (8.9) [-22.2, 14.5]	0	
	Active	73.0 (8.2)	72.2 (8.0)	-0.8 (5.7) [-10, 10]	-0.8 (8.0) [-11.9, 14.1]	1.3 (-2.2, 4.8)	0.46

(*) Calculated from ANCOVA analysis, adjusting for 12 week value

The change in outcomes between the active and control groups were not significantly different for the period from 12 to 16 weeks.

d) Lipid outcomes - Baseline TC < 5.0 mmol/L

The next analyses concentrated on the subgroup with a baseline total cholesterol of less than 5.0 mmol/L. This group consisted of 23 subjects, 11 in control group and 12 in intervention group.

Analyses were performed to examine how the changes in lipid measurements over the course of the study varied between the two study groups. The changes between four pairs of timepoints were examined, with results summarised in Tables 10-13.

Table 10: Change in Lipid measurements in subjects with baseline TC < 5.0 mmol/L from baseline to 6 weeks

Outcome	Group	Baseline Mean (SD)	6 weeks Mean (SD)	Change Mean (SD) [range]	% Change Mean (SD) [range]	Group Difference (*) Mean (95% CI)	P-value
TC	Placebo	4.50 (0.28)	4.79 (0.54)	0.29 (0.63) [-0.6, 1.1]	6.9 (14.3) [-12.5, 25.6]	0	
	Active	4.53 (0.33)	4.52 (0.42)	-0.01 (0.46) [-0.7, 0.7]	0.1 (10.2) [-15.2, 16.7]	-0.27 (-0.70, 0.15)	0.19
HDL	Placebo	1.11 (0.29)	1.20 (0.22)	0.09 (0.15) [-0.1, 0.4]	10.8 (17.5) [-5.9, 50.0]	0	
	Active	1.34 (0.30)	1.39 (0.36)	0.05 (0.17) [-0.2, 0.3]	3.4 (11.6) [-13.3, 21.4]	-0.01 (-0.17, 0.14)	0.85
LDL	Placebo	2.82 (0.28)	3.02 (0.51)	0.20 (0.56) [-0.5, 0.9]	8.0 (20.8) [-17.9, 37.5]	0	
	Active	2.71 (0.31)	2.68 (0.36)	-0.03 (0.38) [-0.6, 0.6]	-0.2 (13.7) [-18.9, 20.7]	-0.30 (-0.70, 0.09)	0.13
Trigs.	Placebo	1.17 (0.52)	1.11 (0.40)	-0.06 (0.30) [-0.6, 0.4]	1.3 (24.8) [-35.3, 50.0]	0	
	Active	1.08 (0.39)	0.94 (0.48)	-0.14 (0.31) [-0.6, 0.5]	-12.4 (30.3) [-50.0, 62.5]	-0.10 (-0.36, 0.16)	0.42

(*) Calculated from ANCOVA analysis, adjusting for baseline value

There was no strong evidence of a statistical difference between that the two groups in terms of changes from baseline to 6 weeks.

Table 11: Change in Lipid measurements in subjects with baseline TC < 5.0 mmol/L from 6 weeks to 12 weeks

Outcome	Group	6 weeks Mean (SD)	12 wks Mean (SD)	Change Mean (SD) [range]	% Change Mean (SD) [range]	Group Difference (*) Mean (95% CI)	P-value
TC	Placebo	4.79 (0.54)	4.79 (0.43)	0.00 (0.70) [-1.4, 0.9]	1.2 (14.2) [-25.5, 22.5]	0	
	Active	4.52 (0.42)	4.61 (0.42)	0.09 (0.36) [-0.8, 0.5]	2.3 (7.5) [-16.3, 10.4]	-0.12 (-0.50, 0.26)	0.53
HDL	Placebo	1.20 (0.22)	1.14 (0.22)	-0.06 (0.12) [-0.3, 0.1]	-4.6 (8.8) [-21.4, 8.3]	0	
	Active	1.39 (0.36)	1.44 (0.42)	0.05 (0.13) [-0.2, 0.2]	3.0 (9.4) [-12.5, 15.4]	0.10 (-0.02, 0.22)	0.09
LDL	Placebo	3.02 (0.51)	3.19 (0.47)	0.17 (0.51) [-0.7, 0.9]	7.3 (17.4) [-21.2, 34.6]	0	
	Active	2.68 (0.36)	2.73 (0.28)	0.04 (0.29) [-0.7, 0.5]	2.3 (9.8) [-20.0, 19.2]	-0.32 (-0.64, 0.01)	0.05
Trigs.	Placebo	1.11 (0.40)	1.14 (0.40)	0.03 (0.22) [-0.3, 0.3]	5.0 (20.3) [-27.3, 30.0]	0	
	Active	1.08 (0.67)	1.15 (0.65)	0.03 (0.25) [-0.4, 0.4]	9.8 (26.8) [-33.3, 57.1]	-0.01 (-0.21, 0.20)	0.93

(*) Calculated from ANCOVA analysis, adjusting for 6 week value

There was some suggestion of difference for the change in LDL cholesterol during the period from 6 to 12 weeks. Both groups showed an increase in values during the time period, but this increase was lower in the active group. The values at 12 weeks (adjusting for 6 weeks) were 0.32 mmol/L lower in the active group.

Table 12: Change in Lipid measurements in subjects with baseline TC < 5.0 mmol/L **from baseline to 12 weeks**

Outcome	Group	Baseline Mean (SD)	12 weeks Mean (SD)	Change Mean (SD) [range]	% Change Mean (SD) [range]	Group Difference (*) Mean (95% CI)	P-value
TC	Placebo	4.50 (0.28)	4.79 (0.43)	0.29 (0.60) [-0.7, 1.4]	7.1 (13.7) [-14.6, 34.1]	0	
	Active	4.53 (0.33)	4.61 (0.42)	0.08 (0.38) [-0.5, 1.0]	2.0 (8.5) [-10.9, 23.3]	-0.19 (-0.56, 0.19)	0.31
HDL	Placebo	1.09 (0.28)	1.11 (0.23)	0.02 (0.19) [-0.2, 0.5]	4.5 (22.3) [-14.3, 62.5]	0	
	Active	1.34 (0.30)	1.44 (0.42)	0.10 (0.19) [-0.1, 0.5]	6.2 (12.9) [-12.5, 33.3]	0.09 (-0.10, 0.27)	0.33
LDL	Placebo	2.88 (0.33)	3.15 (0.47)	0.26 (0.59) [-0.8, 1.3]	10.5 (21.0) [-22.9, 52.0]	0	
	Active	2.71 (0.31)	2.72 (0.28)	0.02 (0.30) [-0.6, 0.6]	1.3 (11.7) [-21.4, 26.1]	-0.39 (-0.74, -0.04)	**0.03**
Trigs.	Placebo	1.14 (0.51)	1.15 (0.38)	0.02 (0.41) [-0.9, 0.5]	12.3 (37.3) [-52.9, 62.5]	0	
	Active	1.08 (0.39)	0.97 (0.35)	-0.12 (0.25) [-0.5, 0.2]	-8.1 (25.4) [-55.6, 28.6]	0.01 (-0.22, 0.23)	0.96

(*) Calculated from ANCOVA analysis, adjusting for baseline value

There was a statistically significant difference between the two study groups in terms of changes in LDL cholesterol from baseline to 12 weeks. LDL values rose in both groups over time, but the increase was lesser in the active group, with values, on average, 0.39 mmol/L lower in the active group.

There were no differences between groups for the three other lipid measurements.

Table 13: Change in Lipid measurements in subjects with baseline TC < 5.0 mmol/L from 12 weeks to 16 weeks

Outcome	Group	12 weeks Mean (SD)	16 weeks Mean (SD)	Change Mean (SD) [range]	% Change Mean (SD) [range]	Group Difference (*) Mean (95% CI)	P-value
TC	Placebo	4.79 (0.43)	4.65 (0.43)	-0.14 (0.57) [-1.1, 0.8]	-2.2 (11.7) [-20, 17.8]	0	
	Active	4.61 (0.42)	4.70 (0.47)	0.09 (0.42) [-0.6, 0.8]	2.3 (8.7) [-11.3, 16.0]	0.12 (-0.27, 0.50)	0.54
HDL	Placebo	1.11 (0.23)	1.13 (0.26)	0.02 (0.14) [-0.2, 0.2]	1.6 (13) [-15.4, 20.0]	0	
	Active	1.44 (0.42)	1.42 (0.39)	-0.03 (0.18) [-0.4, 0.2]	-0.5 (11.4) [-20, 14.3]	0.00 (-0.15, 0.16)	0.98
LDL	Placebo	3.15 (0.47)	3.04 (0.32)	-0.11 (0.45) [-0.8, 0.5]	-2 (14) [-22.9, 19.2]	0	
	Active	2.72 (0.28)	2.83 (0.41)	0.11 (0.33) [-0.4, 0.8]	4.2 (11.6) [-13.8, 25.8]	-0.02 (-0.35, 0.32)	0.93
Trigs.	Placebo	1.15 (0.38)	1.07 (0.44)	-0.08 (0.28) [-0.5, 0.3]	-5.8 (26.7) [-55.6, 37.5]	0	
	Active	0.97 (0.35)	1.01 (0.54)	0.04 (0.29) [-0.4, 0.6]	4.4 (33.8) [-44.4, 75.0]	0.14 (-0.12, 0.40)	0.26

(*) Calculated from ANCOVA analysis, adjusting for 12 week value

There was no evidence of a difference in lipid measurements between the two groups for the period for 12 to 16 weeks.

e) Lipid outcomes - Baseline TC 5.0 - 5.9 mmol/L

Similar analyses were also performed for the subgroup who had a baseline total cholesterol between 5.0 and 5.9 mmol/L. There were 17 subjects in this subgroup, 9 in the control group, and 8 in the intervention group

Analyses were performed to examine how the changes in lipid measurements over the course of the study varied between the two study groups. The changes between four pairs of timepoints were examined, with results summarised in Tables 14-17.

Table 14: Change in Lipid measurements in subjects with baseline TC between 5.0-5.9 mmol/L from baseline to 6 weeks

Outcome	Group	Baseline Mean (SD)	6 weeks Mean (SD)	Change Mean (SD) [range]	% Change Mean (SD) [range]	Group Difference (*) Mean (95% CI)	P-value
TC	Placebo	5.48 (0.25)	5.29 (0.52)	-0.19 (0.59) [-0.8, 0.9]	-3.2 (11.1) [-14.8, 18]	0	
	Active	5.55 (0.24)	5.49 (0.36)	-0.06 (0.34) [-0.9, 0.1]	-1.1 (6) [-15.8, 2.0]	0.18 (-0.30, 0.67)	0.43
HDL	Placebo	1.30 (0.26)	1.33 (0.34)	0.03 (0.16) [-0.2, 0.3]	2.2 (11.7) [-15.4, 20]	0	
	Active	1.50 (0.47)	1.58 (0.52)	0.08 (0.14) [-0.1, 0.3]	5.1 (10.4) [-8.3, 22.2]	0.03 (-0.14, 0.19)	0.74
LDL	Placebo	3.61 (0.27)	3.47 (0.38)	-0.14 (0.44) [-0.7, 0.6]	-3.6 (12.7) [-18.4, 18.8]	0	
	Active	3.55 (0.40)	3.33 (0.51)	-0.23 (0.57) [-1.6, 0.2]	-5.7 (14.3) [-40, 6.3]	-0.13 (-0.60, 0.35)	0.58
Trigs.	Placebo	1.24 (0.42)	1.08 (0.40)	-0.17 (0.35) [-0.9, 0.3]	-9.7 (22.8) [-52.9, 17.6]	0	
	Active	1.11 (0.58)	1.29 (0.95)	0.17 (0.45) [-0.2, 1.1]	7.9 (30.2) [-22.2, 64.7]	0.37 (-0.06, 0.79)	0.08

(*) Calculated from ANCOVA analysis, adjusting for baseline value

There was no strong evidence of that the two groups varied in terms of changes from baseline to 6 weeks. There was a suggestion that triglyceride values were higher in the active group than the placebo group, but this difference was not quite statistically significant.

Table 15: Change in Lipid measurements in subjects with baseline TC between 5.0-5.9 mmol/L from 6 weeks to 12 weeks

Outcome	Group	6 weeks Mean (SD)	12 wks Mean (SD)	Change Mean (SD) [range]	% Change Mean (SD) [range]	Group Difference (*) Mean (95% CI)	P-value
TC	Placebo	5.29 (0.52)	5.52 (0.64)	0.23 (0.65) [-0.8, 1.5]	4.9 (13.1) [-14.5, 32.6]	0	
	Active	5.49 (0.36)	5.25 (0.54)	-0.24 (0.56) [-1.3, 0.5]	-4.1 (9.8) [-22.4, 9.6]	-0.37 (-0.98, 0.25)	0.23
HDL	Placebo	1.33 (0.34)	1.31 (0.33)	-0.02 (0.23) [-0.3, 0.4]	-0.6 (17.6) [-20, 36.4]	0	
	Active	1.58 (0.52)	1.51 (0.49)	-0.06 (0.11) [-0.2, 0.1]	-3.8 (7.5) [-18.2, 7.1]	-0.01 (-0.20, 0.19)	0.92
LDL	Placebo	3.47 (0.38)	3.63 (0.54)	0.17 (0.52) [-0.5, 1.1]	5.4 (15.9) [-13.5, 35.5]	0	
	Active	3.33 (0.51)	3.16 (0.58)	-0.16 (0.5) [-1.1, 0.4]	-4.2 (15.5) [-34.4, 12.5]	-0.38 (-0.91, 0.15)	0.14
Trigs.	Placebo	1.08 (0.40)	1.29 (0.48)	0.21 (0.46) [-0.6, 1]	24.2 (36.5) [-30, 83.3]	0	
	Active	1.29 (0.95)	1.26 (0.92)	-0.03 (0.18) [-0.4, 0.2]	-1.1 (9.3) [-14.3, 12.5]	-0.21 (-0.59, 0.17)	0.25

(*) Calculated from ANCOVA analysis, adjusting for 6 week value

There were no strong evidence of any group difference for the lipid variables for the period from 6 to 12 weeks.

There was very weak evidence of a group difference for LDL cholesterol, although the difference was not statistical significant. LDL values were lower in the active group, on average 0.38 mmol/L lower than the placebo group.

Table 16: Change in Lipid measurements in subjects with baseline TC between 5.0-5.9 mmol/L from baseline to 12 weeks

Outcome	Group	Baseline Mean (SD)	12 weeks Mean (SD)	Change Mean (SD) [range]	% Change Mean (SD) [range]	Group Difference (*) Mean (95% CI)	P-value
TC	Placebo	5.48 (0.25)	5.52 (0.64)	0.04 (0.74) [-0.8, 1.2]	1.1 (13.8) [-14.5, 22.2]	0	
	Active	5.55 (0.24)	5.25 (0.54)	-0.30 (0.64) [-1.3, 0.6]	-5.2 (11.4) [-22.4, 11.8]	-0.23 (-0.87, 0.40)	0.44
HDL	Placebo	1.30 (0.26)	1.31 (0.33)	0.01 (0.15) [-0.2, 0.2]	0.3 (11.2) [-12.5, 15.4]	0	
	Active	1.50 (0.47)	1.51 (0.49)	0.01 (0.1) [-0.1, 0.2]	0.6 (6.4) [-8.3, 11.8]	-0.01 (-0.15, 0.14)	0.91
LDL	Placebo	3.61 (0.27)	3.63 (0.54)	0.02 (0.67) [-0.8, 1.1]	1.5 (19.2) [-21.1, 33.3]	0	
	Active	3.55 (0.40)	3.16 (0.58)	-0.39 (0.65) [-1.3, 0.6]	-10.2 (18.3) [-36.4, 18.8	-0.47 (-1.08, 0.13)	0.11
Trigs.	Placebo	1.24 (0.42)	1.29 (0.48)	0.04 (0.46) [-0.9, 0.6]	10.7 (39.3) [-52.9, 83.3]	0	
	Active	1.11 (0.58)	1.26 (0.92)	0.15 (0.38) [-0.3, 0.7]	5.9 (26.4) [-33.3, 41.2]	0.13 (-0.33, 0.58)	0.56

(*) Calculated from ANCOVA analysis, adjusting for baseline value

There were no statistically significant differences between the two study groups for the change from baseline to 12 weeks.

The lipid measure closest to statistical significance was LDL cholesterol, where there was little change in the placebo group and a decrease difference in the active group. The baseline-adjusted 12 week values were 0.47 mmol/L lower in the active group.

Table 17: Change in Lipid measurements in subjects with baseline TC between 5.0-5.9 mmol/L from 12 weeks to 16 weeks

Outcome	Group	12 weeks Mean (SD)	16 weeks Mean (SD)	Change Mean (SD) [range]	% Change Mean (SD) [range]	Group Difference (*) Mean (95% CI)	P-value
TC	Placebo	5.52 (0.64)	5.32 (0.73)	-0.20 (0.64) [-1, 0.6]	-3.2 (11.7) [-16.4, 12.8]	0	
	Active	5.25 (0.54)	5.31 (0.71)	0.06 (0.52) [-0.6, 0.7]	1.3 (10.0) [-10.5, 13.3]	0.19 (-0.43, 0.82)	0.52
HDL	Placebo	1.31 (0.33)	1.29 (0.31)	-0.02 (0.12) [-0.2, 0.2]	-0.6 (11.4) [-13.3, 25]	0	
	Active	1.51 (0.49)	1.51 (0.45)	0.00 (0.15) [-0.3, 0.2]	1.5 (10.5) [-14.3, 22.2]	0.05 (-0.09, 0.19)	0.45
LDL	Placebo	3.63 (0.54)	3.47 (0.53)	-0.17 (0.52) [-0.8, 0.7]	-3.7 (14.6) [-20.5, 23.3]	0	
	Active	3.16 (0.58)	3.29 (0.57)	0.12 (0.48) [-0.6, 0.9]	5.6 (18.5) [-15.8, 42.9]	0.10 (-0.43, 0.62)	0.70
Trigs.	Placebo	1.29 (0.48)	1.24 (0.37)	-0.04 (0.44) [-0.7, 0.5]	2.5 (33.0) [-45.5, 44.4]	0	
	Active	1.26 (0.92)	1.10 (0.56)	-0.16 (0.40) [-0.8, 0.4]	-0.7 (30.0) [-29.2, 66.7]	-0.13 (-0.41, 0.15)	0.34

(*) Calculated from ANCOVA analysis, adjusting for 12 week value

There was no evidence of a difference in lipid measurements between the two groups for the period for 12 to 16 weeks.

f) Lipid outcomes - Baseline TC 6.0 + mmol/L

Similar analyses were also performed for the subgroup who had a baseline total cholesterol of 6.0 mmol/L or higher. There were 6 subjects in this subgroup, 3 in the control group, and 3 in the intervention group.

Analyses were performed to examine how the changes in lipid measurements over the course of the study varied between the two study groups. The changes between four pairs of timepoints were examined, with results summarised in Tables 18-21.

Table 18: Change in Lipid measurements in patients with baseline **TC of ≥6.0 mmol/L from baseline to 6 weeks**

Outcome	Group	Baseline Mean (SD)	6 weeks Mean (SD)	Change Mean (SD) [range]	% Change Mean (SD) [range]	Group Difference (*) Mean (95% CI)	P-value
TC	Placebo	7.10 (0.40)	7.40 (0.46)	0.30 (0.78) [-0.2, 1.2]	4.6 (11.5) [-2.7, 17.9]	0	
	Active	6.20 (0.26)	5.70 (0.53)	-0.50 (0.70) [-1, 0.3]	-7.8 (11.2) [-15.4, 5]	-2.44 (-4.77, -0.11)	**0.045**
HDL	Placebo	1.60 (0.2)	1.67 (0.15)	0.07 (0.06) [0.0, 0.1]	4.5 (3.9) [0.0, 7.1]	0	
	Active	1.40 (0.2)	1.33 (0.40)	-0.07 (0.21) [-0.3, 0.1]	-6.2 (16.5) [-25.0, 6.3]	-0.06 (-0.53, 0.41)	0.72
LDL	Placebo	4.97 (0.32)	5.13 (0.15)	0.17 (0.46) [-0.1, 0.7]	3.8 (9.9) [-2, 15.2]	0	
	Active	4.27 (0.64)	3.87 (0.49)	-0.40 (0.70) [-0.9, 0.4]	-8.3 (16.3) [-18.0, 10.5]	-1.21 (-2.64, 0.21)	0.07
Trigs.	Placebo	1.13 (0.42)	1.37 (0.99)	0.23 (0.58) [-0.1, 0.9]	11.3 (39) [-12.5, 56.3]	0	
	Active	1.20 (0.56)	1.07 (0.40)	-0.13 (0.38) [-0.4, 0.3]	-3.4 (41.2) [-36.4, 42.9]	-0.38 (-1.82, 1.07)	0.47

(*) Calculated from ANCOVA analysis, adjusting for baseline value

There was a significant difference between groups for total cholesterol. After adjusting for baseline differences, the values were significantly lower in the active group, on average by 2.4 mmol/L.

Table 19: Change in Lipid measurements in subjects with baseline TC of ≥6.0 mmol/L from 6 weeks to 12 weeks

Outcome	Group	6 weeks Mean (SD)	12 wks Mean (SD)	Change Mean (SD) [range]	% Change Mean (SD) [range]	Group Difference (*) Mean (95% CI)	P-value
TC	Placebo	7.40 (0.46)	6.73 (0.71)	-0.67 (0.78) [-1.3, 0.2]	-8.9 (10.2) [-16.5, 2.7]	0	
	Active	5.70 (0.53)	6.83 (0.47)	1.13 (0.32) [0.9, 1.5]	20.1 (6.6) [14.3, 27.3]	0.98 (-2.82, 4.79)	0.47
HDL	Placebo	1.67 (0.15)	1.50 (0.17)	-0.17 (0.12) [-0.3, -0.1]	-10.0 (6.7) [-17.6, -5.6]	0	
	Active	1.33 (0.4)	1.4 (0.3)	0.07 (0.12) [0.0, 0.2]	7.4 (12.8) [0, 22.2]	0.15 (-0.16, 0.46)	0.22
LDL	Placebo	5.13 (0.15)	4.70 (0.56)	-0.43 (0.55) [-0.8, 0.2]	-8.4 (10.8) [-16, 3.9]	0	
	Active	3.87 (0.49)	4.70 (0.56)	0.83 (0.25) [0.6, 1.1]	21.8 (6.6) [14.3, 26.8]	1.24 (-1.78, 4.25)	0.28
Trigs.	Placebo	1.37 (0.99)	1.13 (0.15)	-0.23 (0.85) [-1.2, 0.4]	6.8 (52.7) [-48, 57.1]	0	
	Active	1.07 (0.4)	1.60 (0.66)	0.53 (0.25) [0.3, 0.8]	48.7 (5.4) [42.9, 53.3]	0.57 (-0.65, 1.79)	0.23

(*) Calculated from ANCOVA analysis, adjusting for 6 week value

There was no strong evidence of any group difference for the lipid variables for the period from 6 to 12 weeks. Although not statistically significant, both total and LDL cholesterol values were higher in the active group than in the placebo group.

Table 20: Change in Lipid measurements in subjects with baseline TC of ≥6.0 mmol/L from baseline to 12 weeks

Outcome	Group	Baseline Mean (SD)	12 weeks Mean (SD)	Change Mean (SD) [range]	% Change Mean (SD) [range]	Group Difference (*) Mean (95% CI)	P-value
TC	Placebo	7.10 (0.40)	6.73 (0.71)	-0.37 (0.55) [-1, 0]	-5.2 (7.7) [-14.1, 0.0]	0	
	Active	6.20 (0.26)	6.83 (0.47)	0.63 (0.51) [0.2, 1.2]	10.3 (8.7) [3.3, 20.0]	0.86 (-2.17, 3.90)	0.43
HDL	Placebo	1.60 (0.20)	1.50 (0.17)	-0.10 (0.1) [-0.2, 0.0]	-6.0 (6.3) [-12.5, 0]	0	
	Active	1.40 (0.20)	1.40 (0.30)	0.00 (0.1) [-0.1, 0.1]	-0.7 (7.3) [-8.3, 6.3]	0.13 (-0.22, 0.47)	0.33
LDL	Placebo	4.97 (0.32)	4.70 (0.56)	-0.27 (0.55) [-0.9, 0.1]	-5.2 (10.8) [-17.6, 1.9]	0	
	Active	4.27 (0.64)	4.70 (0.56)	0.43 (0.49) [0.1, 1.0]	10.9 (13.3) [2.5, 26.3]	0.40 (-1.47, 2.26)	0.54
Trigs.	Placebo	1.13 (0.42)	1.13 (0.15)	0.00 (0.30) [-0.3, 0.3]	6.3 (28.6) [-18.7, 37.5]	0	
	Active	1.20 (0.56)	1.60 (0.66)	0.40 (0.46) [-0.1, 0.8]	44.3 (63.3) [-9.1, 114.3]	0.43 (-0.63, 1.47)	0.29

(*) Calculated from ANCOVA analysis, adjusting for baseline value

There were no statistically significant differences between the two study groups for the change from baseline to 12 weeks.

Table 21: Change in Lipid measurements in subjects with baseline TC of ≥6.0 mmol/L from 12 weeks to 16 weeks

Outcome	Group	12 weeks Mean (SD)	16 weeks Mean (SD)	Change Mean (SD) [range]	% Change Mean (SD) [range]	Group Difference (*) Mean (95% CI)	P-value
TC	Placebo	6.73 (0.71)	6.80 (1.04)	0.07 (0.40) [-0.3, 0.5]	0.7 (5.6) [-4.5, 6.7]	0	
	Active	6.75 (0.64)	6.00 (0.28)	-0.75 (0.35) [-1, -0.5]	-10.9 (4.2) [-13.9, -7.9]	-0.82 (-2.63, 0.99)	0.19
HDL	Placebo	1.50 (0.17)	1.47 (0.12)	-0.03 (0.06) [-0.1, 0.0]	-2 (3.4) [-5.9, 0.0]	0	
	Active	1.55 (0.21)	1.50 (0.42)	-0.05 (0.21) [-0.2, 0.1]	-4.2 (14.3) [-14.3, 5.9]	-0.03 (-0.63, 0.57)	0.86
LDL	Placebo	4.70 (0.56)	4.57 (1.03)	-0.13 (0.68) [-0.9, 0.4]	-3.2 (14.4) [-19.6, 7.5]	0	
	Active	4.45 (0.49)	3.80 (0.28)	-0.65 (0.21) [-0.8, -0.5]	-14.4 (3.2) [-16.7, -12.2]	-0.47 (-3.27, 2.33)	0.55
Trigs.	Placebo	1.13 (0.15)	1.70 (0.92)	0.57 (0.80) [-0.2, 1.4]	46.5 (63.0) [-18.2, 107.7]	0	
	Active	1.65 (0.92)	1.55 (0.92)	-0.10 (0.00) [-0.1, -0.1]	-7.2 (4.0) [-10.0, -4.3]	-0.77 (-4.39, 2.85)	0.46

(*) Calculated from ANCOVA analysis, adjusting for 12 week value

There were no statistically significant differences in lipid measurements between the two groups for the period for 12 to 16 weeks. Although not significant, the total cholesterol values were lower in the active group.

g) Lipid outcomes - Females

The next set of analyses were restricted to females only. There were 32 females in the study, 14 in the control group, and 18 in the intervention group.

Analyses were performed to examine how the changes in lipid measurements over the course of the study varied between the two study groups. The changes between four pairs of timepoints were examined, with results summarised in Tables 22-25.

Table 22: Change in Lipid measurements in females from baseline to 6 weeks

Outcome	Group	Baseline Mean (SD)	6 weeks Mean (SD)	Change Mean (SD) [range]	% Change Mean (SD) [range]	Group Difference (*) Mean (95% CI)	P-value
TC	Placebo	5.27 (0.89)	5.38 (1.08)	0.11 (0.71) [-0.8, 1.2]	2.5 (14.2) [-14.8, 25.6]	0	
	Active	5.07 (0.67)	5.02 (0.68)	-0.05 (0.45) [-0.9, 0.7]	-0.6 (9.0) [-15.8, 16.7]	-0.18 (-0.61, 0.34)	0.38
HDL	Placebo	1.31 (0.28)	1.37 (0.28)	0.06 (0.17) [-0.2, 0.4]	5.7 (16.1) [-15.4, 50.0]	0	
	Active	1.47 (0.36)	1.53 (0.41)	0.07 (0.16) [-0.2, 0.3]	4.5 (11.0) [-13.3, 22.2]	0.01 (-0.11, 0.13)	0.85
LDL	Placebo	3.45 (0.78)	3.53 (0.83)	0.08 (0.54) [-0.7, 0.9]	3.6 (18.4) [-18.4, 37.5]	0	
	Active	3.15 (0.60)	3.05 (0.61)	-0.10 (0.49) [-1.6, 0.6]	-2.3 (14.0) [-40.0, 20.7]	-0.25 (-0.62, 0.12)	0.18
Trigs.	Placebo	1.11 (0.41)	1.07 (0.48)	-0.04 (0.34) [-0.6, 0.9]	-1.9 (22.7) [-35.3, 56.3]	0	
	Active	1.02 (0.33)	0.95 (0.54)	-0.07 (0.40) [-0.6, 1.1]	-6.9 (32.4) [-50.0, 64.7]	-0.03 (-0.31, 0.25)	0.84

(*) Calculated from ANCOVA analysis, adjusting for baseline value

There were no statistically significant difference between groups for any of the lipid measures.

Table 23: Change in Lipid measurements in females from 6 weeks to 12 weeks

Outcome	Group	6 weeks Mean (SD)	12 wks Mean (SD)	Change Mean (SD) [range]	% Change Mean (SD) [range]	Group Difference (*) Mean (95% CI)	P-value
TC	Placebo	5.38 (1.08)	5.49 (0.70)	0.11 (0.77) [-1.3, 1.5]	4.0 (14.3) [-16.7, 32.6]	0	
	Active	5.02 (0.68)	5.07 (0.82)	0.04 (0.58) [-1.3, 1.0]	1.2 (10.6) [-22.4, 18.9]	-0.21 (-0.64, 0.21)	0.32
HDL	Placebo	1.37 (0.28)	1.31 (0.26)	-0.06 (0.21) [-0.3, 0.4]	-3.1 (15.7) [-21.4, 36.4]	0	
	Active	1.53 (0.41)	1.55 (0.42)	0.02 (0.13) [-0.2, 0.2]	1.0 (9.2) [-18.2, 15.4]	0.09 (-0.03, 0.21)	0.15
LDL	Placebo	3.53 (0.83)	3.63 (0.64)	0.10 (0.60) [-0.8, 1.1]	5.2 (18.3) [-21.2, 35.5]	0	
	Active	3.05 (0.61)	3.05 (0.70)	0.00 (0.46) [-1.1, 0.8]	0.5 (13.9) [-34.4, 24.2]	-0.26 (-0.63, 0.12)	0.17
Trigs.	Placebo	1.07 (0.48)	1.21 (0.45)	0.14 (0.51) [-1.2, 1.0]	20.1 (36.4) [-48.0, 83.3]	0	
	Active	0.95 (0.54)	1.03 (0.54)	0.08 (0.27) [-0.4, 0.8]	11.2 (26.6) [-33.3, 57.1]	-0.10 (-0.36, 0.17)	0.46

(*) Calculated from ANCOVA analysis, adjusting for 6 week value

Again, no differences were statistically significant.

Table 24: Change in Lipid measurements in females from baseline to 12 weeks

Outcome	Group	Baseline Mean (SD)	12 weeks Mean (SD)	Change Mean (SD) [range]	% Change Mean (SD) [range]	Group Difference (*) Mean (95% CI)	P-value
TC	Placebo	5.27 (0.89)	5.49 (0.70)	0.22 (0.71) [-1.0, 1.4]	5.6 (13.9) [-14.1, 34.1]	0	
	Active	5.07 (0.67)	5.07 (0.82)	-0.01 (0.62) [-1.3, 1.2]	0.2 (11.7) [-22.4, 23.3]	-0.30 (-0.75, 0.14)	0.18
HDL	Placebo	1.31 (0.28)	1.31 (0.26)	0.00 (0.19) [-0.2, 0.5]	1.7 (19.5) [-12.5, 62.5]	0	
	Active	1.47 (0.36)	1.55 (0.42)	0.08 (0.16) [-0.1, 0.5]	5.1 (10.8) [-12.5, 33.3]	0.09 (-0.04, 0.22)	0.18
LDL	Placebo	3.45 (0.78)	3.63 (0.64)	0.18 (0.66) [-0.9, 1.3]	7.8 (20.5) [-21.1, 52.0]	0	
	Active	3.15 (0.60)	3.05 (0.70)	-0.10 (0.59) [-1.3, 1]	-2.3 (17.4) [-36.4, 26.3]	-0.41 (-0.82, 0.01)	0.06
Trigs.	Placebo	1.11 (0.41)	1.21 (0.45)	0.09 (0.38) [-0.9, 0.6]	14.7 (34.0) [-52.9, 83.3]	0	
	Active	1.02 (0.33)	1.03 (0.54)	0.01 (0.31) [-0.5, 0.7]	-0.6 (26.6) [-55.6, 41.2]	-0.08 (-0.34, 0.17)	0.51

(*) Calculated from ANCOVA analysis, adjusting for baseline value

There was no statistically significant differences in Lipid measurements in females from baseline to 12 weeks

Table 25: Change in Lipid measurements in females from 12 weeks to 16 weeks

Outcome	Group	12 weeks Mean (SD)	16 weeks Mean (SD)	Change Mean (SD) [range]	% Change Mean (SD) [range]	Group Difference (*) Mean (95% CI)	P-value
TC	Placebo	5.49 (0.70)	5.17 (0.85)	-0.32 (0.61) [-1.1, 0.8]	-5.6 (11.8) [-20, 17.8]	0	
	Active	5.07 (0.82)	5.08 (0.72)	0.01 (0.53) [-1.0, 0.8]	0.9 (10) [-13.9, 16.0]	0.22 (-0.19, 0.63)	0.28
HDL	Placebo	1.31 (0.26)	1.3 (0.26)	-0.01 (0.12) [-0.2, 0.2]	-0.4 (11.3) [-15.4, 25]	0	
	Active	1.55 (0.42)	1.52 (0.39)	-0.03 (0.17) [-0.4, 0.2]	-0.3 (11.2) [-20, 22.2]	0.02 (-0.09, 0.13)	0.68
LDL	Placebo	3.63 (0.64)	3.32 (0.60)	-0.31 (0.53) [-0.9, 0.7]	-7.4 (15.1) [-22.9, 23.3]	0	
	Active	3.05 (0.70)	3.11 (0.59)	0.06 (0.47) [-0.8, 0.9]	3.6 (15.9) [-16.7, 42.9]	0.14 (-0.20, 0.49)	0.40
Trigs.	Placebo	1.21 (0.45)	1.22 (0.60)	0.01 (0.53) [-0.7, 1.4]	3.6 (42.4) [-55.6, 107.7]	0	
	Active	1.03 (0.54)	0.99 (0.45)	-0.03 (0.27) [-0.7, 0.4]	2.7 (31.6) [-44.4, 75.0]	-0.10 (-0.38, 0.19)	0.48

(*) Calculated from ANCOVA analysis, adjusting for 12 week value

There were no statistically significant differences in lipid measurements between the two groups for the period for 12 to 16 weeks.

h) Lipid outcomes - Males

The next set of analyses were restricted to males only. There were 14 males in the study, 9 in the control group, and 5 in the intervention group.

Analyses were performed to examine how the changes in lipid measurements over the course of the study varied between the two study groups. The changes between four pairs of timepoints were examined, with results summarised in Tables 26-29.

Table 26: Change in Lipid measurements in males from baseline to 6 weeks

Outcome	Group	Baseline Mean (SD)	6 weeks Mean (SD)	Change Mean (SD) [range]	% Change Mean (SD) [range]	Group Difference (*) Mean (95% CI)	P-value
TC	Placebo	5.14 (1.01)	5.24 (0.89)	0.10 (0.59) [-0.8, 1.0]	2.9 (12.3) [-14.0, 23.3]	0	
	Active	5.20 (0.90)	4.96 (0.65)	-0.24 (0.54) [-1, 0.4]	-3.7 (9.8) [-15.4, 9.5]	-0.32 (-0.94, 0.29)	0.27
HDL	Placebo	1.15 (0.35)	1.23 (0.33)	0.08 (0.10) [0.0, 0.2]	7.7 (11.0) [0.0, 25.0]	0	
	Active	1.18 (0.22)	1.14 (0.29)	-0.04 (0.17) [-0.3, 0.1]	-3.5 (13.8) [-25.0, 9.1]	-0.11 (-0.29, 0.06)	0.17
LDL	Placebo	3.41 (0.81)	3.42 (0.84)	0.01 (0.47) [-0.6, 0.7]	1.1 (15.1) [-17.1, 23.3]	0	
	Active	3.40 (1.00)	3.10 (0.64)	-0.30 (0.49) [-0.9, 0.4]	-6.4 (14.8) [-18.7, 17.4]	-0.32 (-0.87, 0.24)	0.24
Trigs.	Placebo	1.34 (0.51)	1.24 (0.49)	-0.10 (0.43) [-0.9, 0.4]	-1.7 (32.1) [-52.9, 50.0]	0	
	Active	1.44 (0.72)	1.54 (0.92)	0.10 (0.32) [-0.3, 0.5]	5.5 (29.0) [-33.3, 42.9]	0.21 (-0.32, 0.73)	0.40

(*) Calculated from ANCOVA analysis, adjusting for baseline value

There was no evidence of a significant difference between groups for any of the four measures.

Table 27: Change in Lipid measurements in males from 6 weeks to 12 weeks

Outcome	Group	6 weeks Mean (SD)	12 wks Mean (SD)	Change Mean (SD) [range]	% Change Mean (SD) [range]	Group Difference (*) Mean (95% CI)	P-value
TC	Placebo	5.24 (0.89)	5.08 (1.01)	-0.17 (0.63) [-1.4, 0.5]	-2.9 (11.8) [-25.5, 10.2]	0	
	Active	4.96 (0.65)	5.32 (1.11)	0.36 (0.67) [-0.2, 1.5]	6.8 (12.5) [-4.3, 27.3]	0.53 (-0.31, 1.37)	0.19
HDL	Placebo	1.23 (0.33)	1.16 (0.32)	-0.06 (0.07) [-0.2, 0.0]	-4.9 (5.7) [-14.3, 0.0]	0	
	Active	1.14 (0.29)	1.14 (0.18)	0.00 (0.14) [-0.2, 0.2]	1.9 (12.6) [-12.5, 22.2]	0.05 (-0.07, 0.16)	0.39
LDL	Placebo	3.42 (0.84)	3.49 (0.83)	0.06 (0.42) [-0.6, 0.6]	2.8 (12.9) [-16.2, 20.7]	0	
	Active	3.10 (0.64)	3.44 (1.03)	0.34 (0.48) [-0.1, 1.1]	10.0 (12.9) [-3.7, 26.8]	0.28 (-0.32, 0.89)	0.32
Trigs.	Placebo	1.24 (0.49)	1.19 (0.34)	-0.05 (0.28) [-0.6, 0.3]	0.9 (19.6) [-30.0, 30.0]	0	
	Active	1.54 (0.92)	1.60 (0.89)	0.06 (0.36) [-0.4, 0.5]	10.8 (30.6) [-18.2, 50.0]	0.18 (-0.20, 0.55)	0.32

(*) Calculated from ANCOVA analysis, adjusting for 6 week value

There were no evidence of any group difference for the lipid variables for the period from 6 to 12 weeks.

Table 28: Change in Lipid measurements in males from baseline to 12 weeks

Outcome	Group	Baseline Mean (SD)	12 weeks Mean (SD)	Change Mean (SD) [range]	% Change Mean (SD) [range]	Group Difference (*) Mean (95% CI)	P-value
TC	Placebo	5.14 (1.01)	5.08 (1.01)	-0.07 (0.58) [-0.8, 0.8]	-0.7 (12.3) [-14.6, 17.0]	0	
	Active	5.2 (0.90)	5.32 (1.11)	0.12 (0.28) [-0.2, 0.5]	1.9 (4.9) [-4.2, 7.7]	0.19 (-0.45, 0.83)	0.53
HDL	Placebo	1.12 (0.33)	1.12 (0.32)	0.00 (0.14) [-0.2, 0.2]	1.0 (13.2) [-14.3, 25.0]	0	
	Active	1.18 (0.22)	1.14 (0.18)	-0.04 (0.09) [-0.1, 0.1]	-2.8 (7.5) [-8.3, 9.1]	-0.03 (-0.18, 0.12)	0.66
LDL	Placebo	3.42 (0.76)	3.40 (0.82)	-0.02 (0.55) [-0.8, 0.9]	0.4 (18.1) [-22.9, 34.6]	0	
	Active	3.4 (1.00)	3.44 (1.03)	0.04 (0.19) [-0.1, 0.3]	1.5 (7.1) [-3.3, 13.0]	0.06 (-0.53, 0.64)	0.83
Trigs.	Placebo	1.28 (0.51)	1.20 (0.32)	-0.08 (0.44) [-0.9, 0.5]	5.0 (39.4) [-52.9, 62.5]	0	
	Active	1.44 (0.72)	1.60 (0.89)	0.16 (0.55) [-0.3, 0.8]	18.9 (57.4) [-25.0, 114.3]	0.30 (-0.26, 0.85)	0.27

(*) Calculated from ANCOVA analysis, adjusting for baseline value

There were no statistically significant differences between the two study groups for the change from baseline to 12 weeks.

Table 29: Change in Lipid measurements in males from 12 weeks to 16 weeks

Outcome	Group	12 weeks Mean (SD)	16 weeks Mean (SD)	Change Mean (SD) [range]	% Change Mean (SD) [range]	Group Difference (*) Mean (95% CI)	P-value
TC	Placebo	5.08 (1.01)	5.23 (1.12)	0.16 (0.33) [-0.3, 0.6]	3.1 (6.6) [-6.4, 12.8]	0	
	Active	4.90 (0.68)	4.88 (0.51)	-0.03 (0.34) [-0.3, 0.4]	-0.1 (7.2) [-6.4, 9.1]	-0.18 (-0.65, 0.29)	0.41
HDL	Placebo	1.12 (0.32)	1.13 (0.31)	0.01 (0.13) [-0.1, 0.2]	1.5 (11.8) [-12.5, 20]	0	
	Active	1.15 (0.21)	1.17 (0.33)	0.03 (0.13) [-0.1, 0.2]	0.8 (10.4) [-11.1, 14.3]	0.01 (-0.16, 0.19)	0.86
LDL	Placebo	3.40 (0.82)	3.53 (0.86)	0.13 (0.23) [-0.3, 0.4]	4.2 (7.1) [-8.6, 14.8]	0	
	Active	3.00 (0.34)	3.00 (0.34)	0.00 (0.26) [-0.3, 0.3]	0.3 (9.1) [-9.7, 11.5]	-0.13 (-0.49, 0.22)	0.41
Trigs.	Placebo	1.20 (0.32)	1.22 (0.34)	0.02 (0.34) [-0.4, 0.5]	5.4 (29.3) [-30.8, 50]	0	
	Active	1.63 (1.02)	1.52 (0.90)	-0.10 (0.57) [-0.8, 0.6]	-4.2 (26) [-26.7, 33.3]	0.01 (-0.54, 0.57)	0.96

(*) Calculated from ANCOVA analysis, adjusting for 12 week value

There were no statistically significant differences in lipid measurements between the two groups for the period for 12 to 16 weeks.

i) Lipid outcomes - Age <50

Analyses were performed for the subgroup of subjects aged less than 50. There were 16 subjects in this age group in the study, 7 in the control group, and 9 in the intervention group.

Analyses were performed to examine how the changes in lipid measurements over the course of the study varied between the two study groups. The changes between four pairs of timepoints were examined, with results summarised in Tables 30-33.

Table 30: Change in Lipid measurements in subjects aged less than 50 from baseline to 6 weeks

Outcome	Group	Baseline Mean (SD)	6 weeks Mean (SD)	Change Mean (SD) [range]	% Change Mean (SD) [range]	Group Difference (*) Mean (95% CI)	P-value
TC	Placebo	4.89 (0.58)	5.00 (0.55)	0.11 (0.69) [-0.8, 1.0]	3.3 (14.5) [-14.8, 23.3]	0	
	Active	5.21 (0.86)	5.07 (0.92)	-0.14 (0.50) [-1, 0.5]	-2.7 (9.4) [-15.4, 11.6]	-0.17 (-0.82, 0.47)	0.57
HDL	Placebo	1.15 (0.34)	1.20 (0.23)	0.05 (0.14) [-0.1, 0.2]	7.5 (14.2) [-7.7, 25]	0	
	Active	1.29 (0.25)	1.29 (0.39)	0.00 (0.21) [-0.3, 0.3]	-1 (14.7) [-25, 20]	-0.05 (-0.27, 0.18)	0.67
LDL	Placebo	3.27 (0.56)	3.30 (0.65)	0.03 (0.57) [-0.7, 0.7]	1.8 (17.9) [-18.4, 23.3]	0	
	Active	3.42 (0.88)	3.23 (0.82)	-0.19 (0.42) [-0.9, 0.4]	-4.9 (11.8) [-18.7, 13]	-0.19 (-0.74, 0.36)	0.47
Trigs.	Placebo	1.07 (0.43)	0.95 (0.23)	-0.12 (0.34) [-0.5, 0.4]	-2.8 (31.9) [-33.3, 50]	0	
	Active	1.14 (0.56)	1.17 (0.68)	0.02 (0.28) [-0.3, 0.5]	2.2 (24.4) [-25, 42.9]	0.15 (-0.21, 0.51)	0.39

(*) Calculated from ANCOVA analysis, adjusting for baseline value

There was no evidence of a significant difference between groups for any of the four measures.

Table 31: Change in Lipid measurements in subjects aged less than 50 from 6 weeks to 12 weeks

Outcome	Group	6 weeks Mean (SD)	12 wks Mean (SD)	Change Mean (SD) [range]	% Change Mean (SD) [range]	Group Difference (*) Mean (95% CI)	P-value
TC	Placebo	5.00 (0.55)	4.93 (0.53)	-0.07 (0.7) [-1.4, 0.7]	-0.5 (13.7) [-25.5, 16.7]	0	
	Active	5.07 (0.92)	5.39 (1.15)	0.32 (0.62) [-0.6, 1.5]	6.4 (11) [-10.5, 27.3]	0.40 (-0.32, 1.13)	0.26
HDL	Placebo	1.20 (0.23)	1.15 (0.28)	-0.05 (0.08) [-0.2, 0.0]	-4.8 (8.3) [-20, 0]	0	
	Active	1.29 (0.39)	1.36 (0.38)	0.07 (0.11) [-0.1, 0.2]	6.0 (9.1) [-5.0, 22.2]	0.12 (0.00, 0.24)	0.06
LDL	Placebo	3.30 (0.65)	3.33 (0.48)	0.03 (0.39) [-0.6, 0.6]	2.7 (13.9) [-16.2, 26.1]	0	
	Active	3.23 (0.82)	3.42 (1.01)	0.19 (0.50) [-0.6, 1.1]	5.8 (13.2) [-14.0, 26.8]	0.15 (-0.40, 0.70)	0.56
Trigs.	Placebo	0.95 (0.23)	1.28 (0.50)	0.33 (0.34) [0.1, 1.0]	33.0 (26.5) [8.3, 83.3]	0	
	Active	1.17 (0.68)	1.36 (0.79)	0.19 (0.31) [-0.2, 0.8]	17.2 (26.7) [-18.2, 53.3]	-0.17 (-0.55, 0.21)	0.34

(*) Calculated from ANCOVA analysis, adjusting for 6 week value

There were no statistically significant differences in in Lipid measurements in subjects aged less than 50 from 6 weeks to 12 weeks

Table 32: Change in Lipid measurements in subjects aged less than 50 from baseline to 12 weeks

Outcome	Group	Baseline Mean (SD)	12 weeks Mean (SD)	Change Mean (SD) [range]	% Change Mean (SD) [range]	Group Difference (*) Mean (95% CI)	P-value
TC	Placebo	4.89 (0.58)	4.93 (0.53)	0.04 (0.57) [-0.7, 0.8]	1.6 (11.9) [-14.6, 17]	0	
	Active	5.21 (0.86)	5.39 (1.15)	0.18 (0.61) [-0.5, 1.2]	3.2 (11.9) [-10.9, 23.3]	0.15 (-0.54, 0.83)	0.65
HDL	Placebo	1.11 (0.33)	1.10 (0.29)	-0.01 (0.12) [-0.1, 0.2]	0 (13.5) [-11.1, 25.0]	0	
	Active	1.29 (0.25)	1.36 (0.38)	0.07 (0.19) [-0.1, 0.5]	4.2 (12.8) [-8.3, 33.3]	0.07 (-0.12, 0.26)	0.44
LDL	Placebo	3.30 (0.52)	3.24 (0.50)	-0.06 (0.57) [-0.8, 0.7]	-0.4 (16.8) [-22.9, 21.9]	0	
	Active	3.42 (0.88)	3.42 (1.01)	0.00 (0.54) [-0.6, 1.0]	0.4 (16.5) [-21.4, 26.3]	0.08 (-0.53, 0.69)	0.79
Trigs.	Placebo	1.03 (0.40)	1.29 (0.45)	0.26 (0.3) [-0.2, 0.6]	30.5 (30.5) [-13.3, 62.5]	0	
	Active	1.14 (0.56)	1.36 (0.79)	0.21 (0.4) [-0.3, 0.8]	20.3 (42.7) [-25.0, 114.3]	-0.06 (-0.47, 0.35)	0.75

(*) Calculated from ANCOVA analysis, adjusting for baseline value

There were no statistically significant differences between the two study groups for the change from baseline to 12 weeks.

Table 33: Change in Lipid measurements in subjects aged less than 50 from 12 weeks to 16 weeks

Outcome	Group	12 weeks Mean (SD)	16 weeks Mean (SD)	Change Mean (SD) [range]	% Change Mean (SD) [range]	Group Difference (*) Mean (95% CI)	P-value
TC	Placebo	4.93 (0.53)	4.81 (0.53)	-0.11 (0.45) [-0.7, 0.6]	-2.0 (9.1) [-14.6, 12.5]	0	
	Active	5.19 (1.05)	5.07 (0.81)	-0.11 (0.54) [-1.0, 0.7]	-1.2 (9.4) [-13.9, 13.2]	0.09 (-0.40, 0.57)	0.70
HDL	Placebo	1.10 (0.29)	1.09 (0.29)	-0.01 (0.11) [-0.1, 0.2]	-0.8 (12.6) [-12.5, 25]	0	
	Active	1.39 (0.39)	1.35 (0.35)	-0.04 (0.18) [-0.4, 0.1]	-1.7 (10.3) [-20.0, 9.1]	0.02 (-0.16, 0.20)	0.80
LDL	Placebo	3.24 (0.5)	3.24 (0.44)	0.00 (0.44) [-0.5, 0.7]	1.0 (14) [-16.1, 23.3]	0	
	Active	3.20 (0.81)	3.16 (0.63)	-0.04 (0.42) [-0.8, 0.4]	0.4 (12.1) [-16.7, 13.6]	-0.05 (-0.46, 0.35)	0.78
Trigs.	Placebo	1.29 (0.45)	1.07 (0.36)	-0.21 (0.36) [-0.7, 0.3]	-13.5 (31.4) [-55.6, 37.5]	0	
	Active	1.34 (0.85)	1.21 (0.65)	-0.12 (0.40) [-0.8, 0.4]	-1.8 (37.8) [-44.4, 66.7]	0.11 (-0.24, 0.45)	0.51

(*) Calculated from ANCOVA analysis, adjusting for 12 week value

There were no statistically significant differences in lipid measurements between the two groups for the period for 12 to 16 weeks.

j) Lipid outcomes - Age 50-59

Analyses were performed for the subgroup of subjects aged 50-59. There were 18 subjects in this age group in the study, 12 in the control group, and 6 in the intervention group.

Analyses were performed to examine how the changes in lipid measurements over the course of the study varied between the two study groups. The changes between four pairs of timepoints were examined, with results summarised in Tables 34-37.

Table 34: Change in Lipid measurements in subjects aged 50-59 from baseline to 6 weeks

Outcome	Group	Baseline Mean (SD)	6 weeks Mean (SD)	Change Mean (SD) [range]	% Change Mean (SD) [range]	Group Difference (*) Mean (95% CI)	P-value
TC	Placebo	5.28 (1.04)	5.37 (1.16)	0.09 (0.71) [-0.8, 1.2]	2.3 (14.1) [-14, 25.6]	0	
	Active	4.95 (0.53)	5.03 (0.23)	0.08 (0.55) [-0.9, 0.7]	2.6 (11.0) [-15.8, 16.7]	-0.07 (-0.78, 0.64)	0.84
HDL	Placebo	1.22 (0.30)	1.27 (0.30)	0.05 (0.16) [-0.2, 0.4]	5.4 (16.5) [-15.4, 50]	0	
	Active	1.30 (0.38)	1.37 (0.37)	0.07 (0.10) [0.0, 0.2]	5.9 (9.6) [0.0, 22.2]	0.03 (-0.13, 0.18)	0.73
LDL	Placebo	3.41 (0.85)	3.48 (0.89)	0.07 (0.54) [-0.6, 0.9]	3.7 (18.9) [-17.1, 37.5]	0	
	Active	3.13 (0.48)	3.08 (0.41)	-0.05 (0.78) [-1.6, 0.6]	0.8 (21.1) [-40.0, 20.7]	-0.21 (-0.86, 0.43)	0.49
Trigs.	Placebo	1.42 (0.40)	1.35 (0.54)	-0.07 (0.44) [-0.9, 0.9]	-3.7 (26.9) [-52.9, 56.3]	0	
	Active	1.12 (0.37)	1.27 (0.80)	0.15 (0.59) [-0.6, 1.1]	11.6 (44.5) [-46.2, 64.7]	0.23 (-0.36, 0.81)	0.42

(*) Calculated from ANCOVA analysis, adjusting for baseline value

There was no evidence of a significant difference between groups for any of the four measures.

Table 35: Change in Lipid measurements in subjects aged 50-59 from 6 weeks to 12 weeks

Outcome	Group	6 weeks Mean (SD)	12 wks Mean (SD)	Change Mean (SD) [range]	% Change Mean (SD) [range]	Group Difference (*) Mean (95% CI)	P-value
TC	Placebo	5.37 (1.16)	5.50 (0.99)	0.13 (0.81) [-1.3, 1.5]	4.1 (15.3) [-16.7, 32.6]	0	
	Active	5.03 (0.23)	4.90 (0.52)	-0.13 (0.45) [-0.8, 0.5]	-2.7 (9.1) [-16.3, 9.6]	-0.39 (-1.08, 0.30)	0.25
HDL	Placebo	1.27 (0.30)	1.27 (0.32)	0.01 (0.17) [-0.3, 0.4]	1.1 (14) [-21.4, 36.4]	0	
	Active	1.37 (0.37)	1.32 (0.42)	-0.05 (0.10) [-0.2, 0.1]	-4.8 (9.2) [-18.2, 7.1]	-0.06 (-0.23, 0.12)	0.49
LDL	Placebo	3.48 (0.89)	3.67 (0.83)	0.18 (0.63) [-0.7, 1.1]	7.2 (19.2) [-21.2, 35.5]	0	
	Active	3.08 (0.41)	3.02 (0.41)	-0.07 (0.40) [-0.7, 0.4]	-1.4 (12.5) [-20, 12.5]	-0.38 (-0.96, 0.20)	0.18
Trigs.	Placebo	1.35 (0.54)	1.21 (0.42)	-0.14 (0.45) [-1.2, 0.6]	-6.0 (25.7) [-48.0, 46.2]	0	
	Active	1.27 (0.80)	1.25 (0.61)	-0.02 (0.29) [-0.4, 0.4]	6.7 (28.1) [-23.1, 57.1]	0.09 (-0.26, 0.44)	0.59

(*) Calculated from ANCOVA analysis, adjusting for 6 week value

There were no strong evidence of any group difference for the lipid variables for the period from 6 to 12 weeks.

Table 36: Change in Lipid measurements in subjects aged 50-59 from baseline to 12 weeks

Outcome	Group	Baseline Mean (SD)	12 weeks Mean (SD)	Change Mean (SD) [range]	% Change Mean (SD) [range]	Group Difference (*) Mean (95% CI)	P-value
TC	Placebo	5.28 (1.04)	5.50 (0.99)	0.22 (0.70) [-0.8, 1.4]	5.5 (14.6) [-14.5, 34.1]	0	
	Active	4.95 (0.53)	4.90 (0.52)	-0.05 (0.58) [-1, 0.6]	-0.5 (10.9) [-17.5, 11.8]	-0.38 (-1.05, 0.30)	0.25
HDL	Placebo	1.22 (0.30)	1.27 (0.32)	0.06 (0.18) [-0.1, 0.5]	6.0 (19.9) [-10, 62.5]	0	
	Active	1.30 (0.38)	1.32 (0.42)	0.02 (0.12) [-0.1, 0.2]	0.4 (9.1) [-12.5, 13.3]	-0.04 (-0.22, 0.14)	0.66
LDL	Placebo	3.41 (0.85)	3.67 (0.83)	0.26 (0.64) [-0.7, 1.3]	9.9 (21.7) [-19.4, 52.0]	0	
	Active	3.13 (0.48)	3.02 (0.41)	-0.12 (0.66) [-1.3, 0.6]	-2.0 (17.8) [-32.5, 18.8]	-0.49 (-1.12, 0.15)	0.12
Trigs.	Placebo	1.42 (0.40)	1.21 (0.42)	-0.21 (0.39) [-0.9, 0.3]	-12.5 (23.7) [-52.9, 20.0]	0	
	Active	1.12 (0.37)	1.25 (0.61)	0.13 (0.32) [-0.2, 0.7]	9.6 (21.8) [-15.4, 41.2]	0.30 (-0.13, 0.72)	0.16

(*) Calculated from ANCOVA analysis, adjusting for baseline value

There was no strong evidence of statistically significant differences between the two study groups for the change from baseline to 12 weeks.

Table 37: Change in Lipid measurements in subjects aged 50-59 from 12 weeks to 16 weeks

Outcome	Group	12 weeks Mean (SD)	16 weeks Mean (SD)	Change Mean (SD) [range]	% Change Mean (SD) [range]	Group Difference (*) Mean (95% CI)	P-value
TC	Placebo	5.5 (0.99)	5.38 (1.18)	-0.12 (0.66) [-1.1, 0.8]	-2.0 (12.6) [-20, 17.8]	0	
	Active	4.9 (0.52)	4.85 (0.52)	-0.05 (0.56) [-0.6, 0.8]	-0.5 (11.5) [-10.5, 16.0]	0.02 (-0.71, 0.76)	0.95
HDL	Placebo	1.27 (0.32)	1.25 (0.29)	-0.03 (0.14) [-0.2, 0.2]	-0.9 (11.7) [-15.4, 20.0]	0	
	Active	1.32 (0.42)	1.30 (0.30)	-0.02 (0.17) [-0.3, 0.2]	2.2 (14.3) [-17.6, 22.2]	0.02 (-0.11, 0.15)	0.76
LDL	Placebo	3.67 (0.83)	3.48 (0.88)	-0.18 (0.53) [-0.9, 0.5]	-4.2 (14.4) [-22.9, 19.2]	0	
	Active	3.02 (0.41)	3.03 (0.52)	0.02 (0.51) [-0.6, 0.8]	1.1 (16) [-15.8, 25.8]	0.09 (-0.53, 0.70)	0.77
Trigs.	Placebo	1.21 (0.42)	1.42 (0.57)	0.22 (0.49) [-0.5, 1.4]	21.0 (37.6) [-26.3, 107.7]	0	
	Active	1.25 (0.61)	1.18 (0.35)	-0.07 (0.32) [-0.7, 0.2]	0.0 (16.3) [-29.2, 20.0]	-0.27 (-0.72, 0.18)	0.22

(*) Calculated from ANCOVA analysis, adjusting for 12 week value

There were no statistically significant differences in lipid measurements between the two groups for the period for 12 to 16 weeks.

k) Lipid outcomes - Age 60+

Analyses were performed for the subgroup of subjects aged 60 and over. There were 12 subjects in this age group in the study, 4 in the control group, and 8 in the intervention group.

Analyses were performed to examine how the changes in lipid measurements over the course of the study varied between the two study groups. The changes between four pairs of timepoints were examined, with results summarised in Tables 38-41.

Table 38: Change in Lipid measurements in subjects aged 60 and over from baseline to 6 weeks

Outcome	Group	Baseline Mean (SD)	6 weeks Mean (SD)	Change Mean (SD) [range]	% Change Mean (SD) [range]	Group Difference (*) Mean (95% CI)	P-value
TC	Placebo	5.65 (1.01)	5.78 (1.02)	0.12 (0.56) [-0.4, 0.9]	2.6 (11.1) [-8.2, 18.0]	0	
	Active	5.09 (0.70)	4.93 (0.58)	-0.16 (0.37) [-0.8, 0.4]	-2.7 (7.2) [-13.1, 9.5]	-0.41 (-1.02, 0.20)	0.16
HDL	Placebo	1.52 (0.10)	1.65 (0.17)	0.13 (0.13) [0, 0.3]	8.1 (8.4) [0.0, 20.0]	0	
	Active	1.61 (0.38)	1.69 (0.42)	0.08 (0.15) [-0.2, 0.3]	4.6 (9.6) [-12.5, 21.4]	-0.05 (-0.26, 0.15)	0.57
LDL	Placebo	3.78 (0.90)	3.8 (0.91)	0.03 (0.42) [-0.4, 0.6]	1.1 (13.0) [-12.5, 18.8]	0	
	Active	3.01 (0.56)	2.85 (0.40)	-0.16 (0.32) [-0.7, 0.4]	-4.3 (10.7) [-17.5, 17.4]	-0.38 (-0.90, 0.15)	0.14
Trigs.	Placebo	0.73 (0.15)	0.75 (0.10)	0.03 (0.10) [-0.1, 0.1]	5.2 (14.2) [-12.5, 16.7]	0	
	Active	1.06 (0.45)	0.84 (0.55)	-0.23 (0.23) [-0.6, 0.1]	-23.1 (18.9) [-50.0, 4.8]	0.28 (-0.59, 0.03)	0.07

(*) Calculated from ANCOVA analysis, adjusting for baseline value

There were no statistically significant differences in lipid outcomes in the over 60 years age group between baseline and 6 weeks.

Table 39: Change in Lipid measurements in subjects aged 60 and over from 6 weeks to 12 weeks

Outcome	Group	6 weeks Mean (SD)	12 wks Mean (SD)	Change Mean (SD) [range]	% Change Mean (SD) [range]	Group Difference (*) Mean (95% CI)	P-value
TC	Placebo	5.78 (1.02)	5.53 (0.71)	-0.25 (0.44) [-0.9, 0]	-3.7 (6.2) [-12.9, 0.0]	0	
	Active	4.93 (0.58)	4.99 (0.71)	0.06 (0.65) [-1.3, 1]	1.7 (11.9) [-22.4, 18.9]	0.00 (-0.90, 0.90)	0.99
HDL	Placebo	1.65 (0.17)	1.38 (0.13)	-0.28 (0.05) [-0.3, -0.2]	-16.6 (1.6) [-17.6, -14.3]	0	
	Active	1.69 (0.42)	1.69 (0.41)	0.00 (0.15) [-0.2, 0.2]	0.5 (8.8) [-12.5, 14.3]	0.28 (0.10, 0.46)	**0.007**
LDL	Placebo	3.80 (0.91)	3.67 (0.56)	-0.12 (0.45) [-0.8, 0.1]	-1.8 (9.5) [-16.0, 3.6]	0	
	Active	2.85 (0.40)	2.90 (0.65)	0.05 (0.53) [-1.1, 0.8]	1.9 (16.6) [-34.4, 24.2]	-0.12 (-1.02, 0.77)	0.76
Trigs.	Placebo	0.75 (0.10)	1.05 (0.17)	0.30 (0.14) [0.1, 0.4]	40.5 (20.8) [14.3, 57.1]	0	
	Active	0.84 (0.55)	0.85 (0.42)	0.01 (0.24) [-0.4, 0.3]	7.5 (28.1) [-33.3, 42.9]	-0.26 (-0.50, -0.03)	**0.03**

(*) Calculated from ANCOVA analysis, adjusting for 6 week value

Significant differences between groups for the period 6 to 12 weeks were observed for both HDL cholesterol and triglycerides. HDL values were significantly higher in the active group, on average by 0.28 mol/L. Conversely, triglyceride values were significantly lower in the active group, on average by 0.26 units.

The two groups did not differ in terms of total and LDL cholesterol.

Table 40: Change in Lipid measurements in subjects aged 60 and over from baseline to 12 weeks

Outcome	Group	Baseline Mean (SD)	12 weeks Mean (SD)	Change Mean (SD) [range]	% Change Mean (SD) [range]	Group Difference (*) Mean (95% CI)	P-value
TC	Placebo	5.65 (1.01)	5.53 (0.71)	-0.12 (0.80) [-1.0, 0.9]	-1.1 (14.0) [-14.1, 18.0]	0	
	Active	5.09 (0.70)	4.99 (0.71)	-0.10 (0.52) [-1.3, 0.3]	-1.5 (9.3) [-22.4, 7.0]	-0.20 (-1.01, 0.60)	0.58
HDL	Placebo	1.52 (0.10)	1.38 (0.13)	-0.15 (0.10) [-0.2, 0.0]	-9.8 (6.6) [-14.3, 0.0]	0	
	Active	1.61 (0.38)	1.69 (0.41)	0.08 (0.15) [-0.1, 0.3]	4.7 (9.6) [-6.7, 21.4]	0.23 (0.02, 0.43)	**0.03**
LDL	Placebo	3.78 (0.90)	3.67 (0.56)	-0.1 (0.67) [-0.9, 0.7]	-0.6 (17.2) [-17.6, 21.9]	0	
	Active	3.01 (0.56)	2.90 (0.65)	-0.11 (0.47) [-1.2, 0.3]	-3.1 (14.9) [-36.4, 13.0]	-0.31 (-1.10, 0.48)	0.40
Trigs.	Placebo	0.73 (0.15)	1.05 (0.17)	0.33 (0.13) [0.2, 0.5]	46.9 (24.4) [33.3, 83.3]	0	
	Active	1.06 (0.45)	0.85 (0.42)	-0.21 (0.19) [-0.5, 0.0]	-19.6 (19.5) [-55.6, 0.0]	-0.48 (-0.74, -0.23)	**0.002**

(*) Calculated from ANCOVA analysis, adjusting for baseline value

As with the period from 6 to 12 weeks, there were significant group differences for the period from baseline to 12 weeks. Again, HDL values were higher in the active group, with triglyceride values lower.

Table 41: Change in Lipid measurements in subjects aged 60 and over from 12 weeks to 16 weeks

Outcome	Group	12 weeks Mean (SD)	16 weeks Mean (SD)	Change Mean (SD) [range]	% Change Mean (SD) [range]	Group Difference (*) Mean (95% CI)	P-value
TC	Placebo	5.53 (0.71)	5.30 (0.55)	-0.22 (0.56) [-0.9, 0.4]	-3.4 (10.3) [-15.3, 8.9]	0	
	Active	4.99 (0.71)	5.15 (0.71)	0.16 (0.42) [-0.5, 0.6]	3.6 (8.3) [-8.7, 13.3]	0.24 (-0.43, 0.90)	0.44
HDL	Placebo	1.38 (0.13)	1.45 (0.10)	0.08 (0.1) [0.0, 0.2]	5.8 (7.9) [0.0, 16.7]	0	
	Active	1.69 (0.41)	1.69 (0.43)	0.00 (0.16) [-0.3, 0.2]	-0.2 (9.6) [-14.3, 14.3]	-0.06 (-0.29, 0.17)	0.56
LDL	Placebo	3.67 (0.56)	3.45 (0.57)	-0.23 (0.46) [-0.8, 0.2]	-5.5 (12.5) [-20.5, 6.9]	0	
	Active	2.90 (0.65)	3.05 (0.56)	0.15 (0.43) [-0.5, 0.9]	7.0 (17.2) [-12.2, 42.9]	0.11 (-0.56, 0.78)	0.73
Trigs.	Placebo	1.05 (0.17)	0.88 (0.21)	-0.17 (0.25) [-0.5, 0.1]	-14.9 (24.1) [-45.5, 12.5]	0	
	Active	0.85 (0.42)	0.90 (0.61)	0.05 (0.27) [-0.2, 0.6]	5.8 (32.8) [-25.0, 75.0]	0.28 (-0.08, 0.65)	0.12

(*) Calculated from ANCOVA analysis, adjusting for 12 week value

There were no statistically significant differences in lipid measurements between the two groups for the period for 12 to 16 weeks.

Introduction

A randomised control trial was performed to examine the cholesterol lowering efficacy of a *Lactobacillus* strain in hypercholesterolaemic adults. Subjects were randomised using a 1:1 allocation into either a placebo group (maltodexrin) or an active group (*Lactobacillus* strain).

Each study participant was measured on 4 occasions:

- 0 weeks (Baseline)
- 6 weeks (During treatment)
- 12 weeks (End of treatment)
- 16 weeks (Post-treatment)

This report details an analysis of the assay outcomes.

Statistical Methods

The methods of analysis were as described in previous reports.

The first set of analyses considered all study participants in the analysis. Subsequently, the change in outcome between baseline and 12 weeks only were repeated for different patient subgroups, with subgroups created based on baseline total cholesterol, gender and age.

Results

a) All participants combined

Analyses were performed to examine how the changes in assay measurements over the course of the study varied between the two study groups. Initially the analyses were performed for all subjects combined.

The changes between four pairs of timepoints were examined, with results summarised in Tables 1-4. These tables show the mean and standard deviation at each of the timepoints, and also in terms of the change between timepoints. The group differences from the ANCOVA analyses are also reported, with the figures the mean difference and corresponding confidence interval. These are reported as outcome for Active group minus outcome for Placebo group. [Note that due to the nature of the analyses the mean group difference may not equate to the difference in raw changes over time between groups]. P-values indicating the significance of the results are also reported.

Table 1: Change in Assay measurements in all subjects from baseline to 6 weeks

Outcome	Group	Baseline Mean (SD)	6 weeks Mean (SD)	Change Mean (SD) [range]	% Change Mean (SD) [range]	Group Difference (*) Mean (95% CI)	P-value
IL-6 (#)	Placebo	54.61 (1.07)	54.56 (1.03)	-0.05 (0.57) [-1.14, 2.01]	-0.1 (1) [-2, 3.7]	0	
	Active	54.63 (0.92)	54.61 (0.92)	-0.01 (0.13) [-0.41, 0.17]	0.0 (0.2) [-0.7, 0.3]	0.04 (-0.20, 0.28)	0.76
IL-10 (#)	Placebo	74.22 (25.49)	74.20 (25.48)	-0.02 (0.10) [-0.27, 0.25]	0 (0.2) [-0.5, 0.4]	0	
	Active	66.26 (23.08)	66.29 (23.05)	0.03 (0.19) [-0.24, 0.61]	0.1 (0.4) [-0.5, 1.2]	0.04 (-0.06, 0.14)	0.41
TNF-α (#)	Placebo	113.7 (5.24)	113.7 (5.23)	-0.01 (0.03) [-0.1, 0.05]	0.0 (0.0) [-0.1, 0.0]	0	
	Active	112.3 (4.93)	112.3 (4.93)	-0.01 (0.04) [-0.15, 0.08]	0.0 (0.0) [-0.1, 0.1]	0.00 (-0.02, 0.02)	0.74
Glucose	Placebo	5.4 (0.6)	5.4 (0.8)	-0.1 (0.6) [-2, 0.7]	-1.2 (10.1) [-35.6, 13.2]	0	
	Active	5.2 (0.6)	5.1 (0.6)	-0.1 (0.5) [-1.2, 1.1]	-0.9 (9.8) [-22.9, 24.4]	0.0 (-0.4, 0.3)	0.76
CRP	Placebo	4.0 (7.4)	2.1 (2.2)	-1.9 (6.7) [-28.2, 5.3]	188 (565) [-91, 2076]	0	
	Active	3.1 (4.6)	2.8 (4.2)	-0.3 (4.3) [-12.3, 14.6]	175 (790) [-66, 3777]	1.0 (-0.9, 2.8)	0.30

(*) Calculated from ANCOVA analysis, adjusting for baseline value
(#) Summary statistics reported in thousands

There was no evidence of that the two groups varied in terms of changes from baseline to 6 weeks.

Table 2: Change in Assay measurements in all subjects from 6 weeks to 12 weeks

Outcome	Group	6 weeks Mean (SD)	12 weeks Mean (SD)	Change Mean (SD) [range]	% Change Mean (SD) [range]	Group Difference (*) Mean (95% CI)	P-value
IL-6 (#)	Placebo	54.56 (1.03)	54.52 (1.01)	-0.04 (0.49) [-1.93, 0.82]	-0.1 (0.9) [-3.4, 1.5]	0	
	Active	54.61 (0.92)	54.68 (0.98)	0.07 (0.18) [-0.29, 0.69]	0.1 (0.3) [-0.5, 1.2]	0.12 (-0.11, 0.34)	0.30
IL-10 (#)	Placebo	73.15 (25.4)	73.16 (25.42)	0.01 (0.08) [-0.17, 0.21]	0.0 (0.1) [-0.3, 0.2]	0	
	Active	66.29 (23.05)	66.3 (23.07)	0.01 (0.13) [-0.34, 0.43]	0.0 (0.2) [-0.7, 0.7]	0.01 (-0.05, 0.07)	0.70
TNF-α (#)	Placebo	113.7 (5.23)	113.7 (5.23)	0.01 (0.03) [-0.05, 0.13]	0.0 (0.0) [0, 0.1]	0	
	Active	112.3 (4.93)	112.4 (4.93)	0.03 (0.07) [-0.04, 0.3]	0.0 (0.1) [0, 0.3]	0.02 (-0.02, 0.05)	0.38
Glucose	Placebo	5.4 (0.8)	5.3 (0.7)	0.0 (0.5) [-1.0, 1.2]	0.3 (10.8) [-17, 34.8]	0	
	Active	5.1 (0.6)	5.1 (0.5)	-0.1 (0.5) [-0.7, 1.5]	-0.5 (10.5) [-14.2, 36.2]	-0.1 (-0.4, 0.2)	0.38
CRP	Placebo	2.1 (2.2)	1.6 (2.0)	-0.5 (1.6) [-5.3, 2.2]	-13.5 (62.6) [-99, 103]	0	
	Active	2.8 (4.2)	2.2 (2.6)	-0.7 (4) [-18.5, 2.6]	10.1 (79.1) [-95, 237]	0.3 (-0.9, 21.7)	0.59

(*) Calculated from ANCOVA analysis, adjusting for baseline value
(#) Summary statistics reported in thousands

No differences between groups were observed in terms of the change from 6 to 12 weeks.

Table 3: Change in Assay measurements in all subjects from baseline to 12 weeks

Outcome	Group	Baseline Mean (SD)	12 weeks Mean (SD)	Change Mean (SD) [range]	% Change Mean (SD) [range]	Group Difference (*) Mean (95% CI)	P-value
IL-6 (#)	Placebo	54.61 (1.07)	54.52 (1.01)	-0.09 (0.47) [-1.44, 0.82]	-0.2 (0.8) [-2.6, 1.5]	0	
	Active	54.63 (0.92)	54.68 (0.98)	0.06 (0.26) [-0.69, 0.73]	0.1 (0.5) [-1.3, 1.3]	0.15 (-0.07, 0.37)	0.18
IL-10 (#)	Placebo	74.22 (25.49)	74.21 (25.5)	-0.01 (0.12) [-0.44, 0.22]	0.0 (0.2) [-0.8, 0.2]	0	
	Active	66.26 (23.08)	66.3 (23.07)	0.04 (0.24) [-0.23, 1.04]	0.1 (0.4) [-0.5, 1.8]	0.05 (-0.07, 0.17)	0.38
TNF-α (#)	Placebo	113.7 (5.24)	113.7 (5.23)	0.00 (0.03) [-0.06, 0.05]	0.0 (0.0) [-0.1, 0.0]	0	
	Active	112.3 (4.93)	112.4 (4.93)	0.02 (0.08) [-0.07, 0.38]	0.0 (0.1) [-0.1, 0.3]	0.02 (-0.02, 0.06)	0.31
Glucose	Placebo	5.4 (0.6)	5.3 (0.7)	-0.1 (0.4) [-0.7, 0.8]	-1.7 (8.1) [-13.2, 16.4]	0	
	Active	5.2 (0.6)	5.1 (0.5)	-0.1 (0.4) [-0.9, 0.9]	-1.9 (8.5) [-16, 20.7]	-0.1 (-0.3, 0.2)	0.57
CRP	Placebo	4.0 (7.4)	1.6 (2.0)	-2.4 (6.1) [-26, 1.3]	-11 (67.8) [-94.9, 121]	0	
	Active	3.1 (4.6)	2.2 (2.6)	-0.9 (2.9) [-13.1, 1.4]	69 (341) [-82, 1604]	0.8 (-0.2, 1.8)	0.11

(*) Calculated from ANCOVA analysis, adjusting for baseline value
(#) Summary statistics reported in thousands

There were no statistically significant differences between the two study groups for the change from baseline to 12 weeks.

Table 4: Change in Assay measurements in all subjects from 12 weeks to 16 weeks

Outcome	Group	12 weeks Mean (SD)	16 weeks Mean (SD)	Change Mean (SD) [range]	% Change Mean (SD) [range]	Group Difference (*) Mean (95% CI)	P-value
IL-6 (#)	Placebo	54.47 (1.00)	54.51 (1.00)	0.04 (0.25) [-0.51, 0.5]	0.1 (0.5) [-0.9, 0.9]	0	
	Active	54.68 (0.98)	54.72 (1.05)	0.04 (0.32) [-0.99, 1.02]	0.1 (0.6) [-1.8, 1.8]	0.00 (-0.18, 0.18)	0.99
IL-10 (#)	Placebo	73.16 (25.42)	73.17 (25.39)	0.01 (0.11) [-0.2, 0.23]	0 (0.2) [-0.3, 0.4]	0	
	Active	66.3 (23.07)	66.32 (23.05)	0.02 (0.1) [-0.36, 0.24]	0 (0.2) [-0.6, 0.5]	0.00 (-0.06, 0.06)	0.99
TNF-α (#)	Placebo	113.7 (5.23)	113.7 (5.24)	0.00 (0.04) [-0.11, 0.14]	0.0 (0.0) [-0.1, 0.1]	0	
	Active	112.4 (4.93)	112.4 (4.93)	-0.01 (0.07) [-0.21, 0.2]	0.0 (0.1) [-0.2, 0.2]	-0.01 (-0.05, 0.02)	0.44
Glucose	Placebo	5.3 (0.7)	5.4 (0.6)	0.1 (0.3) [-0.4, 0.7]	1.9 (6.1) [-7.7, 14.6]	0	
	Active	5.1 (0.5)	5.2 (0.5)	0.1 (0.5) [-0.8, 1.2]	3.1 (10.1) [-15.1, 28.4]	0.0 (-0.3, 0.2)	0.79
CRP	Placebo	1.6 (2.0)	1.8 (2.3)	0.2 (1.6) [-3, 5.8]	214.7 (683) [-98, 3184]	0	
	Active	2.2 (2.6)	4.1 (6.1)	1.9 (5.8) [-2.3, 23.8]	258.7 (680) [-98, 2671]	1.8 (-0.8, 4.3)	0.17

(*) Calculated from ANCOVA analysis, adjusting for baseline value
(#) Summary statistics reported in thousands

There was no evidence of a difference in outcome measurements between the two groups for the period for 12 to 16 weeks.

b) Total cholesterol subgroups

The next analyses concentrated on the subgroups based on the baseline total cholesterol values. Analyses were performed to examine how the changes in lipid measurements over from baseline varied between the two study groups. The changes between these timepoints were examined for each subgroup, with results summarised in Tables 5-7.

Table 5: Change in Assay measurements in subjects with baseline TC < 5.0 mmol/L from baseline to 12 weeks

Outcome	Group	Baseline Mean (SD)	12 weeks Mean (SD)	Change Mean (SD) [range]	% Change Mean (SD) [range]	Group Difference (*) Mean (95% CI)	P-value
IL-6 (#)	Placebo	54.77 (1.21)	54.62 (1.11)	-0.15 (0.46) [-1.44, 0.19]	-0.3 (0.8) [-2.6, 0.3]	0	
	Active	54.62 (0.99)	54.68 (1.08)	0.06 (0.28) [-0.69, 0.48]	0.1 (0.5) [-1.3, 0.9]	0.20 (-0.14, 0.54)	0.23
IL-10 (#)	Placebo	69.71 (23.87)	69.65 (23.87)	-0.07 (0.14) [-0.44, 0.03]	-0.1 (0.2) [-0.8, 0.1]	0	
	Active	61.64 (20.02)	61.7 (20.03)	0.06 (0.31) [-0.14, 1.04]	0.1 (0.6) [-0.3, 1.8]	0.13 (-0.11, 0.36)	0.27
TNF-α (#)	Placebo	113.0 (4.78)	113.0 (4.77)	0.00 (0.03) [-0.06, 0.05]	0.0 (0.0) [-0.1, 0]	0	
	Active	111.6 (4.47)	111.6 (4.48)	0.03 (0.11) [-0.07, 0.38]	0.0 (0.1) [-0.1, 0.3]	0.03 (-0.04, 0.11)	0.38
Glucose	Placebo	5.6 (0.5)	5.4 (0.7)	-0.2 (0.4) [-0.7, 0.4]	-3.4 (7.6) [-13.2, 7.9]	0	
	Active	5.2 (0.5)	5 (0.5)	-0.1 (0.4) [-0.9, 0.6]	-2.2 (7.1) [-14.4, 11.4]	0.0 (-0.4, 0.4)	0.97
CRP	Placebo	1.2 (1.4)	0.9 (1.0)	-0.4 (0.8) [-2.3, 0.9]	-20.6 (51) [-95, 86]	0	
	Active	4.2 (6.1)	2.9 (3.5)	-1.3 (3.9) [-13.1, 1.4]	125 (470) [-82, 160]	0.7 (-0.8, 1.3)	0.36

(*) Calculated from ANCOVA analysis, adjusting for baseline value
(#) Summary statistics reported in thousands

Table 6: Change in Assay measurements in subjects with baseline TC between 5.0-5.9 mmol/L from baseline to 12 weeks

Outcome	Group	Baseline Mean (SD)	12 weeks Mean (SD)	Change Mean (SD) [range]	% Change Mean (SD) [range]	Group Difference (*) Mean (95% CI)	P-value
IL-6 (#)	Placebo	54.62 (0.98)	54.57 (0.97)	-0.05 (0.56) [-1.04, 0.82]	-0.1 (1.0) [-1.9, 1.5]	0	
	Active	54.55 (0.93)	54.64 (0.98)	0.1 (0.27) [-0.15, 0.73]	0.2 (0.5) [-0.3, 1.3]	0.14 (-0.34, 0.62)	0.54
IL-10 (#)	Placebo	80.62 (27.7)	80.63 (27.7)	0.01 (0.05) [-0.09, 0.07]	0 (0.1) [-0.2, 0.1]	0	
	Active	71.74 (26.82)	71.73 (26.8)	-0.01 (0.13) [-0.23, 0.21]	0 (0.3) [-0.5, 0.4]	-0.02 (-0.13, 0.09)	0.71
TNF-α (#)	Placebo	114.7 (5.98)	114.7 (5.97)	0.00 (0.03) [-0.04, 0.04]	0.0 (0.0) [0.0, 0.0]	0	
	Active	113.2 (5.66)	113.2 (5.66)	0.01 (0.01) [-0.01, 0.03]	0.0 (0.0) [0.0, 0.0]	0.00 (-0.02, 0.02)	0.97
Glucose	Placebo	5.4 (0.6)	5.4 (0.8)	0 (0.5) [-0.6, 0.8]	0.5 (9.8) [-12.2, 16.4]	0	
	Active	5.4 (0.6)	5.2 (0.6)	-0.2 (0.4) [-0.9, 0.4]	-3.6 (8.1) [-16, 8.3]	-0.2 (-0.8, 0.3)	0.35
CRP	Placebo	8.4 (10.6)	2.8 (2.6)	-5.6 (9.1) [-26, 1.3]	-8.9 (83) [-93, 121]	0	
	Active	1.4 (1.3)	1.4 (0.9)	-0.1 (0.7) [-1.5, 0.9]	23.8 (78) [-37, 155]	- 0.3 (-2.1, 1.5)	0.74

(*) Calculated from ANCOVA analysis, adjusting for baseline value
(#) Summary statistics reported in thousands

Table 7: Change in Assay measurements in subjects with baseline TC of ≥6.0 mmol/L from baseline to 12 weeks

Outcome	Group	Baseline Mean (SD)	12 weeks Mean (SD)	Change Mean (SD) [range]	% Change Mean (SD) [range]	Group Difference (*) Mean (95% CI)	P-value
IL-6 (#)	Placebo	54 (0.99)	54.01 (0.93)	0.01 (0.09) [-0.07, 0.11]	0 (0.2) [-0.1, 0.2]	0	
	Active	54.85 (0.85)	54.81 (0.86)	-0.04 (0.12) [-0.12, 0.09]	-0.1 (0.2) [-0.2, 0.2]	-0.02 (-0.06, 0.32)	0.86
IL-10 (#)	Placebo	70.03 (29.56)	70.16 (29.65)	0.13 (0.08) [0.06, 0.22]	0.2 (0.1) [0.1, 0.2]	0	
	Active	70.09 (29.48)	70.2 (29.42)	0.11 (0.13) [0, 0.26]	0.2 (0.3) [0, 0.5]	-0.02 (-0.35, 0.31)	0.85
TNF-α (#)	Placebo	113.1 (6.13)	113.1 (6.1)	-0.01 (0.03) [-0.05, 0.00]	0.0 (0.0) [0.0, 0.0]	0	
	Active	113.1 (6.13)	113.1 (6.13)	0.01 (0.00) [0.00, 0.01]	0.0 (0.0) [0.0, 0.0]	0.02 (-0.03, 0.07)	0.30
Glucose	Placebo	5.2 (0.6)	5.1 (0.6)	-0.1 (0.2) [-0.3, 0.0]	-2.1 (3.4) [-6, 0.2]	0	
	Active	4.9 (0.7)	5 (0.5)	0.1 (0.7) [-0.4, 0.9]	3.5 (15.2) [-7.9, 20.7]	0.1 (-1.3, 1.4)	0.89
CRP	Placebo	0.4 (0.3)	0.7 (0.8)	0.2 (0.5) [-0.2, 0.8]	18 (91) [-81, 97]	0	
	Active	3.4 (1.7)	1.7 (0.7)	-1.8 (2.1) [-3.9, 0.2]	-36 (48) [-82, 14]	1.2 (-3.0, 5.4)	0.43

(*) Calculated from ANCOVA analysis, adjusting for baseline value
(#) Summary statistics reported in thousands

There were no statistically significant differences between the two study groups for the change from baseline to 12 weeks for any of the three subgroups based on total cholesterol.

c) Gender subgroups

The next set of analyses compared the change from baseline to 12 weeks by gender. The results summarised in Tables 8 and9.

Table 8: Change in Assay measurements in females from baseline to 12 weeks

Outcome	Group	Baseline Mean (SD)	12 weeks Mean (SD)	Change Mean (SD) [range]	% Change Mean (SD) [range]	Group Difference (*) Mean (95% CI)	P-value
IL-6 (#)	Placebo	54.73 (1.12)	54.54 (0.98)	-0.19 (0.51) [-1.44, 0.68]	-0.3 (0.9) [-2.6, 1.2]	0	
	Active	54.40 (0.88)	54.47 (0.98)	0.07 (0.29) [-0.69, 0.73]	0.1 (0.5) [-1.3, 1.3]	0.22 (-0.07, 0.51)	0.13
IL-10 (#)	Placebo	76.45 (26.65)	76.44 (26.67)	-0.01 (0.15) [-0.44, 0.22]	0.0 (0.2) [-0.8, 0.2]	0	
	Active	66.28 (24.19)	66.28 (24.19)	0.00 (0.12) [-0.23, 0.26]	0.0 (0.2) [-0.5, 0.5]	0.01 (-0.09, 0.14)	0.83
TNF-α (#)	Placebo	113.7 (5.63)	113.7 (5.62)	0.00 (0.03) [-0.05, 0.04]	0.0 (0.0) [0.0, 0.0]	0	
	Active	111.9 (5.31)	111.9 (5.31)	0.03 (0.09) [-0.07, 0.38]	0.0 (0.1) [-0.1, 0.3]	0.02 (-0.03, 0.08)	0.37
Glucose	Placebo	5.5 (0.7)	5.4 (0.7)	0.0 (0.4) [-0.7, 0.8]	-0.2 (8.2) [-11.1, 16.4]	0	
	Active	5.2 (0.6)	5.1 (0.6)	-0.1 (0.5) [-0.9, 0.9]	-2.2 (9.4) [-16, 20.7]	-0.2 (-0.5, 0.1)	0.25
CRP	Placebo	5.7 (9.2)	1.9 (2.3)	-3.7 (7.7) [-26, 1.3]	-13.1 (70) [-93, 121]	0	
	Active	3.5 (5)	2.5 (2.9)	-1.0 (3.2) [-13.1, 1.4]	890 (383) [-75, 1604]	1.1 (-0.3, 2.5)	0.11

(*) Calculated from ANCOVA analysis, adjusting for baseline value
(#) Summary statistics reported in thousands

Table 9: Change in Assay measurements in males from baseline to 12 weeks

Outcome	Group	Baseline Mean (SD)	12 weeks Mean (SD)	Change Mean (SD) [range]	% Change Mean (SD) [range]	Group Difference (*) Mean (95% CI)	P-value
IL-6 (#)	Placebo	54.39 (1.01)	54.48 (1.13)	0.08 (0.33) [-0.29, 0.82]	0.2 (0.6) [-0.5, 1.5]	0	
	Active	55.43 (0.54)	55.46 (0.45)	0.03 (0.11) [-0.09, 0.19]	0.1 (0.2) [-0.2, 0.3]	-0.09 (-0.51, 0.33)	0.65
IL-10 (#)	Placebo	71.00 (24.91)	70.99 (24.91)	-0.01 (0.07) [-0.1, 0.11]	0.0 (0.1) [-0.2, 0.2]	0	
	Active	66.16 (21.07)	66.37 (20.97)	0.21 (0.46) [-0.01, 1.04]	0.4 (0.8) [0.0, 1.8]	0.21 (-0.14, 0.56)	0.21
TNF-α (#)	Placebo	113.7 (4.9)	113.7 (4.89)	0.00 (0.03) [-0.06, 0.05]	0.0 (0.0) [-0.1, 0.0]	0	
	Active	113.9 (3.18)	113.9 (3.18)	0.01 (0.02) [-0.02, 0.04]	0.0 (0.0) [0.0, 0.0]	0.01 (-0.03, 0.04)	0.71
Glucose	Placebo	5.4 (0.4)	5.2 (0.7)	-0.2 (0.4) [-0.7, 0.3]	-4.1 (7.8) [-13.2, 5.3]	0	
	Active	5.2 (0.5)	5.1 (0.5)	-0.1 (0.2) [-0.3, 0.3]	-1.1 (4.6) [-5.4, 4.9]	0.2 (-0.3, 0.7)	0.40
CRP	Placebo	1.3 (1.7)	1.1 (1.3)	-0.2 (0.6) [-1.4, 0.9]	-7.7 (68.6) [-94.9, 106]	0	
	Active	1.7 (1.8)	1.0 (0.7)	-0.6 (1.9) [-3.9, 1.1]	-6.4 (95.3) [-82.4, 151]	-0.2 (-1.2, 0.8)	0.66

(*) Calculated from ANCOVA analysis, adjusting for baseline value
(#) Summary statistics reported in thousands

There were no statistically significant differences between the two study groups for the change from baseline to 12 weeks for either males or females

d) Age subgroups

Analyses were performed for subgroup of subjects split by their age. The results summarised in Tables 10-12.

Table 10: Change in Assay measurements in subjects aged less than 50 from baseline to 12 weeks

Outcome	Group	Baseline Mean (SD)	12 weeks Mean (SD)	Change Mean (SD) [range]	% Change Mean (SD) [range]	Group Difference (*) Mean (95% CI)	P-value
IL-6 (#)	Placebo	54.84 (1.21)	54.71 (1.12)	-0.14 (0.17) [-0.36, 0.02]	-0.2 (0.3) [-0.6, 0.0]	0	
	Active	54.71 (1.07)	54.74 (1.05)	0.03 (0.07) [-0.09, 0.12]	0.1 (0.1) [-0.2, 0.2]	0.16 (0.03, 0.29)	**0.02**
IL-10 (#)	Placebo	71.56 (25.35)	71.46 (25.36)	-0.10 (0.17) [-0.44, 0.01]	-0.2 (0.3) [-0.8, 0.0]	0	
	Active	64.85 (22.4)	64.81 (22.42)	-0.04 (0.1) [-0.23, 0.08]	-0.1 (0.2) [-0.5, 0.1]	0.06 (-0.09, 0.22)	0.39
TNF-α (#)	Placebo	113.0 (5.04)	113.0 (5.02)	-0.01 (0.03) [-0.06, 0.04]	0.0 (0.0) [-0.1, 0.0]	0	
	Active	112.4 (4.73)	112.4 (4.73)	0.01 (0.02) [-0.02, 0.05]	0.0 (0.0) [0.0, 0.0]	0.01 (-0.01, 0.04)	0.28
Glucose	Placebo	5.5 (0.3)	5.2 (0.4)	-0.2 (0.5) [-0.7, 0.7]	-3.8 (8.7) [-13.2, 14.7]	0	
	Active	5.0 (0.4)	4.9 (0.5)	-0.1 (0.4) [-0.5, 0.6]	-1.7 (7.3) [-10.2, 11.4]	-0.0 (-0.6, 0.5)	0.86
CRP	Placebo	7.8 (12.2)	2.1 (2.8)	-5.7 (9.8) [-26, 0.1]	-40.9 (45.3) [-95, 23.8]	0	
	Active	2.8 (4.0)	2.2 (3.0)	-0.6 (1.7) [-3.9, 1.1]	36.4 (88.6) [-81.5, 155]	1.5 (-0.8, 3.7)	0.18

(*) Calculated from ANCOVA analysis, adjusting for baseline value
(#) Summary statistics reported in thousands

There were a statistically significant differences between the two study groups for the <50 subgroup for the change from baseline to 12 weeks in terms of IL-6. There was a decrease in values over time in the placebo group, wand a slight increase in the active group. The mean difference between groups was 0.16 $x10^{-3}$ pg/ml

Table 11: Change in Assay measurements in subjects aged 50-59 from baseline to 12 weeks

Outcome	Group	Baseline Mean (SD)	12 weeks Mean (SD)	Change Mean (SD) [range]	% Change Mean (SD) [range]	Group Difference (*) Mean (95% CI)	P-value
IL-6 (#)	Placebo	54.35 (1.02)	54.28 (0.95)	-0.08 (0.51) [-1.44, 0.82]	-0.1 (0.9) [-2.6, 1.5]	0	
	Active	54.08 (0.55)	54.03 (0.61)	-0.05 (0.36) [-0.69, 0.36]	-0.1 (0.7) [-1.3, 0.7]	-0.02 (-0.51, 0.47)	0.93
IL-10 (#)	Placebo	74.12 (26.46)	74.15 (26.48)	0.03 (0.09) [-0.09, 0.22]	0 (0.1) [-0.2, 0.2]	0	
	Active	67.71 (28.06)	67.74 (28.01)	0.04 (0.10) [-0.07, 0.21]	0.1 (0.2) [-0.1, 0.4]	0.01 (-0.09, 0.11)	0.86
TNF-α (#)	Placebo	113.7 (5.55)	113.7 (5.53)	0.00 (0.02) [-0.05, 0.03]	0.0 (0.0) [0.0, 0.0]	0	
	Active	111.4 (6.29)	111.5 (6.29)	0.01 (0.02) [-0.01, 0.03]	0.0 (0.0) [0.0, 0.0]	0.01 (-0.01, 0.03)	0.50
Glucose	Placebo	5.6 (0.7)	5.6 (0.8)	-0.1 (0.4) [-0.7, 0.4]	-1.1 (6.9) [-12.2, 7.9]	0	
	Active	5.4 (0.6)	5.2 (0.7)	-0.2 (0.2) [-0.4, 0.1]	-4.2 (3.4) [-8.2, 1.9]	-0.2 (-0.5, 0.2)	0.36
CRP	Placebo	1.7 (2.3)	1.3 (1.7)	-0.4 (0.7) [-1.8, 0.2]	-14.8 (58) [-85, 106]	0	
	Active	5.9 (7.0)	3.3 (3.4)	-2.5 (5.2) [-13.1, 0.3]	-35.5 (34.7) [-75, 10.9]	0.3 (-1.5, 2.2)	0.71

(*) Calculated from ANCOVA analysis, adjusting for baseline value
(#) Summary statistics reported in thousands

No significant differences between groups were observed in the 50-59 age category

Table 12: Change in Assay measurements in subjects aged 60 and over from baseline to 12 weeks

Outcome	Group	Baseline Mean (SD)	12 weeks Mean (SD)	Change Mean (SD) [range]	% Change Mean (SD) [range]	Group Difference (*) Mean (95% CI)	P-value
IL-6 (#)	Placebo	55.01 (1.11)	54.95 (1.06)	-0.06 (0.72) [-1.04, 0.68]	-0.1 (1.3) [-1.9, 1.2]	0	
	Active	54.94 (0.87)	55.11 (0.94)	0.17 (0.29) [-0.12, 0.73]	0.3 (0.5) [-0.2, 1.3]	0.23 (-0.44, 0.89)	0.46
IL-10 (#)	Placebo	78.5 (29.48)	78.52 (29.46)	0.02 (0.04) [-0.04, 0.06]	0 (0.1) [0, 0.1]	0	
	Active	66.75 (23.11)	66.89 (23.06)	0.14 (0.38) [-0.14, 1.04]	0.2 (0.7) [-0.2, 1.8]	0.11 (-0.37, 0.58)	0.62
TNF-α (#)	Placebo	114.7 (5.95)	114.8 (5.96)	0.03 (0.02) [0.00, 0.05]	0.0 (0.0) [0.0, 0.0]	0	
	Active	113.0 (4.63)	113.0 (4.63)	0.05 (0.14) [-0.07, 0.38]	0.0 (0.1) [-0.1, 0.3]	0.02 (-0.16, 0.19)	0.83
Glucose	Placebo	4.9 (0.4)	4.9 (0.4)	0.0 (0.6) [-0.6, 0.8]	0.1 (11.9) [-12, 16.4]	0	
	Active	5.2 (0.6)	5.2 (0.4)	-0.1 (0.6) [-0.9, 0.9]	-0.5 (12.3) [-16, 20.7]	-0.2 (-0.5, 0.9)	0.54
CRP	Placebo	3.8 (5.7)	1.7 (0.7)	-2.2 (6.3) [-11.6, 1.3]	52.8 (98.6) [-93.4, 121]	0	
	Active	1.4 (1.4)	1.3 (0.8)	-0.1 (0.9) [-1.7, 1.4]	184 (575) [-82.4, 1604]	-0.5 (-1.7, 0.7)	0.36

(*) Calculated from ANCOVA analysis, adjusting for baseline value
(#) Summary statistics reported in thousands

No significant differences between groups were observed in the over 60 year age category
